# Basicity as a Thermodynamic Descriptor of Carbanions Reactivity with Carbon Dioxide: Application to the Carboxylation of α,β-Unsaturated Ketones

**DOI:** 10.3389/fchem.2021.783993

**Published:** 2021-11-24

**Authors:** Pietro Franceschi, Catia Nicoletti, Ruggero Bonetto, Marcella Bonchio, Mirco Natali, Luca Dell’Amico, Andrea Sartorel

**Affiliations:** ^1^ Nano and Molecular Catalysis Laboratory, Department of Chemical Sciences, University of Padova, Padova, Italy; ^2^ Department of Chemical, Pharmaceutical and Agricultural Sciences (DOCPAS), University of Ferrara, and Centro Interuniversitario per La Conversione Chimica Dell’Energia Solare (SOLARCHEM), Ferrara, Italy

**Keywords:** carbon dioxide fixation, thermodynamic analysis, DFT calculations, reaction intermediates, unsaturated carbonyl

## Abstract

The utilization of carbon dioxide as a raw material represents nowadays an appealing strategy in the renewable energy, organic synthesis, and green chemistry fields. Besides reduction strategies, carbon dioxide can be exploited as a single-carbon-atom building block through its fixation into organic scaffolds with the formation of new C-C bonds (carboxylation processes). In this case, activation of the organic substrate is commonly required, upon formation of a carbanion C^−^, being sufficiently reactive toward the addition of CO_2_. However, the prediction of the reactivity of C^−^ with CO_2_ is often problematic with the process being possibly associated with unfavorable thermodynamics. In this contribution, we present a thermodynamic analysis combined with density functional theory calculations on 50 organic molecules enabling the achievement of a linear correlation of the standard free energy (ΔG^0^) of the carboxylation reaction with the basicity of the carbanion C^−^, expressed as the pK_a_ of the CH/C^−^ couple. The analysis identifies a threshold pK_a_ of ca 36 (in CH_3_CN) for the CH/C^−^ couple, above which the ΔG^0^ of the carboxylation reaction is negative and indicative of a favorable process. We then apply the model to a real case involving electrochemical carboxylation of flavone and chalcone as model compounds of α,β-unsaturated ketones. Carboxylation occurs in the β-position from the doubly reduced dianion intermediates of flavone and chalcone (calculated ΔG^0^ of carboxylation in β = −12.8 and −20.0 Kcalmol^-1^ for flavone and chalcone, respectively, associated with pK_a_ values for the conjugate acids of 50.6 and 51.8, respectively). Conversely, the one-electron reduced radical anions are not reactive toward carboxylation (ΔG^0^ > +20 Kcalmol^-1^ for both substrates, in either α or β position, consistent with pK_a_ of the conjugate acids < 18.5). For all the possible intermediates, the plot of calculated ΔG^0^ of carboxylation vs. pK_a_ is consistent with the linear correlation model developed. The application of the ΔG^0^ vs. pK_a_ correlation is finally discussed for alternative reaction mechanisms and for carboxylation of other C=C and C=O double bonds. These results offer a new mechanistic tool for the interpretation of the reactivity of CO_2_ with organic intermediates.

## Introduction

The activation and transformation of small molecules are pillars of artificial photosynthesis. In particular, carbon dioxide is an appealing target substrate because it is the product of combustion of organic compounds, and its levels in the atmosphere are continuously rising due to anthropogenic emissions while contributing to the greenhouse effect and global warming. Activation of CO_2_ can be accomplished through reduction routes (Francke et al., 2018; Melchionna et al., 2021) in which desirable products are carbon monoxide, formic acid, methanol, methane, or > C2 species (Albero et al., 2020). Alternatively, carbon dioxide can be exploited in cyclic carbonates or heterocycle formation (North et al., 2010; Fiorani et al., 2015; Yu and He, 2015; Guo et al., 2021; Vieira et al., 2018, 2019; Faria et al., 2021) or as a single-carbon-atom building block for its fixation into organic compounds (Liu et al., 2015; Cao et al., 2018; Cherubini-Celli et al., 2018; Tlili and Lakhdar, 2020; Zhang et al., 2020; Sahoo et al., 2021; Yuan et al., 2021; He et al., 2020) upon creation of new C-C or C-heteroatom bonds. Mechanistically, these processes can be accomplished through 1) the reduction of carbon dioxide to its radical anion, followed by its reaction with the organic scaffold (in dimethylformamide, E^0^(CO_2_/CO_2_^•–^) = −2.21 V vs. saturated calomel electrode, SCE, corresponding to −1.97 V vs. standard hydrogen electrode) (Lamy et al., 1977; Otero et al., 2006; Berto et al., 2015) or 2) upon the formation of reduced intermediates of the organic substrate accomplished through chemical, electrochemical, or photochemical routes and their subsequent reactivity with CO_2_ (Yuan et al., 2021).

This second possibility includes reductive activation of C-LG bonds (LG^–^ is a leaving group, often a halide ion) (Meng et al., 2017; Isse et al., 1998; Isse et al., 2002; Isse and Gennaro, 2002; Scialdone et al., 2008; Durante et al., 2013) of C=C or C=N double bonds (Seo et al., 2017; Fan et al., 2018; Chen et al., 2020; Fan et al., 2018; Schmalzbauer et al., 2020) and of C-H bonds (Gui et al., 2017; Seo et al., 2017; He et al., 2020). Recent examples include activation of substituted olefins (Alkayal et al., 2020), of diverse carbonyl compounds (Okumura and Uozumi, 2021) including α-ketoamides and α-ketoesters (Cao et al., 2021), α,β-unsaturated esters (Sheta et al., 2021) and ketones (Chen et al., 2020), and of aldimines generated *in situ* for α-aminoacid synthesis (Naito et al., 2021).

In all cases, a carbanion (hereafter generally indicated as C^−^) is postulated to be the key intermediate that reacts with CO_2_ although the nature of the reactive species and the mechanistic comprehension of the reactivity often remain elusive.

As reported by Mayr and coworkers (Li et al., 2020), the prediction of the reactivity of carbon-based nucleophiles with CO_2_ is problematic using linear-free energy relationships based on nucleophilicity and electrophilicity parameters (Li et al., 2020; Orlandi et al., 2021); the failure to observe carboxylation products with a variety of nucleophilic carbanions may be caused by unfavorable thermodynamics of the reaction (Li et al., 2020).

Therefore, we aimed at developing a general tool to predict the thermodynamics of a carboxylation reaction involving a carbon-based anion C^−^ by exploiting the basicity of C^−^ as a thermodynamic parameter. We propose a thermodynamic analysis supported by density functional theory calculations on 50 small organic molecules that enable the to correlate the standard free energy of the carboxylation reaction with the basicity of the carbanion C^−^, expressed in terms of the pK_a_ of the C-H/C^−^couple. We then apply the model to a real case involving electrochemical carboxylation of α,β-unsaturated carbonyls as the selected model substrates, and finally discuss alternative reaction mechanisms for the carboxylation of C=C and C=O double bonds.

## Results and Discussion

### Thermodynamic and DFT Analysis of Carbanions Reactivity with CO_2_

We employed a thermodynamic analysis to correlate the standard free energy of carboxylation of C^−^ ( $\Delta G_{1}^{0}$ in Eq. 1) with the basicity of the carbanion (expressed on the basis of the pK_a_ of the conjugate acid C-H, Eq. 2) as a thermodynamic descriptor of its reactivity. This analysis was inspired by a similar one reported by Kubiak and coworkers for correlating the hydricity of metal hydrides with the redox potential of the metal center and for evaluating the standard free energy for the reaction of the metal hydride with CO_2_ to produce formate (Waldie et al., 2018).

$$C^{-}\;+\;CO_{2\;}⇌C-CO_{2}^{-}\;\;\;\;\;\;\Delta G_{1}^{0}$$ (1)

$$\;\;C-H⇌C^{-}+H^{+}\;\;\;\;\;\;\;\Delta G_{2}^{0}=+2.303RT\cdot pK_{a}\left(CH,C^{-}\right)$$ (2)

We then considered Eqs. 3, 4, for which the ΔG^0^ in acetonitrile is reported (Waldie et al., 2018), with the goal of expressing the $\Delta G_{1}^{0}$ as a function of the pK_a_ of the C-H/C- couple.

$$H-CO_{2}^{-}⇌\;H^{-}\;+\;CO_{2\;}\;\;\;\;\;\;\Delta G_{3}^{0}=+44\;Kcalmol^{-1}\;in\;CH_{3}CN$$ (3)

$$H^{-}\;+\;H^{+}⇌H_{2}\;\;\;\;\;\;\;\Delta G_{4}^{0}=\;-76\;Kcalmol^{-1}\;in\;CH_{3}CN$$ (4)

Eq. 5 derives from the sum of Eqs. 1–4:

$$C-H+H-CO_{2}^{-}⇌C-CO_{2}^{-}\;+\;H_{2}\;\;\;\;\;\Delta G_{5}^{0}$$ (5)

$\Delta G_{5}^{0}$ (in kcalmol^−1^), thus, results in

$$\Delta G_{5}^{0}\;\left(Kcalmol^{-1}\right)=\Delta G_{1}^{0}+2.303RT\cdot pK_{a}\left(CH,C^{-}\right)+\;44-76$$ (6)

and, rearranging,

$$\left\{\Delta G_{1}^{0}-\;\Delta G_{5}^{0}\right\}\;\left(Kcal\;mol^{-1}\right)=+32-2.303RT\cdot pK_{a}\left(CH,C^{-}\right)$$ (7)

Eq. 7, thus, predicts that the difference between $\Delta G_{1}^{0}$ and $\Delta G_{5}^{0}$ depends linearly on the pK_a_ of the C-H/C^−^ couple.

**CHART 1:**
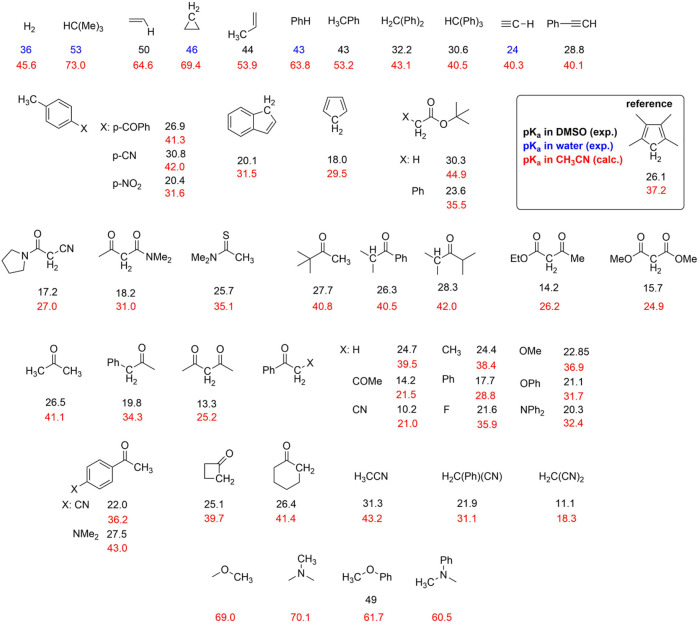
Organic molecules considered in the calculations with experimental and computed pK_a_ values. The experimental pK_a_ values are reported from the Reich database https://organicchemistrydata.org/hansreich/resources/pka/#pka_dmso_compilation (black and blue values refer to DMSO and water solvent, respectively). Computed pK_a_ values (red) were evaluated by DFT calculations using the relative determination method (Ding et al., 2009), by selecting 1,2,3,4-tetramethylcyclopentadiene as the reference (pK_a_ = 37.2).

In order to evaluate the separate dependence of $\Delta G_{1}^{0}$ and $\Delta G_{5}^{0}\;$ on the pK_a_ predicted by Eq. 7, we performed DFT calculations on 50 organic molecules containing C-H groups spanning different acidity with experimental pK_a_ in the range 9 ÷ 53 reported mainly in dimethylsulfoxide (DMSO), see Chart 1 (experimental pK_a_ values are reported from the Reich database: https://organicchemistrydata.org/hansreich/resources/pka/#pka_dmso_compilation). The choice of these 50 molecules was based on the simplicity of the organic scaffold, on the availability of the experimental pK_a_, and on the possibility of spanning a sufficiently large range of acidity. For these molecules, we calculated the pK_a_ of the C-H groups (Eq. 2), the ΔG^0^ of carboxylation of the anion C^−^ (Eq. 1), and the ΔG^0^ referred to Eq. 5, employing a geometry optimization at a b3lyp/6–311g (d,p) level with frequency analysis (Mateos et al., 2020), and including a continuum model for the acetonitrile solvent. Acetonitrile was considered because it provides a high solubility of CO_2_ of 0.28 M (Azcarate et al., 2016) and, thus, is widely used in carboxylation reactions. The calculations were done on the parent neutral molecules, on the corresponding carbanions, and on the carboxylated products, i.e., on 150 species.

Several experimental pK_a_ values of C-H moieties are reported in DMSO; however, pK_a_ is solvent-dependent (Daasbjerg, 1995; Workentin et al., 1995; Izutzu, 1990), and in acetonitrile, it can be rescaled according to pK_a_ (CH_3_CN) = 11.6 + 0.98⋅pK_a_ (DMSO); (Ding et al., 2009; Roszak et al., 2019).

When experimental values are not available (as in the case of some intermediates discussed in this work, *vide infra*), pK_a_ can be predicted computationally. Thus, the pK_a_ values of the C-H groups of the 50 species in Chart 1 were calculated by DFT, using the *relative* determination method (Ding et al., 2009; Kadiyala et al., 2013; Fu et al., 2005) by employing 1,2,3,4-tetramethylcyclopentadiene reference (pK_a_ value in CH_3_CN of 37.2 derived from an experimental pK_a_ = 26.1 in DMSO).

**FIGURE 1:**
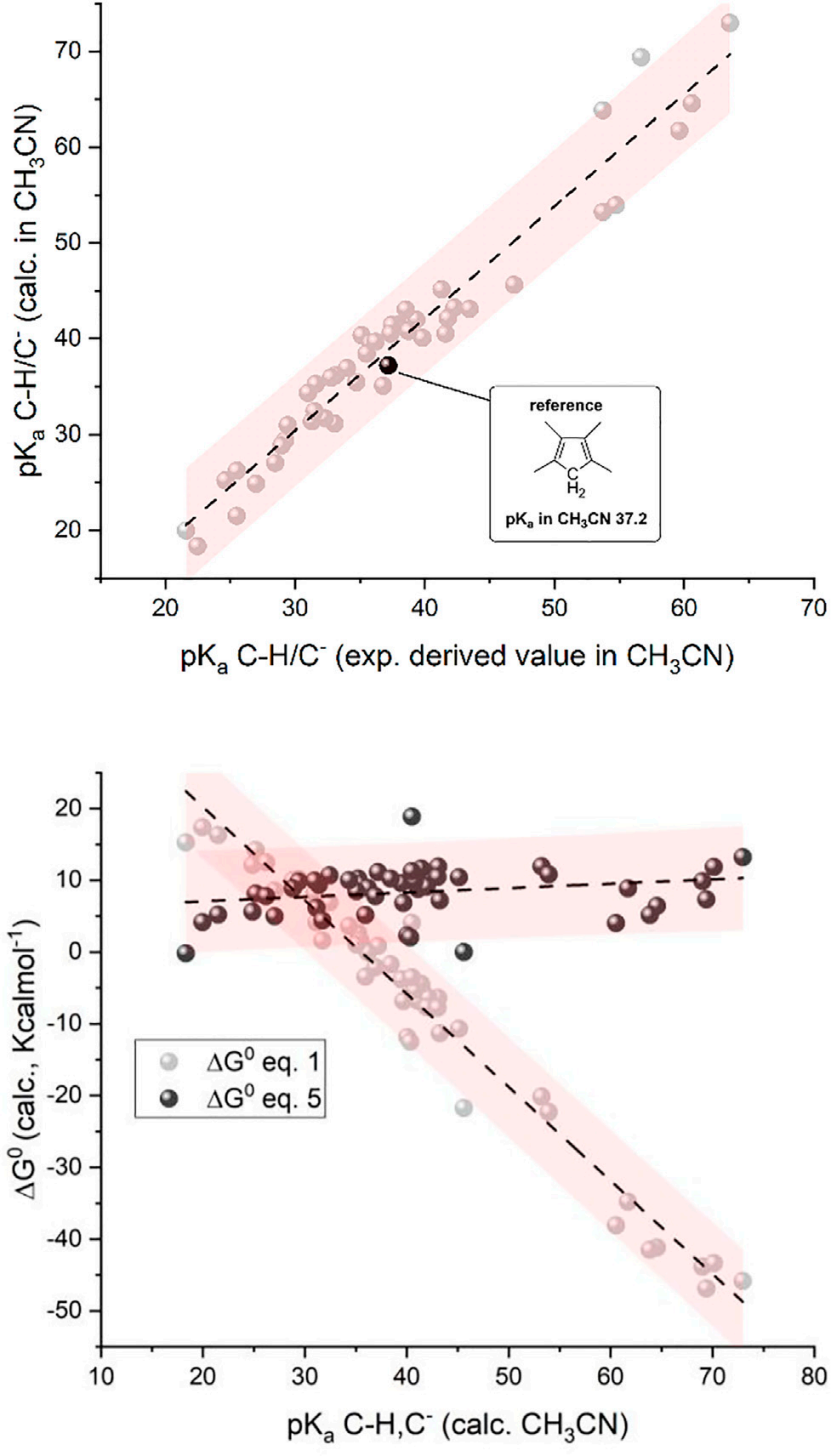
Top: Plot of calculated vs. experimental pK_a_ for the 50 organic substances considered; see Chart 1 (CH/C^−^ couples; 1,2,3,4-tetramethylcyclopentadiene as the reference; see the black dot with pK_a_ = 37.2 in CH_3_CN). Bottom: Plot of calculated standard free energy of carboxylation ( $\Delta G_{1}^{0}$ , light gray dots) and standard free energy for Eq. 5 ( $\Delta G_{5}^{0}$ , dark gray dots) vs. calculated pK_a_ of the C-H/C^−^ couples. In the calculations, $\Delta G_{1}^{0}$ of carboxylation (Eq. 1) was considered as the free energy of the C-CO_2_^−^ species, subtracting the free energy of C^−^ and of CO_2_; the calculation on the CO_2_ molecule still considered the continuous model of acetonitrile solvent. The pink shaded areas represent the 95% confidence interval of the correlations.

As shown in the top panel of Figure 1, the plot of calculated vs. experimental (derived values in CH_3_CN) pK_a_ values show a linear correlation with a slope of 1.17 ± 0.04, an intercept of -4.7 ± 1.6, and an R-square of 0.95; except for one case, all the points stand within the 95% confidence interval of the linear correlation; the major deviations are observed for species with experimental pK_a_ values > 35, for which the available data are more limited and subject to uncertainties.

We then determined the ΔG^0^ of reactions 1 and 5 (by the difference between the calculated free energy of products and reactants) for the 50 organic substrates and plotted the calculated $\Delta G_{1}^{0}$ and $\Delta G_{5}^{0}$ values versus the calculated pK_a_ of the C-H/C^−^couples (Figure 1, bottom panel). Interestingly, the plot of $\Delta G_{1}^{0}$ shows a marked dependence on pK_a_ (light gray dots in Figure 1, bottom) observing a trend fitted with a linear equation having a slope of −1.30 ± 0.04 Kcalmol^-1^ and an R-square of 0.96; the slope is close to the value of −2.303RT = −1.36 Kcalmol^-1^ at 298 K (Wiedner et al., 2016) and, thus, indicates that the major dependence of the ( $\Delta G_{1}^{0}-\;\Delta G_{5}^{0}$ ) left term in Eq. 7 on the pK_a_ is associated with the contribution of $\Delta G_{1}^{0}$ . Consistently, the plot of $\Delta G_{5}^{0}$ shows a weak pK_a_ dependence with a slope of 0.06 ± 0.03 Kcalmol^-1^ resulting from the linear fitting (dark gray dots in Figure 1, bottom panel); this weak dependence can be explained by considering that $\Delta G_{5}^{0}$ can be approximated as in Eq. 8 (i.e., by considering negligible entropic effects and expressing the $\Delta G_{5}^{0}$ as the difference/sum of bond enthalpies *DH* of the bonds being formed/broken in Eq. 5):

$$\Delta G_{5}^{0}\;\approx \;\;-DH_{H-H\;}-\;DH_{C-C\left(OO\right)}+\;DH_{H-C\left(OO\right)}+\;DH_{C-H}$$ (8)

considering $DH_{H-H\;}\;$ = +104 kcalmol^‐1^ and $DH_{H-C\left(OO\right)}$ = +96 kcalmol^‐1^, (Blanksby and Ellison, 2003), the weak dependence of $\Delta G_{5}^{0}$ on pK_a_ is, thus, associated with the variation of *DΗ* of C-C(OO) and C-H bonds (Eq. 5).

In short, the presented analysis supports a linear correlation of the standard free energy of carboxylation of C^−^ ( $\Delta G_{1}^{0}$ in Eq. 1) with the pK_a_ of the C-H/C^−^ couples (Eq. 2) with a slope close to the theoretical value of −2.303RT = −1.36 Kcalmol^-1^ at 298 K; interestingly, the calculations predict a threshold pK_a_ value of ca 36 (in CH_3_CN, corresponding to ca 25 in DMSO) for the C-H/C^−^ couple that delimits positive/negative values of $\Delta G_{1}^{0}$ of the carboxylation reaction.

Clearly, the $\Delta G_{1}^{0}$ is associated with the equilibrium constant of Eq. 1, implying that, under suitable conditions (high CO_2_ concentration or pressure), the carboxylation of the carbanion can be observed also in the case of a slightly positive $\Delta G_{1}^{0}$ . Indeed, carboxylation of indene and phenylacetonitrile (calc. pK_a_ 31.5 and 31.1, respectively; calc. $\Delta G_{1}^{0}$ +7 and +4 Kcalmol^-1^, respectively) was observed in DMSO in the presence of carbon dioxide, 18-crown-6 and K_2_CO_3_ as a base (Chiba et al., 1992; Chiba et al., 1994). The occurrence of an equilibrium in Eq. 1 is also associated with microscopic reversibility, by which backward decarboxylation can occur (Destro et al., 2020; Kong et al., 2020; Zhou et al., 2021): carboxylation and decarboxylation processes are typically associated with a low energy barrier in aprotic solvents (Zhou et al., 2021).

### Evaluation of the Model for Electrochemical Carboxylation of α,β-Unsaturated Carbonyls

We then examined the consistency of the predictive model with the experimental electrochemical carboxylation of flavone and chalcone as representatives of α,β-unsaturated carbonyl scaffolds retaining significant biological interest (Zhuang et al., 2017; Pietta, 2000). Moreover, under cathodic conditions, these substrates lead to the formation of multiple reduced intermediates, thus providing an ideal platform to assess their reactivity with carbon dioxide: the electrochemical methodology is indeed suitable to selectively generate the desired intermediate by tuning the operating potential.

#### Carboxylation of Flavone

**FIGURE 2:**
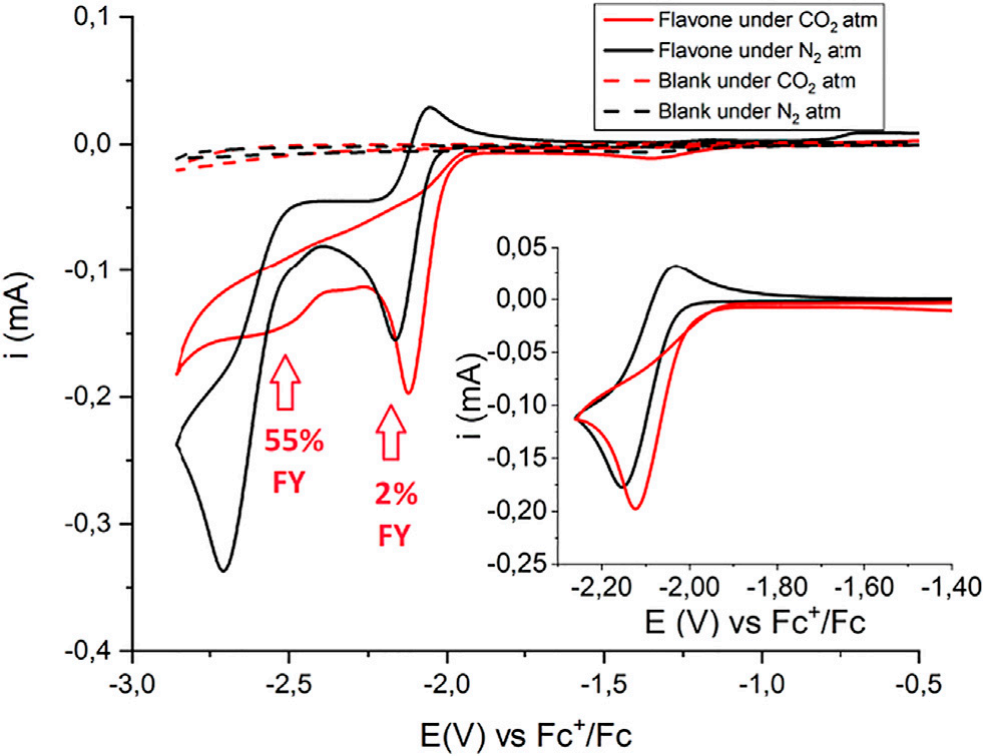
CV of 5 mM Flavone in CH_3_CN with 0.1 M tetrabutylammonium hexafluorophosphate supporting electrolyte under N_2_ (black traces) and CO_2_ (red traces). The inset shows the scan conducted in a narrow potential range and limited to the first reduction process. Glassy carbon working electrode (GC, 0.3 cm diameter, 0.07 cm^2^ geometric area), Pt counter electrode, Ag/AgCl reference electrode, scan rate 0.1 V s^−1^. Potentials were then converted to Fc^+^/Fc registering a CV scan of a ferrocene solution under the same conditions. The red arrows and the corresponding FY values refer to the carboxylation process and to the production of flavanone-2-carboxylic acid methyl ester. In principle, the operating potentials could be compatible with the production of the carbon dioxide radical anion, CO_2_^•–^: the E^0^(CO_2_/CO_2_^•–^) = −2.21 V vs SCE corresponding to −2.63 V vs. Fc^+^/Fc (Lamy et al., 1977; Berto et al., 2015; Christensen et al., 1990). However, only a slight increase of the CV traces below −2.7 V is observed passing from N_2_ to CO_2_ in the control experiments in the absence of flavone (dashed black and red traces, respectively). With GC electrodes, reduction of CO_2_ suffers indeed of an additional overpotential, and gives CO with almost quantitative Faradaic yield, accompanied by formation of CO_3_^2-^ (Berto et al., 2015; Christensen et al., 1990). Considering the ca 15-fold larger current observed in the presence of flavone (5 mM) and the higher concentrations of flavone used in CPE experiments (20 mM), the reduction of flavone is envisaged as the predominant route under these conditions.

Cyclic voltammetry of flavone **F** under cathodic scan shows a first, quasi-reversible wave at E_1/2_ = −2.09 V vs Fc^+^/Fc (ΔE = 120 mV) due to the one-electron reduction of **F** to the flavone radical anion, **F(RA)** (see Figure 2); scanning the CV analysis toward more negative potentials, a second, irreversible wave is observed peaking at E = −2.71 V vs Fc^+^/Fc, associated with the formation of the dianion **F(DA)** and its further reduction (see Figure 2); previous polarographic evidence suggests the occurrence of a two-electron process for this second wave due to a further irreversible reduction of **F(DA)** at this potential (Vakulskaya et al., 2011). Under CO_2_ saturation, the first wave becomes completely irreversible, and the cathodic peak shifts toward less negative potentials by 50 mV (Figure 2). A major change is instead associated with the second wave, suggesting reactivity of **F(DA)** with CO_2_; the decrease of the current suggests that the presence of CO_2_ inhibits the further reduction of the **F(DA)** with the latter likely involved in a different reaction pathway with CO_2_.

**SCHEME 1:**
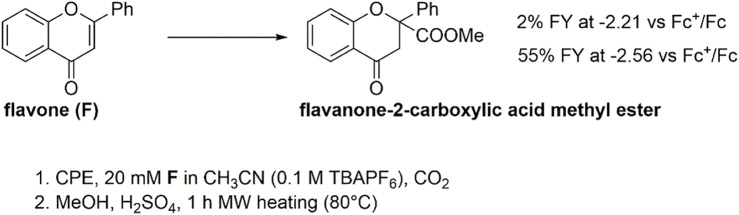
Electrochemical carboxylation of flavone **F** and formation of flavanone-2-carboxylic acid methyl ester (from carboxylation in β-position to the carbonyl group) after esterification of the electrolysis solution.

Controlled potential electrolysis (CPE) experiments were then performed to assess the reactivity of both the **F(RA)** and **F(DA)** species by applying a suitable operating potential to a glassy carbon rod working electrode. Electrolysis was conducted in a cell with two compartments separated by a ceramic frit. To evaluate the Faradaic yield of formation of the carboxylation product(s), an esterification procedure was performed involving treatment of the electrolyzed solution with H_2_SO_4_ in methanol for 1 h under microwave heating at 80°C (Scheme 1).

**Reactivity of flavone radical anion, F(RA)**: In the presence of CO_2_, a CPE held at −2.21 V (a potential associated with the electrogeneration of **F(RA)**), led to the production of flavanone-2-carboxylic acid methyl ester in a 2% Faradaic yield (Scheme 1, see also the red arrow in Figure 2; in CPE, a 20 mM concentration of flavone was used, fourfold higher with respect to CV conditions), and the electrolysis led mainly to the formation of 2,2-biflavanone (racemate and meso forms) dimerization by-products (see Supporting Information) (Sisa et al., 2010). This result suggests that **F(RA)** is not an intermediate favorably reacting with CO_2_ along a carboxylation reaction.

The unfavorable reactivity of **F(RA)** with CO_2_ is supported by DFT analysis. **F(RA)** was optimized as a doublet, displaying spin density mainly at the carbon in β to the carbonyl group (0.28 spin density) at the ortho and para positions of the phenyl ring in β (0.16–0.22 spin density) and at the carbonyl group (0.12 and 0.13 spin density at the carbon and oxygen atoms, respectively); no significant spin density is observed at the carbon in α to the carbonyl (see Supplementary Figure S1).

**SCHEME 2:**
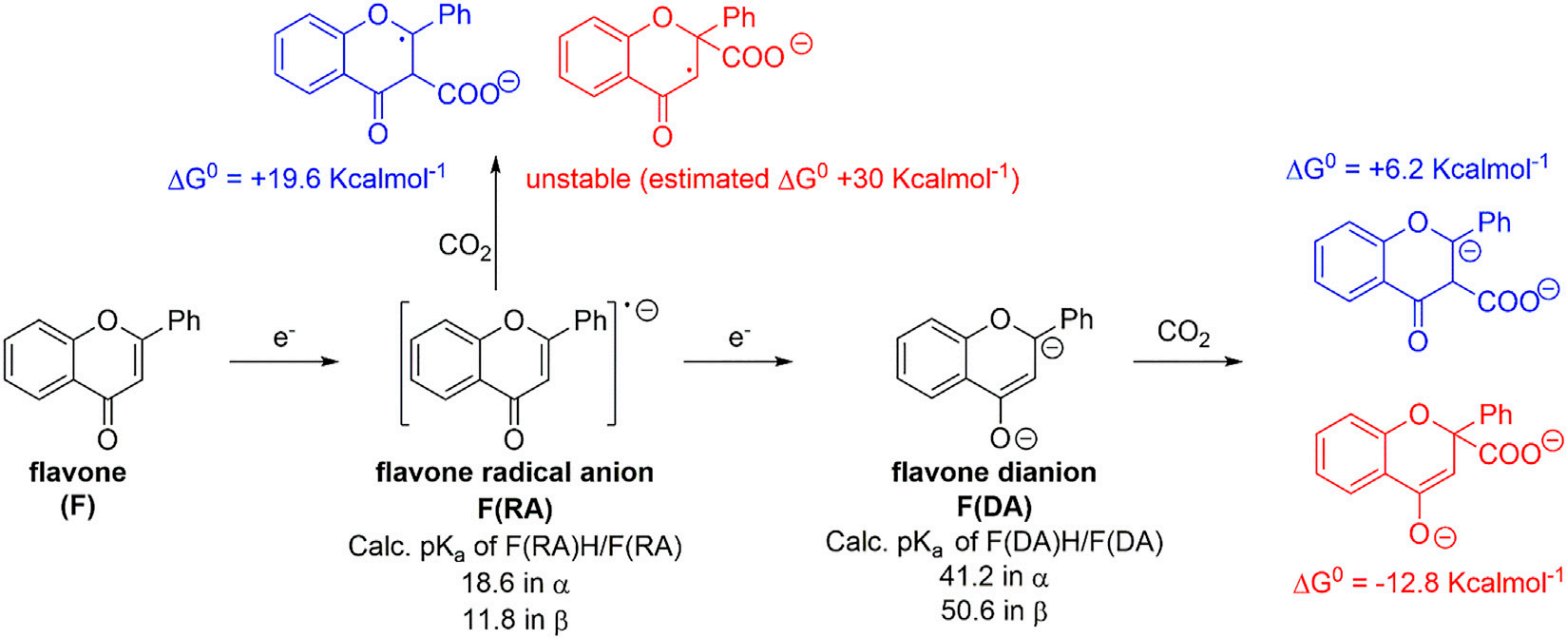
Generation of radical anion **F(RA)** and dianion **F(DA)** of flavone **F**, calculated pK_a_ values of their conjugate acids, and their predicted reactivity with CO_2_ in terms of calculated ΔG^0^ values. Blue and red structures refer to carboxylation in α and β positions, respectively.

**FIGURE 3:**
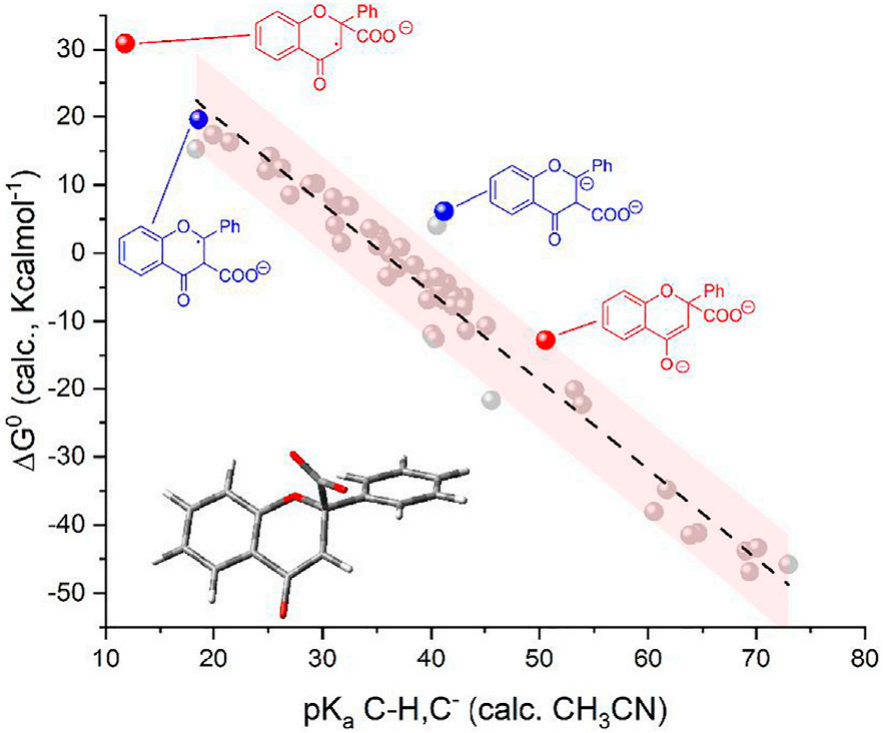
Plot of calculated standard free energy of carboxylation ( $\Delta G_{1}^{0}$ ) vs. calculated pK_a_ of the CH/C^−^ couples. Red dots indicate flavone derivatives upon carboxylation in the β position; blue dots indicate flavone derivatives upon carboxylation in the α position. The carboxylation product in β starting from F(RA) is unstable during the calculation and explodes releasing CO_2_: in this case, the ΔG^0^ value is extrapolated by the linear correlations among the 50 organic substrates. Inset: optimized geometry of the carboxylated product in β starting from **F(DA)**. The light gray dots indicate the 50 organic molecules reported in Chart 1 and plotted in Figure 1.

Calculations on the conjugate acids of **F(RA)** were performed by considering protonation of **F(RA)** in α or β positions; the computed free energies allowed to determine the calculated pK_a_ of the C-H/C^−^ couples according to the abovementioned procedure, resulting in pK_a_ values of 18.6 and 11.8 for the α and β positions, respectively. Both these values fall above the previously discussed threshold to reach a favorable carboxylation process. Consistently, a calculated ΔG^0^ of +19.6 kcalmol^−1^ was found for the carboxylation of **F(RA)** in α position, and the carboxylation product of **F(RA)** in the β position was unstable during the optimization process, decomposing into CO_2_ and **F(RA)**; see Scheme 2 and Figure 3.

**Reactivity of flavone dianion, F(DA)**: CPE at -2.56 V vs Fc^+^/Fc (Scheme 1, see also the red arrow in Figure 2) leads to the β-C carboxylation of flavone with 55% Faradaic yield upon isolation of the flavanone-2-carboxylic acid methyl ester. This evidence indicates a reaction of **F(DA)** with CO_2_ in the β-position (Senboku et al., 2011; Senboku et al., 2012). The reactivity of **F(DA)** with CO_2_ was supported by DFT calculations. **F(DA)** was optimized as a singlet state (Supplementary Figure S2), and similarly to the case of **F(RA)** previously discussed, calculations on the conjugate acids of the **F(DA)** were performed by considering protonation of **F(DA)** in the α and β positions to determine the calculated pK_a_ of the C-H/C^−^ couples: pK_a_ values of 41.2 and 50.6 were obtained for the conjugate acids of **F(DA)** in the α and β positions, respectively (Scheme 2 and Figure 3). Concerning the carboxylation upon reaction of **F(DA)** with CO_2_, calculated ΔG^0^ of +6.2 kcalmol^−1^ and of −12.8 Kcalmol^-1^ were found for the carboxylation of **F(DA)** in the α and β positions, respectively (Scheme 2 and Figure 3), thus supporting the preferred carboxylation in β-position (Senboku et al., 2011; Senboku et al., 2012).

Importantly, the calculated ΔG^0^ vs pK_a_ values for **F(RA)** (in the α position) and **F(DA)** (in both α and β positions) were observed to be consistent with the trend predicted in Figure 1 right for the 50 organic substrates (Scheme 2 and Figure 3).

#### Carboxylation of *Trans*-Chalcone

**SCHEME 3:**
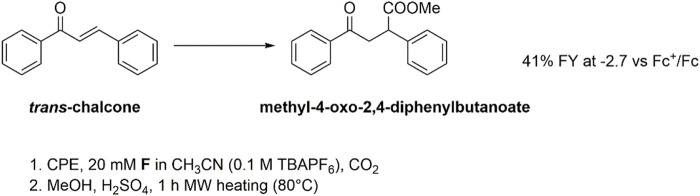
Electrochemical carboxylation of *trans*-chalcone **C** and formation of ethyl-4-oxo-2,4-diphenylbutanoate after esterification in methanol of the electrolysis solution. The Faradaic yield results lower with respect to galvanostatic conditions performed in a single compartment cell and employing aluminum anodes (Chen et al., 2020); this can be due to a stabilizing effect of the carboxylate product by electrogenerated Al(III) ions.

An analogous scenario was reached exploring the carboxylation of *trans*-chalcone (**C**). The CV analysis under cathodic scan and N_2_ atmosphere shows the presence of a first irreversible wave peaking at −1.9 V vs Fc^+^/Fc, attributed to the formation of the chalcone radical anion **C(RA)**, Supplementary Figure S3. This is followed by a second wave, composed of two contributions at E_1/2_ = −2.3 V vs Fc^+^/Fc and E_1/2_ = −2.45 V vs Fc^+^/Fc, likely associated with the formation of the chalcone dianion **C(DA)** (Chen et al., 2020); the splitting of the wave into two contributions with ca halved intensity with respect to the first one could be ascribed to the rotation of the C-C bond in **C(RA)**, leading to two cis/trans isomeric forms that are further reduced to **C(DA)** at slightly different potentials. Upon addition of CO_2_, the first wave is almost unaffected, and the second one shows marked changes with the formation of a single irreversible wave peaking at E_pc_ = −2.5 V vs. Fc^+^/Fc and, thus, suggesting reactivity of **C(DA)** with CO_2_. This is confirmed by CPE experiments, that allowed the isolation of methyl-4-oxo-2,4-diphenylbutanoate with 41% FY upon electrolysis at −2.7 V vs Fc^+^/Fc followed by esterification of the carboxylate product, consistent with effective carboxylation in the β position to the carbonyl group (Scheme 3).

**SCHEME 4:**
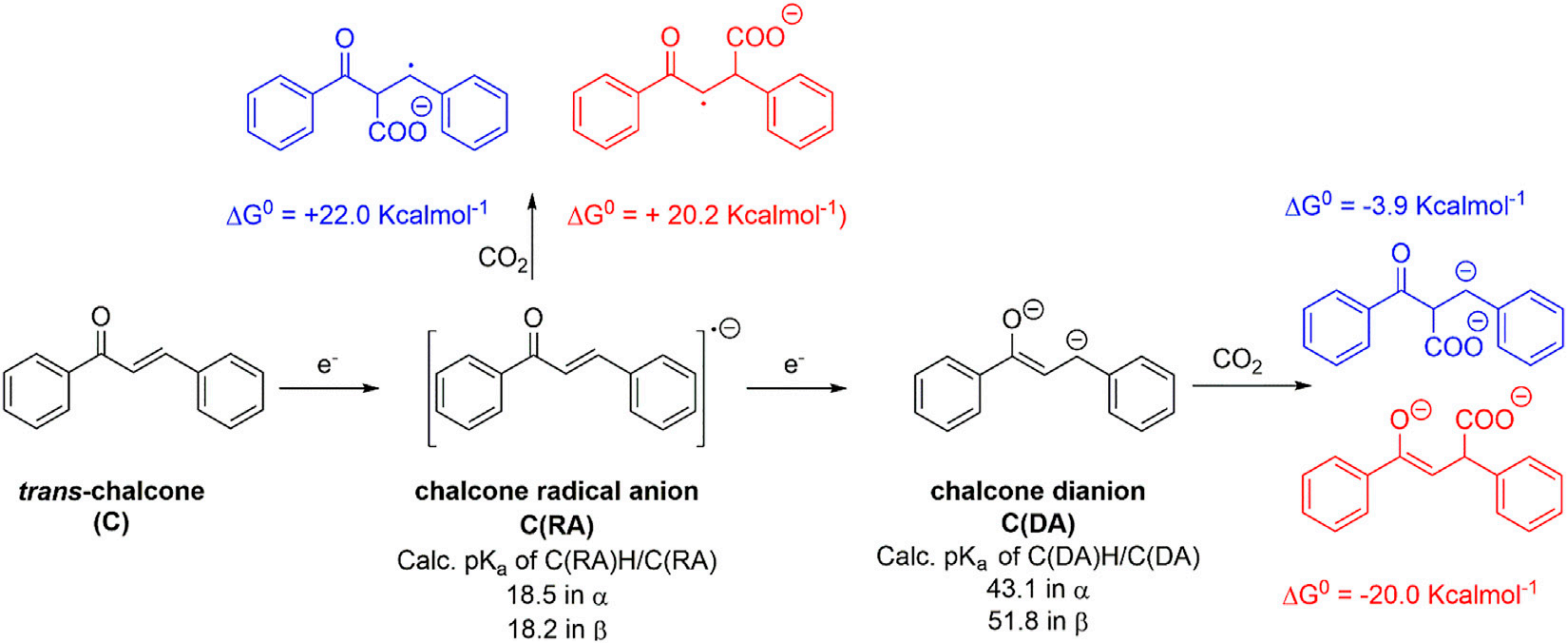
Generation of radical anion **C(RA)** and dianion **C(DA)** from *trans*-chalcone **C**, calculated pK_a_ values of their conjugate acids and their predicted reactivity with CO_2_ in terms of calculated ΔG^0^ values. Blue and red structures refer to carboxylation in α and β positions, respectively.

**FIGURE 4:**
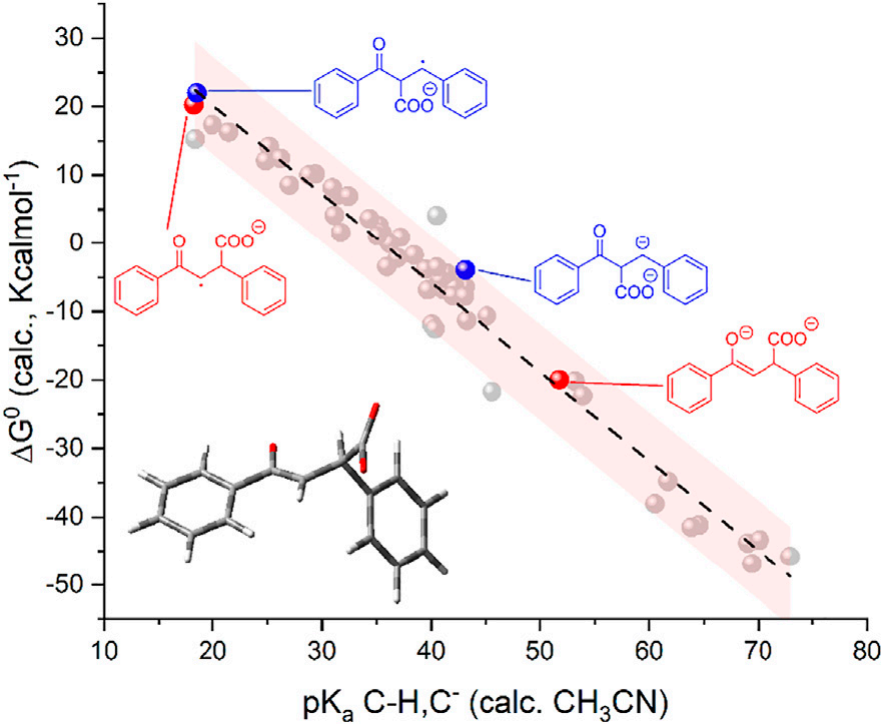
Plot of calculated standard free energy of carboxylation ( $\Delta G_{1}^{0}$ ) vs. calculated pK_a_ of the CH/C^−^ couples. Red dots indicate chalcone derivatives upon carboxylation in the β position; blue dots indicate chalcone derivatives upon carboxylation in the α position. The light gray dots indicate the 50 organic molecules reported in Chart 1 and plotted in Figure 1. Inset: optimized geometry of the carboxylated product in β starting from **C(DA)**.

Similar to what previously discussed in the case of flavone, the reactivity trend of chalcone reduced species **C(RA)** and **C(DA)** toward CO_2_ was supported by DFT calculations (Scheme 4 and Figure 4). **C(RA)**, optimized as a doublet, shows a planar structure and a spin density localized mainly on the carbon in β to the carbonyl (0.33 spin density) and on the carbonyl group (0.18 and 0.19 spin density for C and O, respectively); see Supplementary Figure S4. Calculations on the conjugate acids of **C(RA)** by considering a protonation in the α and β positions to the carbonyl, lead to the determination of pK_a_ values of 18.5 and 18.2 in the α and β positions, respectively; these pK_a_ values are below the predicted threshold of reactivity with CO_2_, and consistently, positive ΔG^0^ of +22.0 and of +20.2 kcalmol^−1^ were obtained for the carboxylation of **C(RA)** in the α and β positions, respectively, see Scheme 4 and Figure 4.

Optimization of **C(DA)** as a singlet led to a planar structure (Supplementary Figure S5); calculations on the conjugate acids of **C(DA)** by inserting a proton in the α and β positions, led to the determination of pK_a_ of 43.1 and 51.8 for C-H groups in the α and β positions, respectively. Consistently, negative ΔG^0^ for the carboxylation reaction involving **C(DA)** were determined of −3.9 and −20.0 Kcalmol^-1^ for the α and β positions, respectively (Scheme 4 and Figure 4).

Again, the calculated ΔG^0^ vs. pK_a_ values for **C(RA)** and **C(DA)** in both α and β positions were observed to be consistent with the linear trend predicted in Figure 1 for the 50 organic substrates (Figure 4) with all the points standing within the 95% confidence interval, supporting the need to generate the dianion of chalcone to achieve the carboxylation in the β-position as experimentally observed.

#### Alternative Mechanisms and Perspectives in the Carboxylation of C=C and C=O Bonds

Based on the above discussion, some considerations of general relevance can be finally addressed and focused in particular on 1) the reactivity of radical anions, generated from a one-electron reduction of the parent C=C bond; this type of intermediate is often envisaged in photochemical carboxylation processes (Nikolaitchik et al., 1996; Seo et al., 2017); 2) the reactivity of carbanions generated from an activation of C=O bonds, via an umpolung strategy (Juhl and Lee, 2018; Juhl et al., 2019; Cao et al., 2021).

**SCHEME 5:**
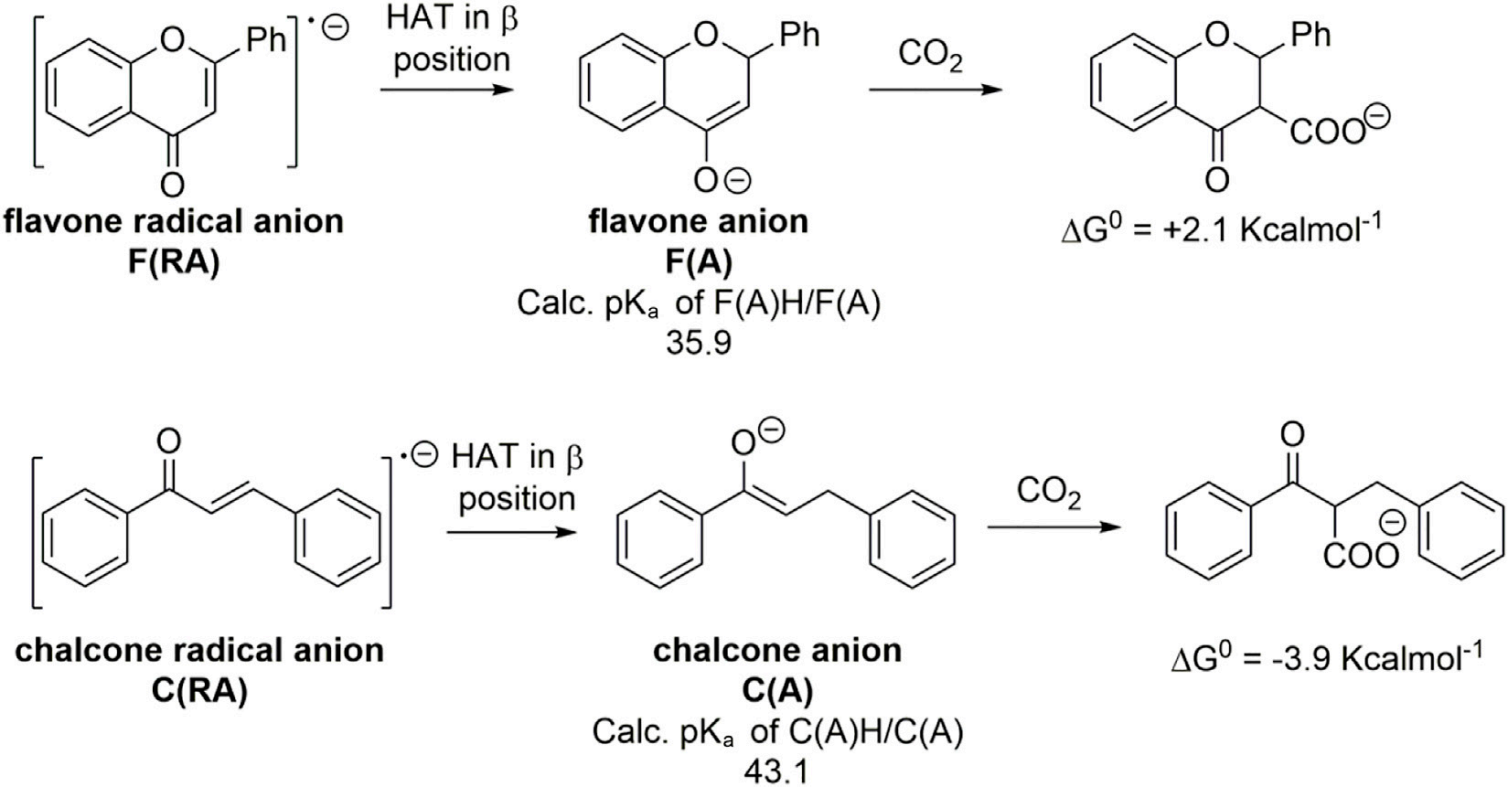
Generation of flavone and chalcone anions, **F(A)** and **C(A)** from a HAT to the flavone and chalcone radical anions **F(RA)** and **C(RA)**, calculated pK_a_ values of their conjugate acids, and their predicted reactivity with CO_2_ in terms of calculated ΔG^0^ values of the carboxylation reaction.

In the case of α,β-unsaturated carbonyl compounds, a positive, unfavorable ΔG^0^ in the reactivity of the radical anion toward CO_2_ seems to be a general feature as predicted by the DFT calculations on other α,β-unsaturated carbonyl scaffolds summarized in Supplementary Table S1. However, other reaction pathways that are alternative to the generation of a further reduced dianion intermediate can be envisaged to achieve carboxylation of this class of substrates. One possibility exploits hydrogen atom transfer (HAT) from a suitable HAT donor (Capaldo and Ravelli, 2017; Costas and Bietti, 2018). Still considering the representative case of flavone and chalcone, a HAT to **F(RA)** and **C(RA)** occurs preferentially in β positions to generate the corresponding flavone and chalcone anions, **F(A)** and **C(A)** (Scheme 5; these are 12.8 and 11.9 Kcalmol^-1^ more stable with respect to the isomeric species generated by a HAT in the α position to flavone and chalcone, respectively). Because the HAT is more favorable in the β position, the possible reactivity of **F(A)** and of **C(A)** with CO_2_ should occur in the α position.

**FIGURE 5:**
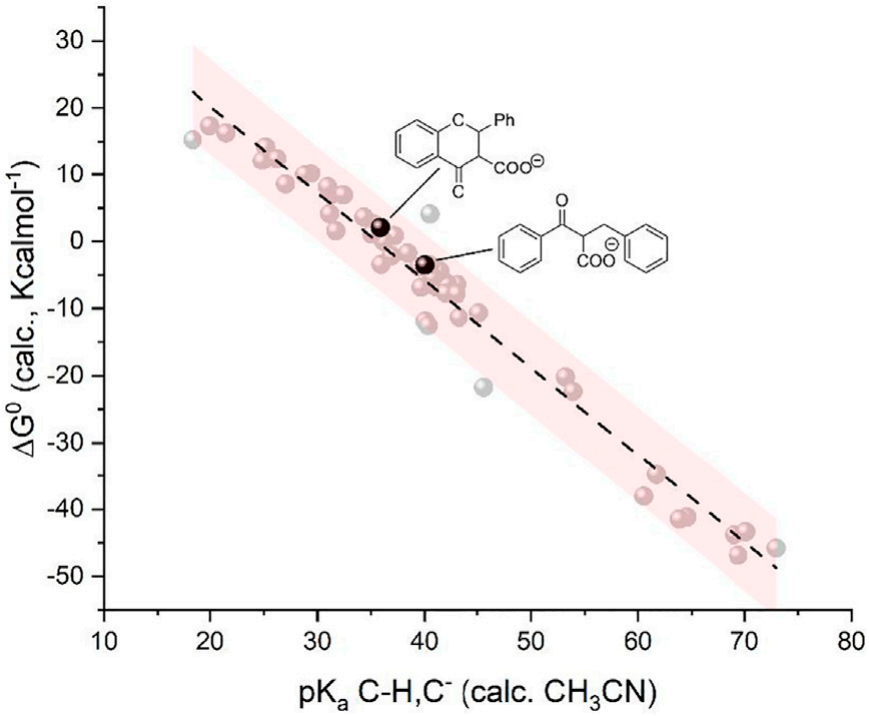
Plot of calculated standard free energy of carboxylation ( $\Delta G_{1}^{0}$ ) vs. calculated pK_a_ of the CH/C^−^ couples. The two black dots refer to the products upon carboxylation in α position to the carbonyl from flavone and chalcone anions **F(A)** and **C(A)**. The light gray dots indicate the same 50 organic molecules reported in Chart 1 and plotted in Figure 1.

Calculations predict pK_a_ values of 35.9 and 43.1 for the conjugate acids of **F(A)** and **C(A)**, respectively, and the ΔG^0^ for the carboxylation are +2.1 and -3.9 Kcalmol^-1^ starting from **F(A)** and **C(A)**, respectively (Scheme 5); the pK_a_ and ΔG^0^ values fit well with the model previously developed, standing within the 95% confidence interval (Figure 5). Therefore, the basicity of **F(A)** and **C(A)** is greatly enhanced with respect to the corresponding radical anions **F(RA)** and **C(RA)** with differences in the pK_a_ values of 17.3 and 24.6, respectively; the enhancement of basicity leads to a favorable gain in the ΔG^0^ of carboxylation in the α position of 17.5 and 25.9 Kcalmol^-1^ for flavone and chalcone, respectively, when passing from the radical anions to the anions. The use of a HAT donor additive can, thus, be considered in the carboxylation processes although the regioselectivity should be properly evaluated.

**FIGURE 6:**
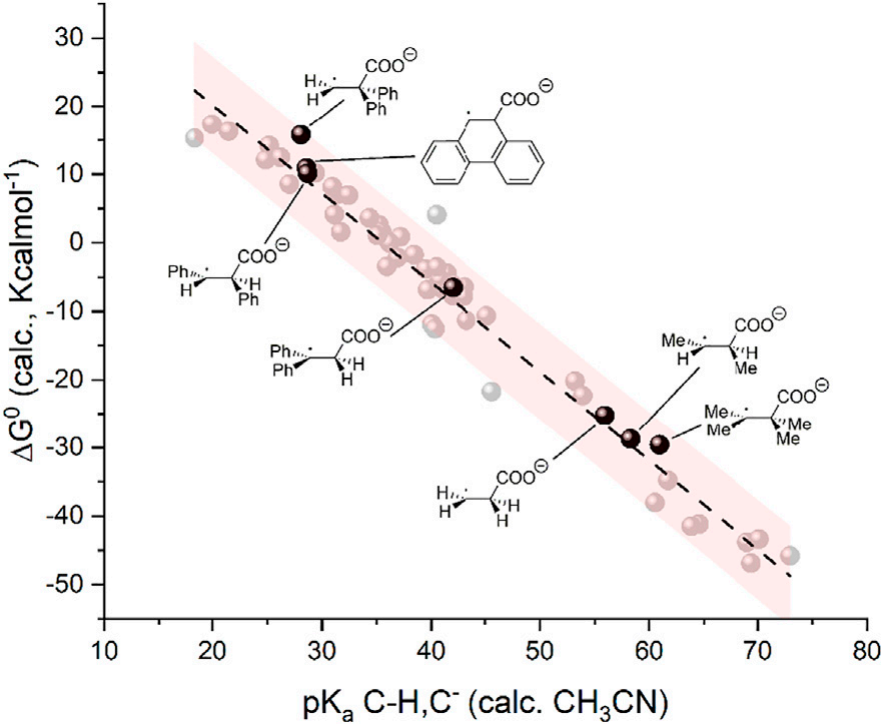
Plot of calculated standard free energy of carboxylation ( $\Delta G_{1}^{0}$ ) vs. calculated pK_a_ of the CH/C^−^ couples. The black dots refer to the products upon carboxylation from radical anions of alkenes. The light gray dots indicate the same 50 organic molecules reported in Chart 1 and plotted in Figure 1.

Although unfavorable in the case of α,β-unsaturated carbonyls, the negligible reactivity of radical anion intermediates toward carboxylation should not be considered as a general feature of C=C double bonds. We performed DFT calculations on the radical anions of selected alkenes, such as ethylene, 2-butene, and diphenylethylene isomers as well as of phenanthrene as representative of a fully aromatic scaffold to calculate the corresponding basicity and the ΔG^0^ of the carboxylation reaction involving these radical anions. The results are summarized in Supplementary Table S1 and plotted in the ΔG^0^ vs. pK_a_ graph of Figure 6. A nice match is observed between these data and those of the 50 organic substrates previously employed in the construction of the linear trend. Interestingly, these calculations predict that 1) radical anions of alkenes can be sufficiently basic to achieve a favorable ΔG^0^ for carboxylation when hydrogen or alkyl groups are bound to the C=C double bond; 2) phenyl groups bound to the carbon atoms of the C=C bond reduce the basicity of the radical anion and tend to disfavor the carboxylation (differences of ca 30 pK_a_ units and of 40 Kcalmol^-1^ in ΔG^0^ of carboxylation are observed by comparing *trans*-2-butene and *trans*-1,2-diphenylethylene); 3) when phenyl groups are present, reactivity is expected to be favorable if one of the carbon atoms of the C=C bond does not bear phenyl substituents as in the case of 1,1-diphenylethylene; and 4) radical anions of C=C bonds in aromatic scaffolds show unfavorable basicity and carboxylation reactivity as in the case of phenanthrene. Further investigations on structure-reactivity analysis on this kind of substrate are ongoing.

**SCHEME 6:**
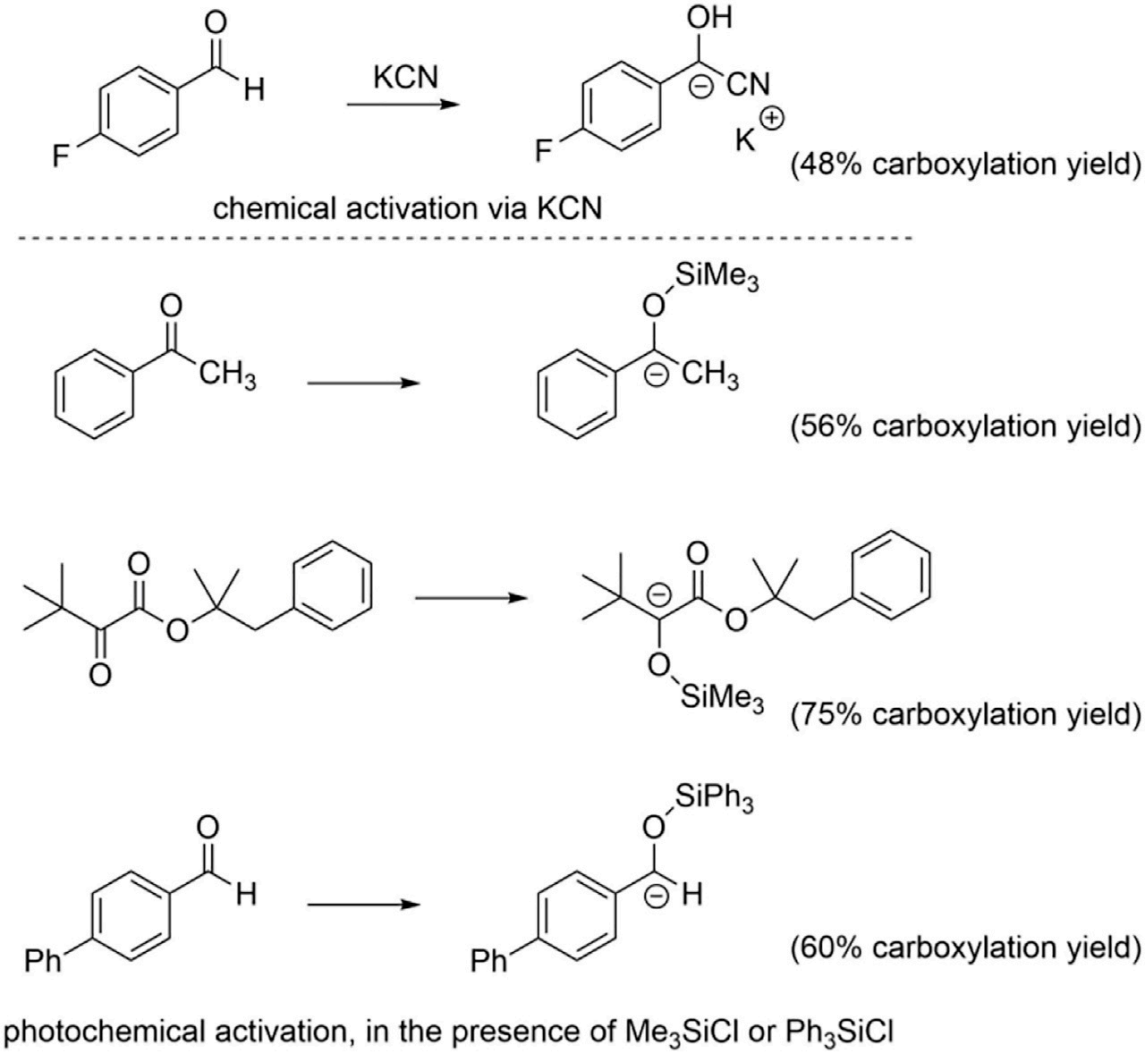
Formation of carbanions capable of carboxylation reactions via Umpolung activation of C=O bonds. See references for further experimental conditions (Juhl and Lee, 2018; Juhl et al., 2019; Cao et al., 2021).

**FIGURE 7:**
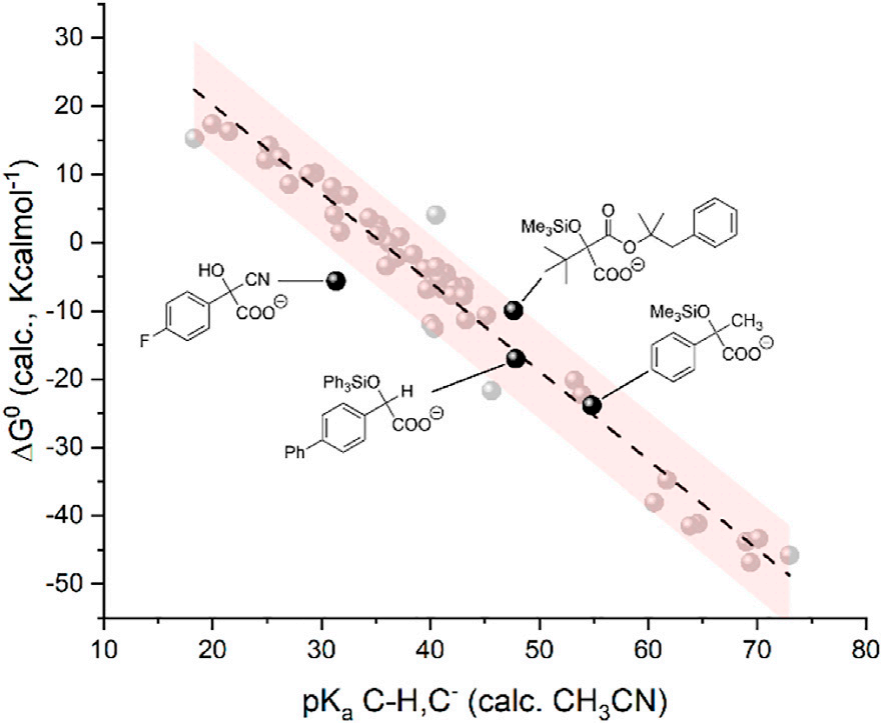
Plot of calculated standard free energy of carboxylation (ΔG^0^) vs. calculated pK_a_ of the CH/C^−^ couples. The black dots refer to the products upon carboxylation of anions generated from activation of C=O groups. The light gray dots indicate the same 50 organic molecules reported in Chart 1 and plotted in Figure 1.

We finally verified if the model is suitable for the prediction of carboxylation of carbanions generated by activation of C=O groups through an umpolung strategy. In particular, we considered a carbanion of 4-fluorobenzaldehyde activated via a cyanohydrin intermediate (Juhl and Lee, 2018) and the carbanions of alkyl aryl ketones, α-ketoesters, and aryl aldehydes generated through a photochemical process combining a trimethylsilyl (in the case of the alkyl aryl ketones and of α-ketoesters) or triphenylsilyl (in the case of aryl aldehydes) activating/protecting group, see Scheme 6 (Cao et al., 2021). Gratifyingly, the calculations predict a negative ΔG^0^ for the carboxylation of such intermediates (Juhl et al., 2019), thus supporting the experimental outcome (see the yields of carboxylation in Scheme 6) (Juhl and Lee, 2018; Cao et al., 2021). In addition, the pK_a_ and ΔG^0^ values fit well with the model previously developed (Figure 7).

## Conclusion and Perspectives

We present a thermodynamic analysis combined with density functional theory calculations that enable linearly correlating the standard free energy ΔG^0^ of the carboxylation reaction of a carbanion C^−^ with its basicity expressed as the pK_a_ of the CH/C^−^ couple. This offers a new mechanistic tool for the interpretation of the reactivity of CO_2_ with organic intermediates. The analysis identifies a threshold pK_a_ of ca 36 (in CH_3_CN) for the CH/C^−^ couple, above which the ΔG^0^ of the carboxylation reaction is negative and, thus, indicative of a thermodynamically favorable process. Because fast tools are nowadays available for the estimation of pK_a_ of C-H groups (Roszak et al., 2019), the pK_a_ vs ΔG^0^ correlation enables a fast analysis and prediction of the thermodynamics of the carboxylation reaction.

Application of the model to the electrochemical carboxylation of flavone and chalcone as representative compounds of α,β-unsaturated ketones allows the prediction of the carboxylation occurring in the β-position from the doubly reduced dianion intermediates of the starting compounds (ΔG^0^ of carboxylation in β = −12.8 and −20.0 Kcalmol^-1^ for flavone and chalcone, respectively, associated to pK_a_ values for the conjugate acid of 50.6 and 51.8, respectively). The one-electron reduced radical anions are instead not reactive toward carboxylation (ΔG^0^ > +20 Kcalmol^-1^ for both substrates in either α or β position, consistent with pK_a_ of the conjugate acid < 18.5). In all cases, the calculated pK_a_ and ΔG^0^ of carboxylation are consistent with the linear correlation model developed, thus supporting its application also to more complex organic scaffolds.

The analysis was extended to alternative carboxylation mechanisms and to other organic substrates that were already employed in carboxylation reactions in previous literature studies.

Further tuning of the model could consider possible specific stabilization of the species involved and, in particular, of the charged ones by the nature of the solvent or by the presence of additives. As discussed by (Pletcher and Slevin, 1996), Mg^2+^ ions are known to stabilize reduced intermediates and carboxylate species (Corbin et al., 2021) and are proposed to play a key role in the electrochemical carboxylation of benzalacetone (Mg^2+^ are typically generated under electrochemical conditions, when employing Mg sacrificial anodes) (Wang et al., 2017; Bhasha Sayyed and Sakaki, 2014).

## Experimental

The cyclic voltammetry (CV) characterizations were carried out with a three-electrode system controlled by a BASi EC Epsilon potentiostat-galvanostat. The working electrode was a glassy carbon disk electrode (BioLogic, nominal diameter 3 mm), the auxiliary electrode was a platinum electrode (BASi), and the reference electrode was an Ag/AgCl (NaCl 3 M) electrode; potentials were then referenced to the ferrocenium/ferrocene (Fc^+^/Fc) couple upon addition, at the end of each experiment session, of ferrocene to the analyte solutions as internal standard; 0.1 M tetrabutylammonium hexafluorophosphate (Bu_4_NPF_6_) was used as a supporting electrolyte.

Constant potential electrolysis experiments were performed with a Metrohm Autolab PGSTAT204 potentiostat-galvanostat in combination with the NOVA 2.1.4 software (https://www.metrohm-autolab.com/Products/Echem/Software/Nova.html).

The cell generally employed for preparative electrolysis was a custom-made, six-necked, two-compartment glass cell with the two compartments being separated by a porous glass frit.

Quantitative gas chromatographic (GC) analysis were performed on a Shimadzu GC-2010 Pro gas chromatograph equipped with a flame ionization detector (FID). Every measurement was performed by automatic injection of 1 μL of the sample solution. Quantification of the starting material and ester products was achieved by internal calibration of the instrument upon the construction of a calibration curve by the injection of known volumes of reagents and mesitylene as a standard. The response factor of the initial substrate was used also to quantify the ester product because the presence of one -COOCH_3_ additional group with respect to the initial substrate is expected to have a minor effect in the FID response.

^1^H NMR spectra were recorded on a Bruker 300 Advance spectrometer equipped with BBO probe head 5 mm. NMR spectra were processed using MestReNova software.

EI-MS spectra were registered using an Agilent 6,850 Network GC system equipped with a 5975 Series MSD detector. ESI-MS spectra were acquired with an Agilent Technology LC/MSD Trap SL, interfaced to an Agilent 1100 binary pump.

Esterification procedures were done by a CEM Discover microwave reactor (300 W maximum power) setting the bulk temperature at 80°C for 1 h.

For all species, geometry optimizations and frequency calculations were done to give the best suited Gibbs energies by DFT calculations performed at the b3lyp/6–311 + g(d,p) level of theory with Gaussian16 and GaussView 6 software packages (Frisch et al., 2016). The self-consistent reaction field was used with DFT energies, optimizations, and frequency calculations to model systems in acetonitrile solution. The convergence criteria for interatomic force minimization (geometry optimization) were the standard ones of the Gaussian16 software.

Further details are reported in Supplementary Information.

## Data Availability

The original contributions presented in the study are included in the article/Supplementary Material; further inquiries can be directed to the corresponding author.
