# Supplementary material for: Basicity as a Thermodynamic Descriptor of Carbanions Reactivity with Carbon Dioxide: Application to the Carboxylation of α,β-Unsaturated Ketones
Source: Front Chem. 2021 Nov 24;9:783993. doi: 10.3389/fchem.2021.783993 (PMC8652261; doi:10.3389/fchem.2021.783993)
Supplement: Supplementary file 1 [file DataSheet1.docx]

Supporting Information

Basicity as a thermodynamic descriptor of carbanions reactivity with carbon dioxide: application to the carboxylation of α,β-unsaturated ketones

Pietro Franceschi^1†^, Catia Nicoletti^1†^, Ruggero Bonetto^1^, Marcella Bonchio^1^, Mirco Natali^2^, Luca Dell’Amico^1^, Andrea Sartorel^1*^

^1^Nano and Molecular Catalysis Laboratory, Department of Chemical Sciences, University of Padova, via Marzolo 1, 35131 Padova, Italy.

^2^ Department of Chemical, Pharmaceutical and Agricultural Sciences (DOCPAS), University of Ferrara, and Centro Interuniversitario per la Conversione Chimica dell’Energia Solare (SOLARCHEM), sez. di Ferrara, via L. Borsari 46, 44121 Ferrara, Italy.

† P.F. and C.N. equally contributed.

*andrea.sartorel@unipd.it

**Table of Contents**

A. General Information 3

A.1. Electrochemical characterization procedures by cyclic voltammetry 4

A.2. Esterification procedure 5

A.3. GC analysis 6

A.3.1. Flavone calibration curve 6

A.3.2. Chalcone calibration curve 7

A.4. DFT calculations 8

B. General Procedures For Electrolysis 9

B.1. Procedures for Flavanone-2-carboxylic acid methyl ester synthesis 10

B.1.1 WE potential: -2.56 V vs Fc^+^/Fc 10

B.1.2 WE potential: -2.21 V vs Fc^+^/Fc 10

B.2. Procedure for Methyl 4-oxo-2,4-diphenylbutanoate synthesis 12

B.2.1 WE potential: -2.7 V vs Fc^+^/Fc 12

C. Supplementary Figures and Tables 13

D. Optimized Geometries 20

G. References 33

# A. General Information

NMR spectra were recorded on Bruker 300 Advance spectrometer equipped with BBO probe head 5 mm. The chemical shifts (δ) for ^1^H and ^13^C are given in ppm relative to residual signals of the solvents (CHCl_3_ @ 7.26 ppm ^1^H NMR, 77.16 ppm ^13^C NMR). Coupling constants are given in Hz. The following abbreviations are used to indicate the multiplicity: s, singlet; d, doublet; t, triplet; q, quartet; m, multiplet; bs, broad signal. NMR spectra were processed using MestReNova software.

EI-MS spectra were registered using an Agilent 6850 Network GC system equipped with a 5975 Series MSD detector. The analyses were performed using an HP-5MS column (30 m length, 0.25 mm, Ø 0.25 mm, 0.25 micron film thickness).

ESI-MS spectra were acquired with an Agilent Technology LC/MSD Trap SL, interfaced to an Agilent 1100 binary pump. All mass spectra were registered upon flow injection of samples diluted in acetonitrile, to reach a concentration of 10^-5^ M. The eluent composition was chosen as pure HPLC-grade acetonitrile with 0.1% formic acid.

Gas chromatographic (GC) analyses were performed on a Shimadzu GC-2010 Pro gas chromatograph equipped with a flame ionization detector (FID). The analyses were performed using an equity-5 column (15 m, Ø 0.1mm, 0.1 micron film thickness) and helium as a carrier gas. Every measurement was performed by automatic injection of 1 μL of the sample solution. Column oven temperature program: 70°C (hold for 2 min), then 25°C/s until 270 °C (hold for 9 min), total program time: 19 min.

The electrochemical characterizations were carried out on a BASi EC Epsilon potentiostat-galvanostat in a typical three-electrode cell.

Electrosynthesis experiments were performed with a Metrohm Autolab PGSTAT 2014 potentiostat-galvanostat in combination with the Nova 2.1.4 software (<https://www.metrohm-autolab.com/Products/Echem/Software/Nova.html>).

Esterification procedures were done by a CEM Discover microwave reactor (300 W maximum power) setting the bulk temperature at 80°C for 1 h.

Chromatographic purification was accomplished using flash chromatography on silica gel (SiO_2_, 0.04-0.063 mm) purchased from Machery-Nagel, with the indicated solvent system according to the standard techniques. Thin-layer chromatography (TLC) analysis was performed on pre-coated Merck TLC plates (silica gel 60 GF254, 0.25 mm). Visualization of the developed chromatography was performed by checking UV absorbance (254 nm and 365 nm) as well as with phosphomolybdic acid and potassium permanganate aqueous solutions. Organic solutions were concentrated under reduced pressure on a Büchi rotary evaporator.

All commercial grade reagents and solvents were purchased at the highest commercial quality from Sigma Aldrich and used as received, unless otherwise stated.

## A.1. Electrochemical characterization procedures by cyclic voltammetry

A typical three-electrode cell was employed, combining a glassy carbon (GC) working electrode (WE) (BioLogic, 3 mm nominal diameter, 7 mm^2^ geometric area), a platinum electrode (BASi) as counter electrode (CE) and a silver/silver chloride electrode (Ag/AgCl/3M NaCl) as reference electrode (RE). Oxygen was removed by saturating the solution with high-purity Nitrogen (N_2_) or carbon dioxide (CO_2_). The glass electrochemical cell was kept closed during the measurements, the headspace of the cell being also degassed to prevent dioxygen contamination.

Unless otherwise stated, experiments were conducted in acetonitrile (CH_3_CN), with 0.1 M tetrabutylammonium hexafluorophosphate (TBAPF_6_) at room temperature, and with a scan rate of 0.1 V∙s^–1^. All potentials were then converted to ferrocenium/ferrocene (Fc^+^/Fc), using an internal reference system^1^, upon addition, at the end of each experiment session, of ferrocene to the analyte solutions, and running a cyclic voltammogram from which the E_1/2_ of the couple was measured.

The glassy carbon working electrode was polished before any measurement with a 1 μm diamond paste on a microfiber cloth (Struers), carefully rinsed with de-ionized water, then methanol and rinsed by applying ultrasonic for 5 minutes. After each series of CV experiments, the electrochemical cell was carefully rinsed with ethanol, acetone, and de-ionized water; afterwards, the cell and the magnetic stirrer were sonicated for 5 min with acetone.

## A.2. Esterification procedure

10 mL of electrolysis solution (initially containing 0.2 mmol of substrate) were evaporated under vacuum and then dissolved in 1.5 mL of anhydrous methanol. 66 µL of concentrated sulfuric acid were added and the mixture was placed in a microwave reactor setting the bulk temperature at 80°C for 1 h, with constant stirring. Reaction progress was controlled by TLC (Hexane: Ethyl acetate 4:6 + 2 % acetic acid).

After the MW induced heating, the reaction mixture was treated with water (10 mL) and then extracted adding 3x10 mL of ethyl acetate; the organic phase was finally washed with water and then dried with anhydrous MgSO_4_ before evaporating the solvent. For quantification of the product by GC analysis, the solid was dissolved in acetonitrile, using the same amount as the initial volume of electrolysis solution esterified (10 mL or 5 mL), in order to reach the same initial concentration as in the crude electrolysis solution.

## A.3. GC analysis

Quantitative Gas chromatography (GC) analysis were carried out for evaluation of starting material conversion and ester product yield.

500 μL aliquot of the reaction mixture, was transferred into a 1.5 mL screw thread GC vial, containing 500 μL of a mesitylene solution (10 mM in CH_3_CN) as an internal standard.

The response factor of the initial substrate was used also to quantify the ester product, since the presence of one -COOCH_3_ additional group with respect to the initial substrate is expected to have a minor effect in the FID response.

The quantification was done by evaluating the ratio between the areas of the analyte and that of the standard and using this ratio value in the equation *(S1).* From the know value of the internal standard (mesitylene), the concentration of the analyte can be derived.

$\frac{A_{analyte}}{A_{STD}} =f \frac{{mol}_{analyte}}{{mol}_{STD}}$ *(S1)*

### A.3.1. Flavone calibration curve

Calibration curve of flavone by GC-FID chromatography was obtained analysing three solutions in acetonitrile with constant concentration of mesitylene (10 mM) and three different concentrations of flavone, obtained by subsequent dilution of a 20 mM flavone stock solution; these were prepared by adding in a GC vial: 500 µL of mesitylene (5 μmol); 500, 250 or 100 µL of the flavone stock solution, (corresponding to 10, 5 and 2 μmol of flavone, respectively); the corresponding volume of acetonitrile (0, 250 and 400 µL, respectively) to reach a 1 mL total volume. The samples were analysed at the GC, collecting the area of the peaks of flavone and of the standard. The resulting GC-FID calibration curve is depicted below.


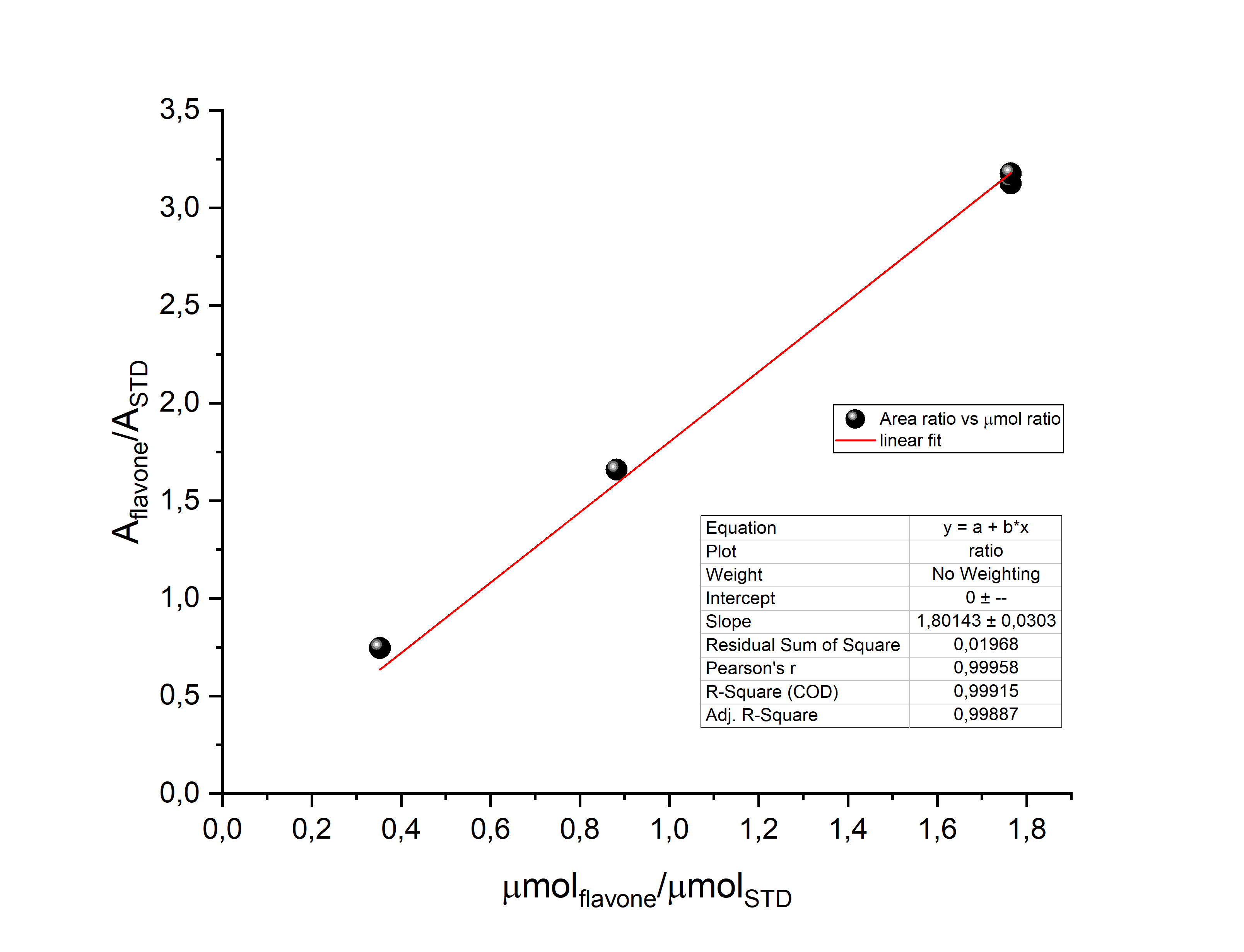


Calibration curve used to evaluate the flavone conversion after the electrosynthesis experiments and to evaluate the methyl ester yield.

Linear fitting of the experimental data provided the following equation *(S2)*:

$\frac{A_{F}}{A_{STD}} = 1.80 \frac{{\mu mol}_{F}}{{\mu mol}_{STD}}$ *(S2)*

Which can be converted into equation *(S3)* used to quantify the analyte:

$\frac{A_{F}}{A_{STD}} \frac{{\mu mol}_{STD}}{1.80}= {\mu mol}_{F}$ *(S3)*

### A.3.2. Chalcone calibration curve

Calibration of chalcone was carried out by GC-FID chromatography, using the same analytical procedure described in the previous section for flavone.

The resulting GC-FID calibration curve is depicted below.


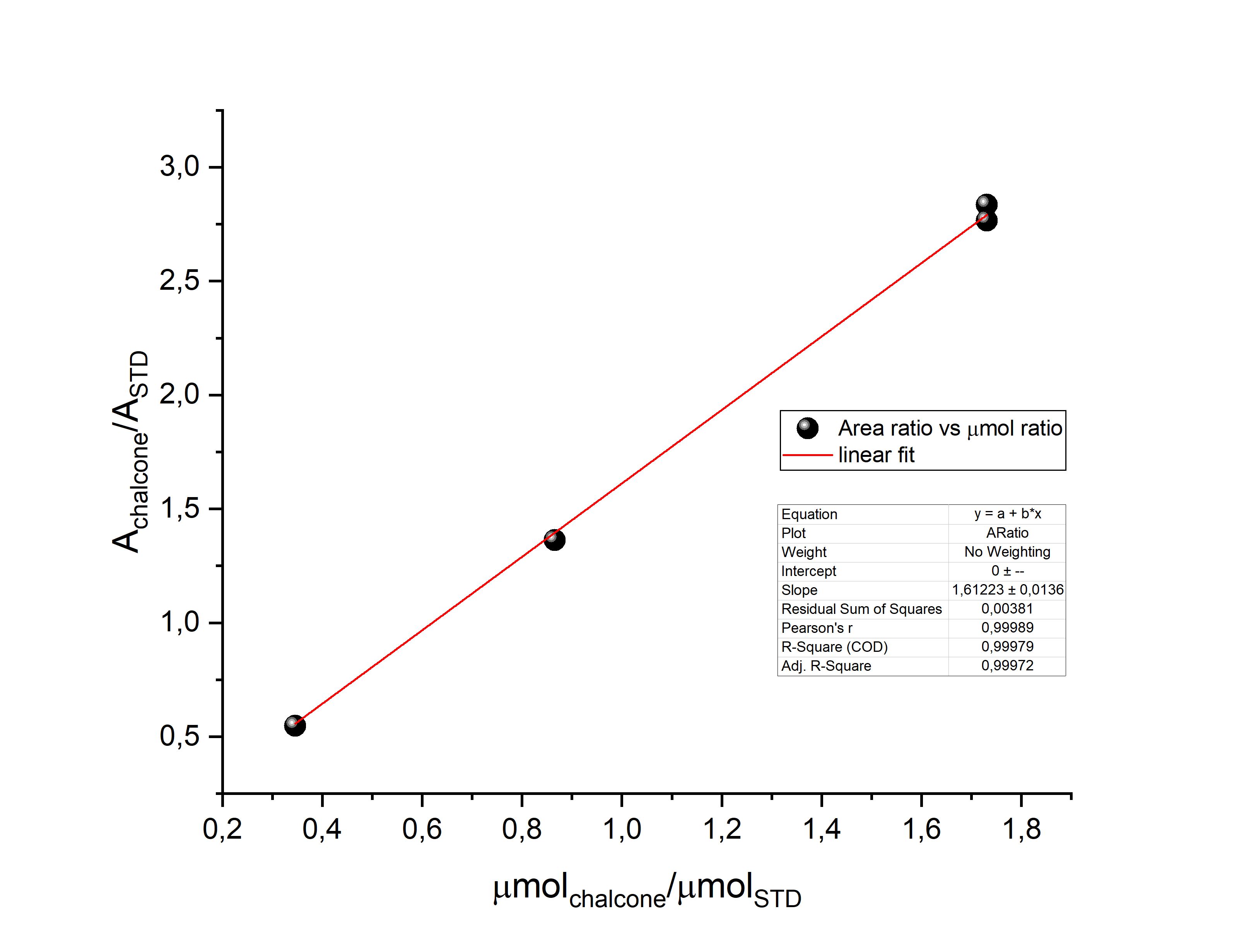


Calibration curve used to evaluate the chalcone conversion after the electrosynthesis experiments and to evaluate the methyl ester yield.

Linear fitting of the experimental data provided the following equation *(S3)*, in which the intercept was fixed at 0 value:

$\frac{A_{C}}{A_{STD}} = 1.61 \frac{{\mu mol}_{C}}{{\mu mol}_{STD}}$ *(S4)*

Which can be converted into equation *(S5)* used to quantify the analyte:

$\frac{A_{C}}{A_{STD}} \frac{{\mu mol}_{STD}}{1.61}= {\mu mol}_{C}$ *(S5)*

## A.4. DFT calculations

The DFT calculations were done on 50 small organic molecules, containing C-H moieties. For every molecule considered, geometry optimization and calculation of free energy were performed on: C-H (neutral charge, singlet states); C^−^ (negative charge, singlet states); C-COO^−^ (negative charge, singlet states).

All calculations were performed with GaussView 6, a graphical interface software by which it is possible to build a Gaussian input and interpret a Gaussian output. For all molecules, geometry optimisations and frequency calculations were done to give the Gibbs energies, using the density functional theory (DFT) method B3LYP, the 6-311G basis set and (d, p) polarization functions. The self-consistent reaction field ([SCRF](https://gaussian.com/scrf)) was used with DFT energies, optimizations, and frequency calculations to model systems in acetonitrile solution. The command expressed in all computed jobs in this work was: # opt freq b3lyp/6-311g(d,p) scrf=(solvent=acetonitrile).

# B. General Procedures For Electrolysis

Constant potential electrolysis experiments were performed in a custom-made 6-necked 2-compartment glass cell, the two compartments being separated by a porous glass frit under stirring conditions of the solution with a magnetic stirring bar.

Cathodic compartment: 20 mM of substrate in 25 ml CH_3_CN with 0.1 M tetrabutylammonium hexafluorophosphate as supporting electrolyte (except otherwise specified); glassy carbon rod working electrode (SIGRADUR, HTW, ca 1.5 cm^2^ geometric area); Ag/AgCl (NaCl 3 M) as reference electrode. The reference electrode was separated from the bulk solution by means of a salt bridge (equipped with a Coralpor frit) filled with electrolyte solution. The reference electrode was fit inside the upper part of the bridge. This latter was constituted by a screw joint allowing the content of the bridge to be sealed by means of a PTFE O-ring.

Anodic compartment: 0.1 M TBAPF_6_ solution (10 ml in CH_3_CN) with a sacrificial electron donor (triethylamine, 10 eq., i.e. 200 mM); Platinum coiled wire as counter electrode.

After saturating the electrolyte solution in both compartments with CO_2_ for ca 20 min a CO_2_ atmosphere was kept above the solutions during the electrolysis experiments.


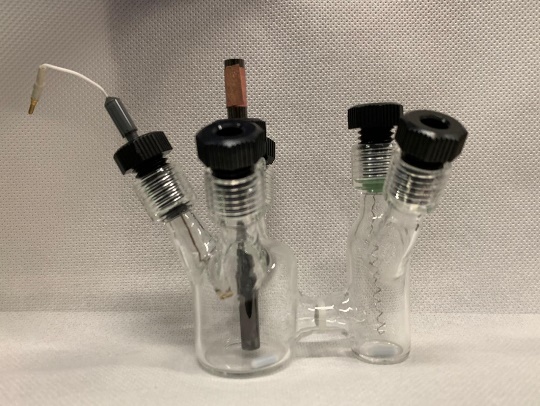


The two-compartment cell used for electrosynthesis experiments.

## B.1. Procedures for Flavanone-2-carboxylic acid methyl ester synthesis

### B.1.1 WE potential: -2.56 V vs Fc^+^/Fc

Following the general electrolysis procedure in a two-compartment cell (**section B, Set-up of the electrolysis in two-compartment cell**), Flavanone-2-carboxylic acid methyl ester was synthesized from flavone (**F**) (20 mM of **F** in 25 ml CH_3_CN with 0.1 M tetrabutylammonium hexafluorophosphate as supporting electrolyte). The experiment started with an initial current of – 12 mA. The experiment was stopped at *Q*= −99.5 C with a final current of – 3 mA. Conversion, evaluated by GC, of **F** was 60%. 10 mL of crude electrolysis solution were esterified following the procedure in **section A2**. The ester was isolated by a flash chromatographic column on silica (eluent mixture: Hexane/Ethyl acetate; gradient elution chromatography 9:1 🡪 8:2), yielding pure Flavanone-2-carboxylic acid methyl ester (yellow oil) in 55% yield (34.05 mg, 0.120 mmol).

**^1^H NMR** (300 MHz, Chloroform-*d*) δ 7.83 (ddd, *J* = 7.8, 1.8, 0.5 Hz, 1H), 7.62 – 7.56 (m, 2H), 7.52 (ddd, *J* = 8.4, 7.2, 1.8 Hz, 1H), 7.42 – 7.30 (m, 3H), 7.21 (ddd, *J* = 8.4, 1.1, 0.5 Hz, 1H), 7.03 (ddd, *J* = 7.8, 7.2, 1.1 Hz, 1H), 3.63 (s, 3H), 3.59 (d, *J* = 16.8 Hz, 1H), 3.27 (d, *J* = 16.8 Hz, 1H). **EI-MS:** 51, 77, 103, 121, 139, 165, 194.1, 223.1, 250, 282.1 (M^•+^) m/z.

### B.1.2 WE potential: -2.21 V vs Fc^+^/Fc

Following the general electrolysis procedure in a two-compartment cell, Flavanone-2-carboxylic acid methyl ester was synthesized from flavone **F** (20 mM of **F** in 25 ml CH_3_CN with 0.1 M tetrabutylammonium hexafluorophosphate as supporting electrolyte). The experiment started with an initial current of – 9.5 mA. The experiment was stopped at *Q*= −71 C with a final current of – 1.7 mA. Conversion, evaluated by GC, of **F** was 65%. The crude solution become red when evaporated, during work up. 10 mL of crude electrolysis solution were esterified (GC yield for flavanone-2-carboxylic acid methyl ester: 3%). By-product were obtained:

**2,2-biflavanone meso**

**^1^H NMR** (300 MHz, Chloroform-*d*): δ 7.72 – 6.76 (m, 18H), 3.62 (d, J = 16.1 Hz, 1H), 3.10 (d, J = 16.2 Hz, 1H). **ESI-MS [MH^+^]:** 447.1 m/z.

**2,2-biflavanone racemate**

**^1^H NMR** (300 MHz, Chloroform-*d*): δ 7.72 – 6.76 (m, 18H), 3.93 (d, J = 16.3 Hz, 1H), 3.33 (d, J = 16.3 Hz, 1H). **ESI-MS [MH^+^]:** 447.1 m/z.

**ESI mass spectrum:** [MH]^+^: 447.1 m/z. The intense peak in ESI-MS at 242.3 m/z was attributed to TBA^+^ ion.

|  |
| --- |
|  |

## B.2. Procedure for Methyl 4-oxo-2,4-diphenylbutanoate synthesis

### B.2.1 WE potential: -2.7 V vs Fc^+^/Fc

Following the general electrolysis procedure in a two-compartment cell, Methyl 4-oxo-2,4-diphenylbutanoate was synthesized from *trans*-Chalcone **C** (20 mM of **C** in 25 ml CH_3_CN with 0.1 M tetrabutylammonium hexafluorophosphate as supporting electrolyte). The experiment started with an initial current of – 26 mA. The experiment was stopped at *Q*= −100 C with a final current of – 9 mA. Conversion of **C** (by GC)**:** 79.5%. 5 mL of crude electrolysis solution were subject to esterification. The GC yield of the esterification crude sample before the chromatographic separation give a 41% yield. Ester was isolated by a flash chromatographic column on silica (eluent mixture: Hexane/Ethyl acetate; gradient elution chromatography 9:1 🡪 8:2), yielding pure Methyl 4-oxo-2,4-diphenylbutanoate (yellow oil) in 37% isolated yield (10 mg, 0.037 mmol).

**^1^H NMR** (300 MHz, Chloroform-*d*) δ 8.09 – 7.81 (m, 2H), 7.64 – 7.17 (m, 8H), 4.29 (dd, J = 10.3, 4.1 Hz, 1H), 3.93 (dd, J = 18.0, 10.3 Hz, 1H), 3.68 (s, 3H), 3.26 (dd, J = 18.0, 4.1 Hz, 1H). **EI-MS:** 51, 77, 105, 130.9, 208, 236, 268 (M^•+^) m/z.

# C. Supplementary Figures and Tables

| 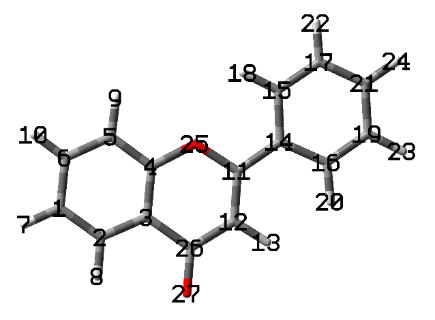  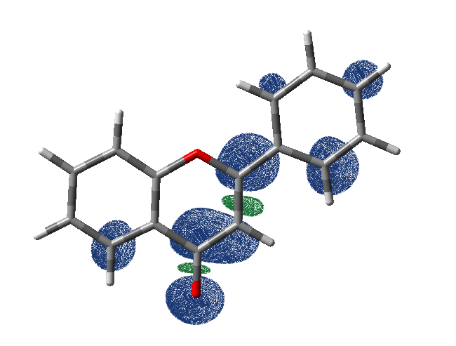 | Mulliken charges and spin densities:  1 C -0.118696 0.002780  2 C -0.096806 0.064012  3 C -0.189990 -0.033024  4 C 0.207737 -0.002592  5 C -0.135692 0.037162  6 C -0.117180 0.031565  7 H 0.101220 -0.000581  8 H 0.081225 -0.004386  9 H 0.108096 -0.002490  10 H 0.104958 -0.002177  11 C 0.191712 0.284510  12 C -0.222997 0.007405  13 H 0.064909 -0.001288  14 C -0.175583 -0.029389  15 C -0.084868 0.160764  16 C -0.118663 0.159273  17 C -0.125372 -0.079174  18 H 0.090657 -0.008958  19 C -0.129068 -0.078753  20 H 0.092885 -0.008890  21 C -0.148596 0.222954  22 H 0.094177 0.003310  23 H 0.094279 0.003339  24 H 0.091199 -0.013496  25 O -0.374373 0.033542  26 C 0.219411 0.121794  27 O -0.504581 0.132788 |
| --- | --- |

**Figure S1**: Optimized geometry of Flavone radical anion F(RA) and pictorial view of the spin density; charge = -1; multiplicity = doublet. Mulliken charges and spin density from the output file.

| 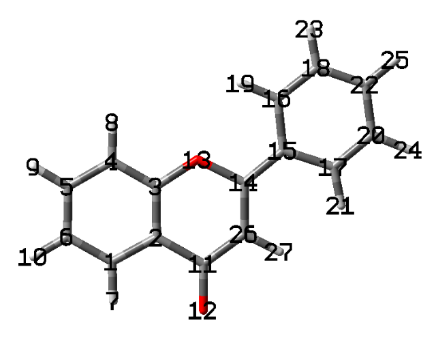 | Mulliken charges:  1 C -0.148284  2 C -0.182037  3 C 0.205168  4 C -0.185914  5 C -0.137678  6 C -0.139824  7 H 0.055579  8 H 0.084544  9 H 0.085331  10 H 0.081825  11 C 0.167176  12 O -0.600910  13 O -0.399792  14 C 0.114273  15 C -0.158762  16 C -0.173622  17 C -0.196036  18 C -0.136980  19 H 0.054180  20 C -0.142162  21 H 0.059818  22 C -0.247730  23 H 0.063160  24 H 0.063474  25 H 0.053822  26 C -0.263186  27 H 0.024567 |
| --- | --- |

**Figure S2**: Optimized geometry of Flavone dianion **F(DA)**; charge = -1; multiplicity = singlet. Mulliken charges from the output file.


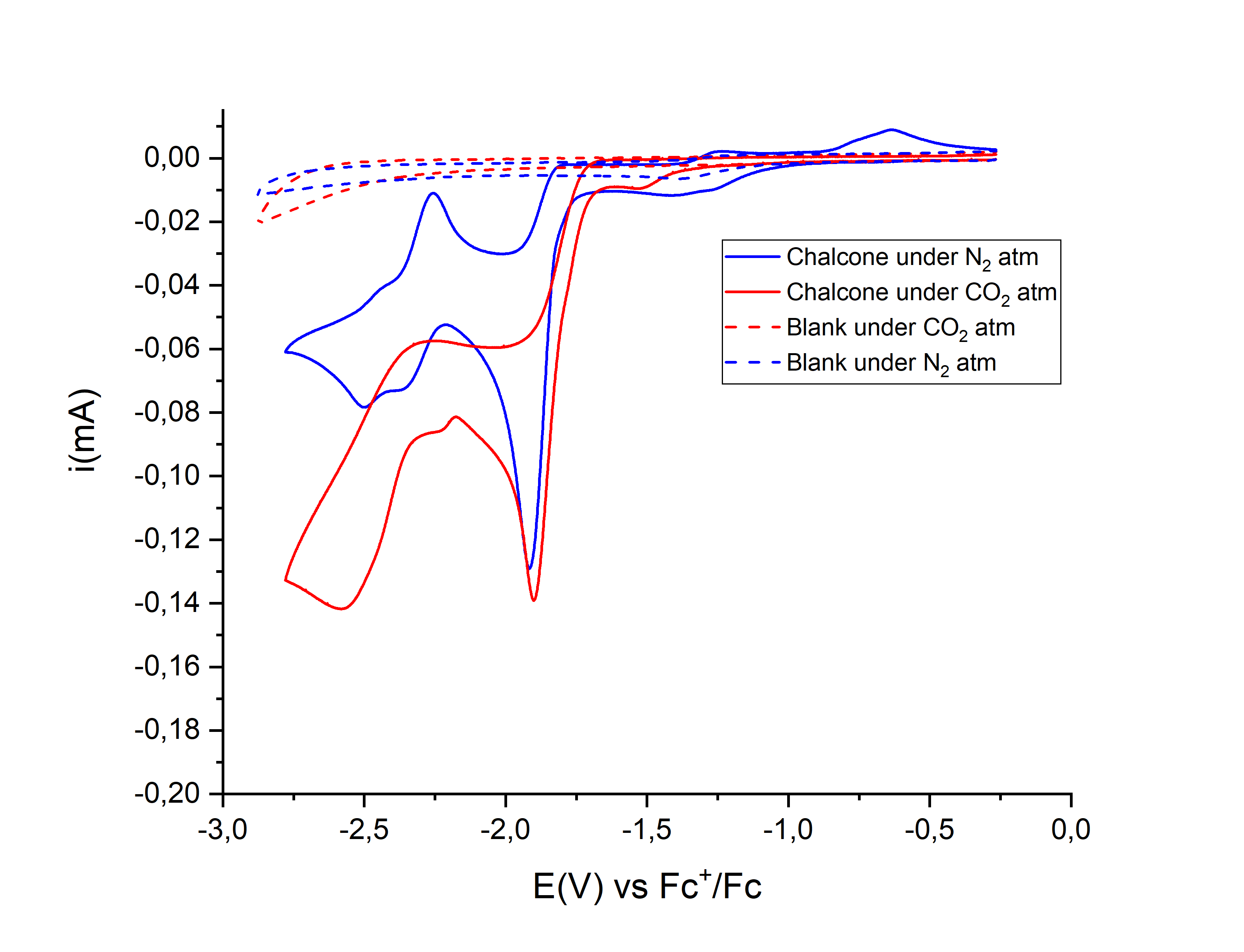


**Figure S3**. Cyclic voltammetries of *trans*-Chalcone (5 mM in CH_3_CN) under N_2_ and CO_2_ atmosphere (blue and red traces, respectively). Scan rate: 0.1 V∙s^–1^. 0.1 M TBAPF_6_ was used as supporting electrolyte. Working electrode: Glassy Carbon; Counter electrode: Platinum; Reference electrode: Ag/AgCl (3 M NaCl). Reduction potentials are then converted vs Fc/Fc^+^ couple, registering a CV scan of a ferrocene solution under the same conditions.

| 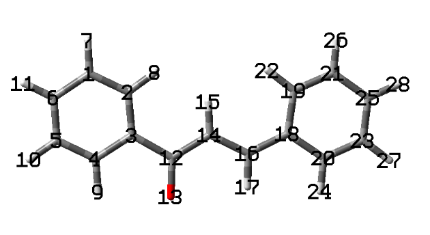  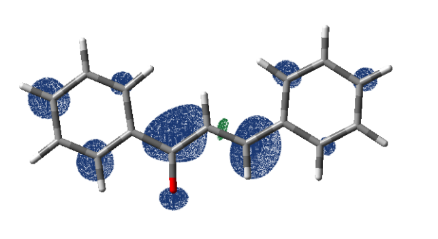 | Mulliken charges and spin densities:  1 C -0.125722 -0.030572  2 C -0.100725 0.058417  3 C -0.134496 -0.033457  4 C -0.095257 0.089361  5 C -0.115171 -0.041571  6 C -0.123967 0.092936  7 H 0.098747 0.001240  8 H 0.096378 -0.002923  9 H 0.078284 -0.005769  10 H 0.099529 0.001829  11 H 0.099089 -0.005601  12 C 0.166070 0.178102  13 O -0.506269 0.191832  14 C -0.231984 -0.006265  15 H 0.093313 -0.002204  16 C -0.129574 0.326153  17 H 0.055866 -0.019485  18 C -0.062055 -0.054562  19 C -0.126391 0.134463  20 C -0.116044 0.108963  21 C -0.119645 -0.066868  22 H 0.089959 -0.007479  23 C -0.122209 -0.057913  24 H 0.086434 -0.005704  25 C -0.142750 0.161268  26 H 0.096481 0.003025  27 H 0.096965 0.002450  28 H 0.095143 -0.009666 |
| --- | --- |

**Figure S4**. Optimized geometry of chalcone radical anion **C(RA)** and pictorial view of the spin density; charge = -1; multiplicity = doublet. Mulliken charges and spin density from the output file.

| 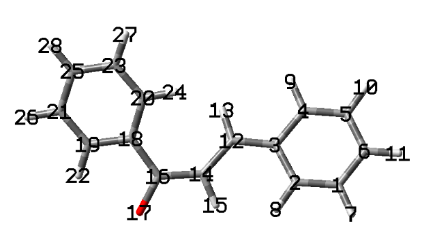 | Mulliken charges:  1 C -0.130964  2 C -0.202702  3 C -0.060555  4 C -0.178948  5 C -0.137644  6 C -0.225686  7 H 0.069439  8 H 0.063601  9 H 0.053986  10 H 0.069771  11 H 0.062681  12 C -0.218384  13 H 0.036982  14 C -0.231311  15 H 0.000865  16 C 0.105966  17 O -0.644614  18 C -0.135590  19 C -0.137806  20 C -0.120806  21 C -0.123966  22 H 0.056921  23 C -0.142808  24 H 0.080029  25 C -0.158568  26 H 0.083671  27 H 0.083784  28 H 0.082655 |
| --- | --- |

**Figure S5:** Optimized geometry of chalcone dianion **C(DA)**; charge = -1, multiplicity = singlet. Mulliken charges from the output file.

**Table S1**. Calculated ΔG^0^ for the carboxylation of double bonds (indicated in red) starting from the radical anion.

| Organic substrate | Calc. pK_a_ | calc. ΔG^0^ (Kcal/mol) |
| --- | --- | --- |
|  | 18.6 (in α)  11.8 (in β) | +19.6 (in α)  unstable (in β) |
|  | 18.5 (in α)  18.2 (in β) | +22.0 (in α)  +20.2 (in β) |
|  | 8.0 (in α)  12.0 (in β) | +28.8 (in α)  unstable (in β) |
|  |  | +15.7 (in α)  +18.3 (in β) |
|  |  | +7.8 (in α)  +9.7 (in β) |
|  |  | +17.2 (in α)  +4.1 (in β) |
|  |  | unstable |
| Organic substrate | Calc. pK_a_ | calc. ΔG^0^ (Kcal/mol) |
|  |  | +5.2 |
|  | 55.9 | -25.2 |
|  | 58.3 | -28.7 |
|  | 60.9 | -29.6 |
|  | 28.7 | +10.1 |
|  | 28.1 (in α)  42.0 (in β) | +15.8 (in α)  -6.5 (in β) |
|  | 28.6 | +10.9 |

# D. Optimized Geometries


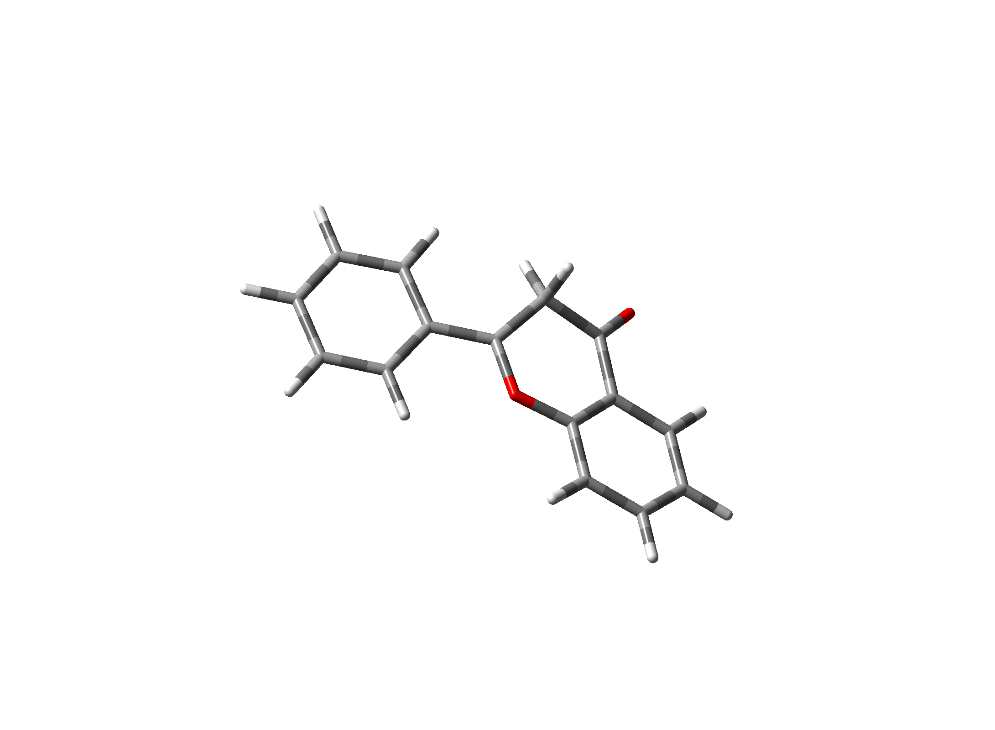


**F(RA)-Hα**: Flavone radical anion conjugate acid, considering the protonation in α position

**
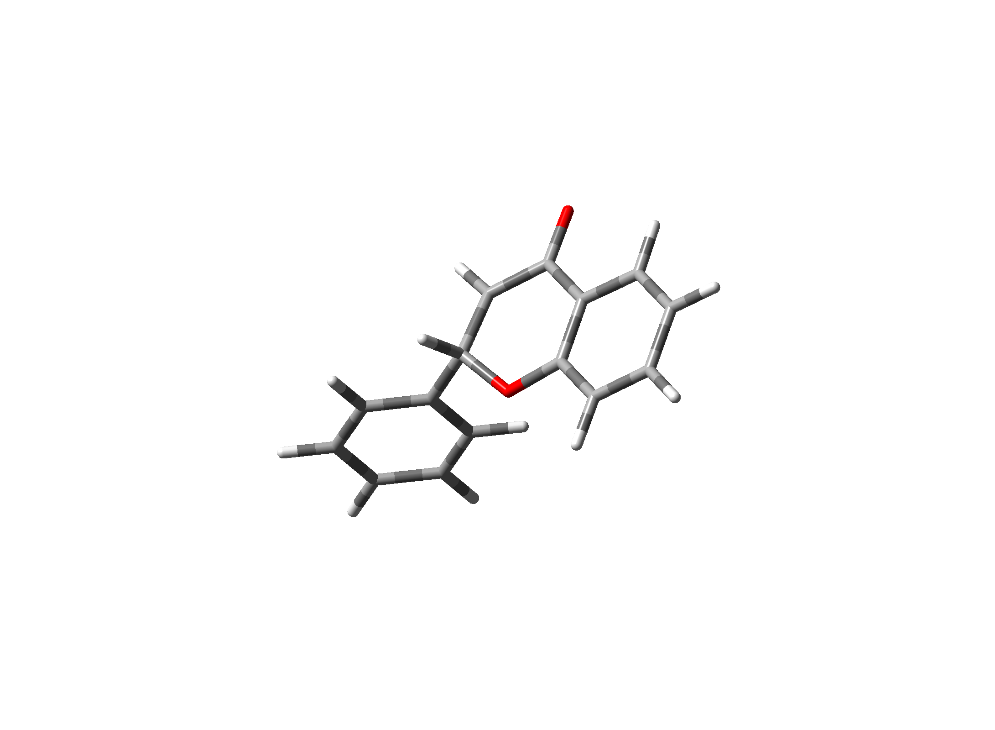
**

**F(RA)-Hβ**: Flavone radical anion conjugate acid, considering the protonation in β position

**
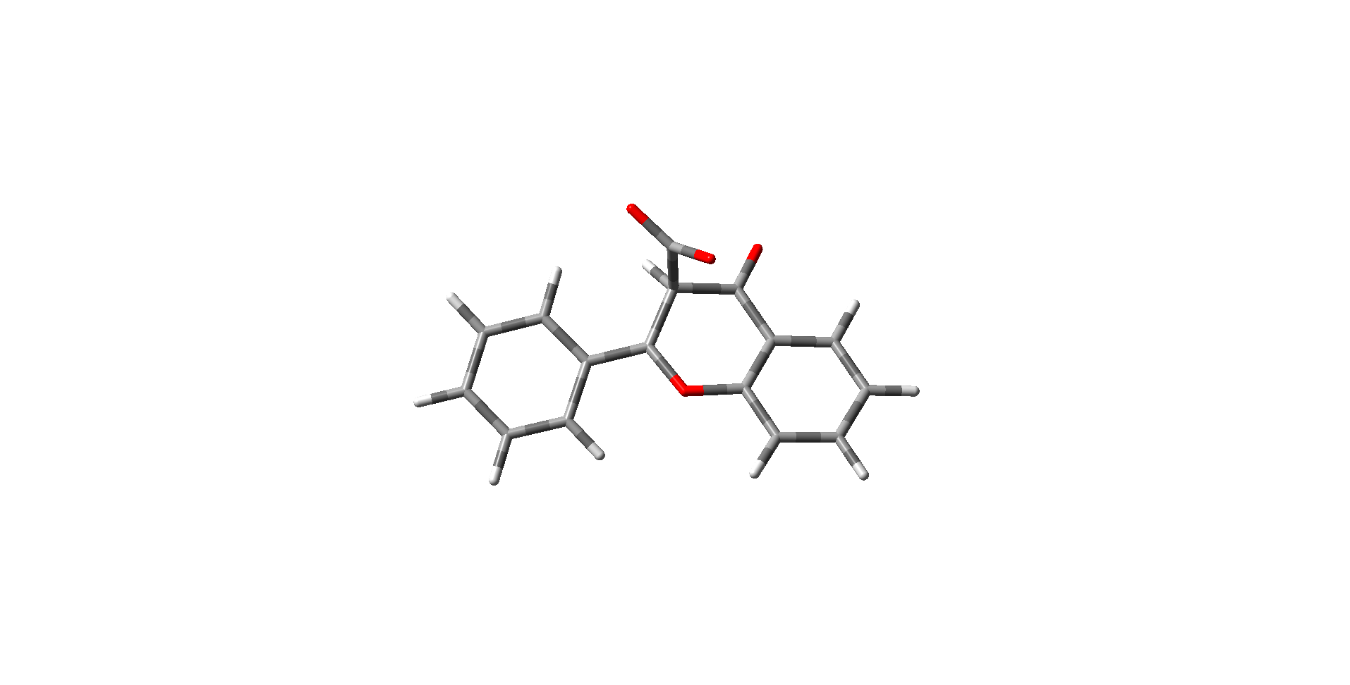
**

**F(RA)-COOα:** Flavone radical anion carboxylate product in α position

**
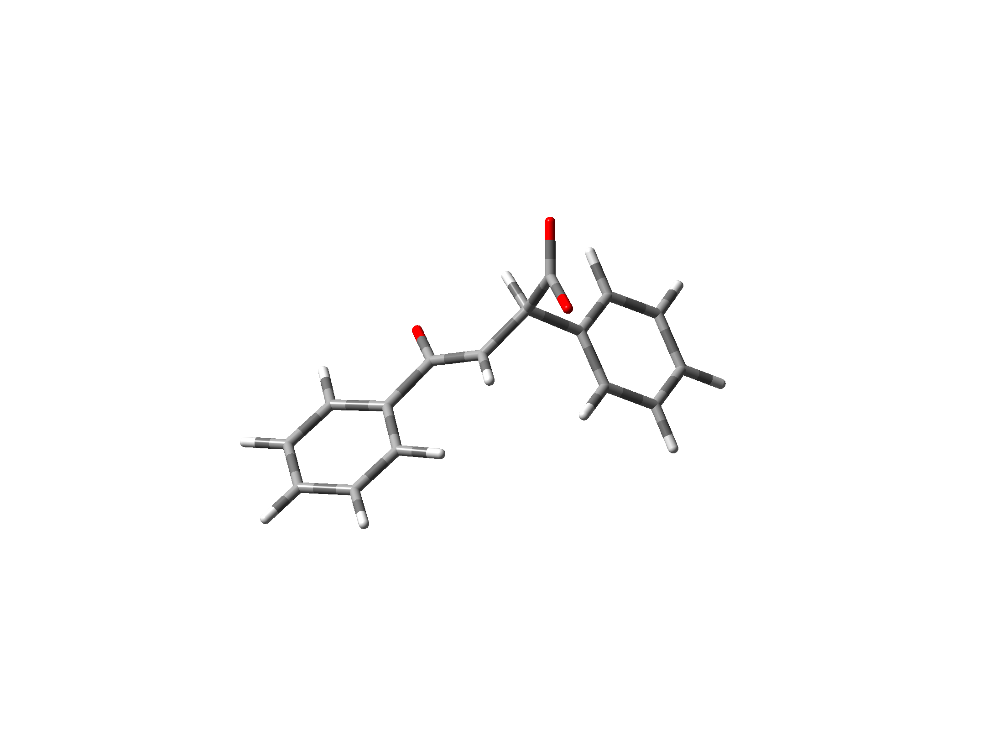
**

**F(RA)-COOβ**: Flavone radical anion carboxylate product in β position

**
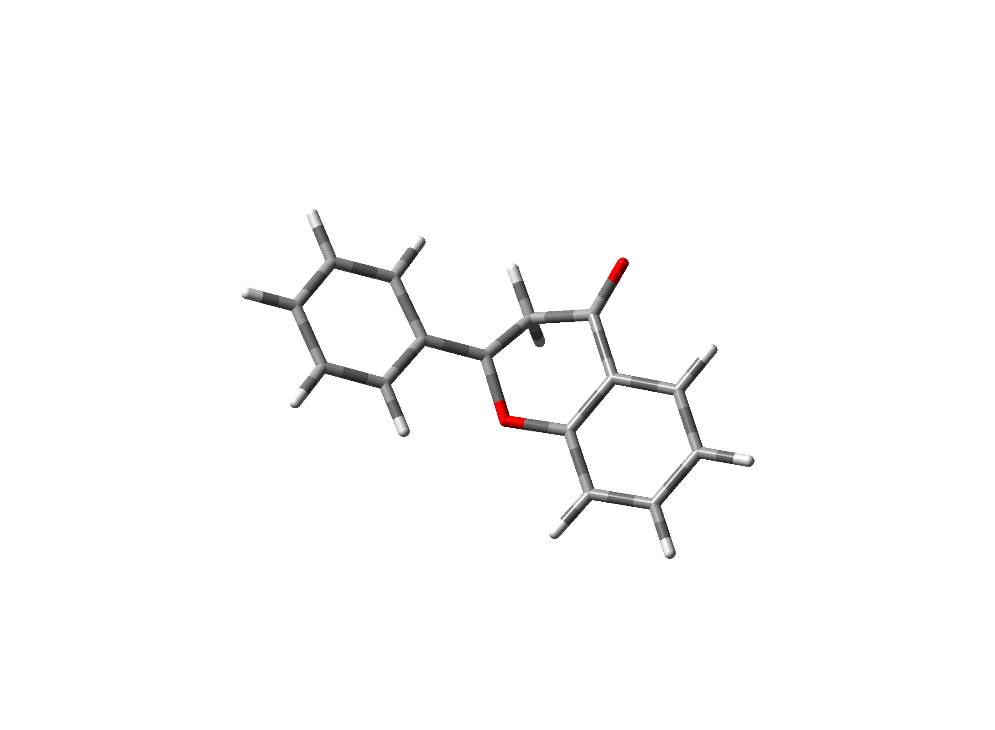
**

**F(DA)-Hα**: Flavone dianion conjugate acid, considering the protonation in α position

**
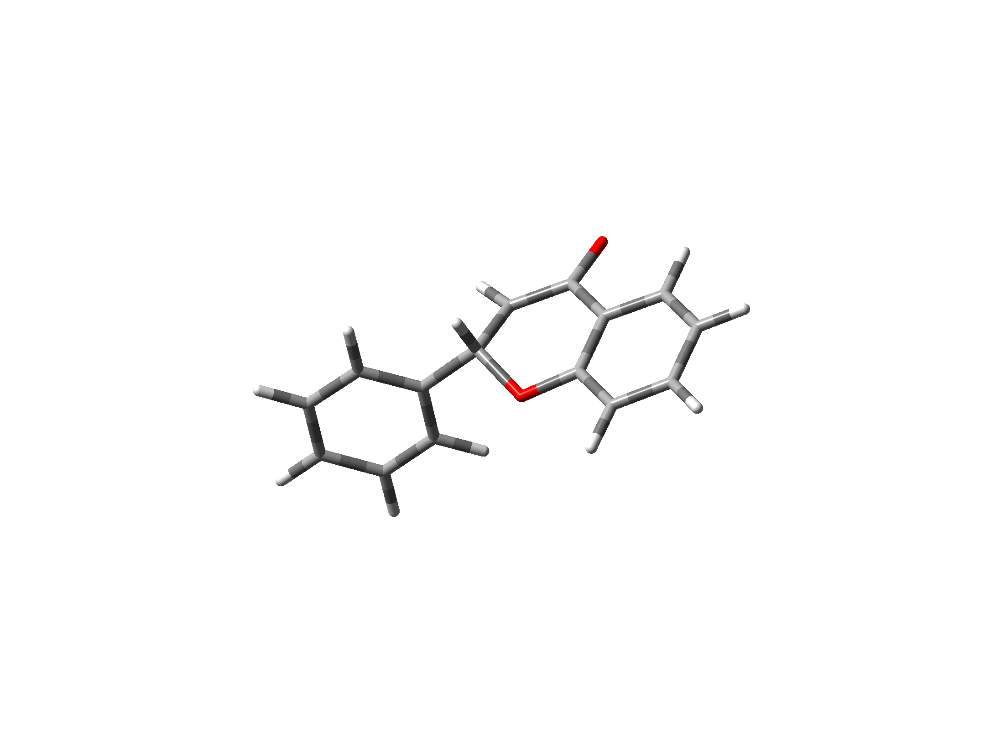
**

**F(DA)-Hβ**: Flavone dianion conjugate acid, considering the protonation in β position

**
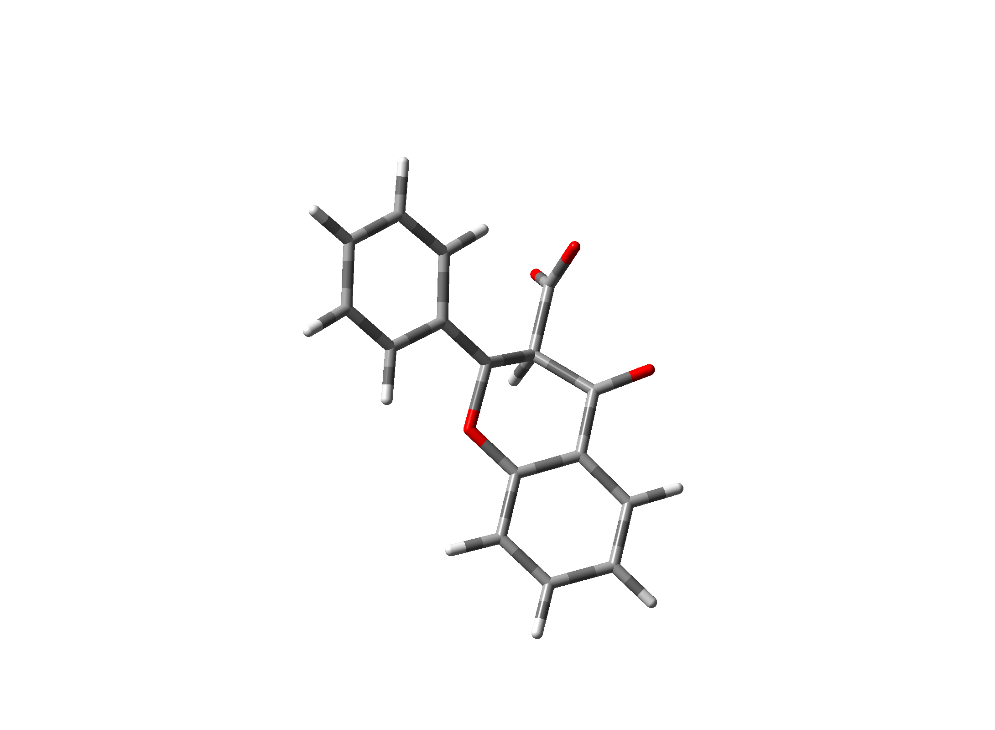
**

**F(DA)-COOα**: Flavone dianion carboxylate product in α position

**
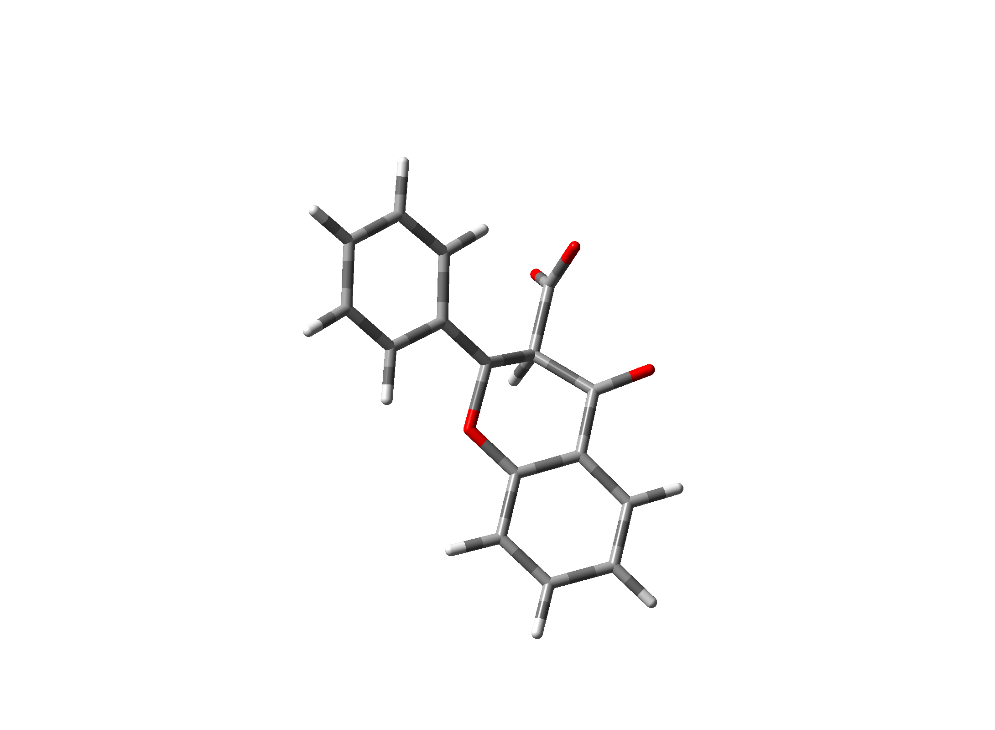
**

**F(DA)-COOβ:** Flavone dianion carboxylate product in β position

**
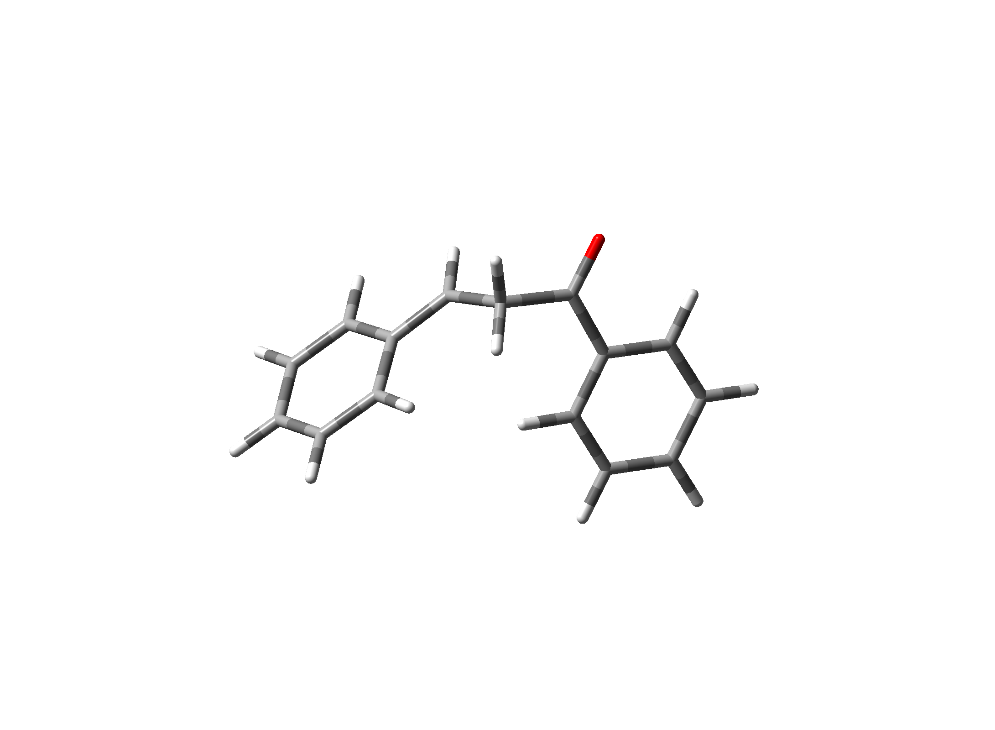
**

**C(RA)-Hα***: trans*-Chalcone radical anion conjugate acid, considering the protonation in α position

**
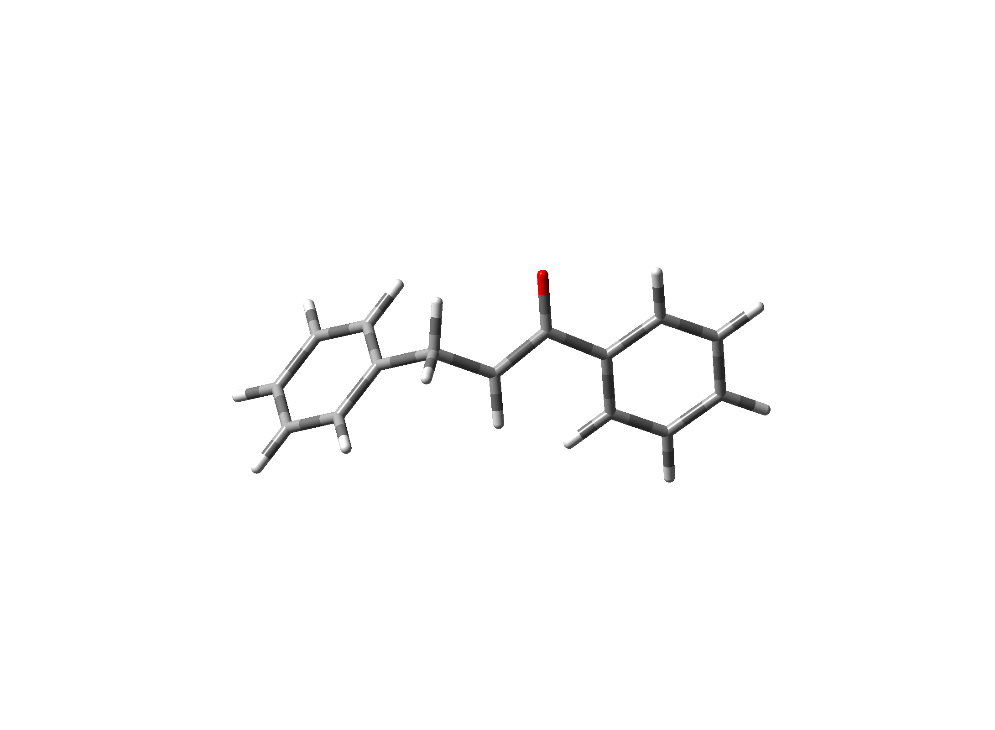
**

**C(RA)-Hβ***: trans*-Chalcone radical anion conjugate acid, considering the protonation in β position

**
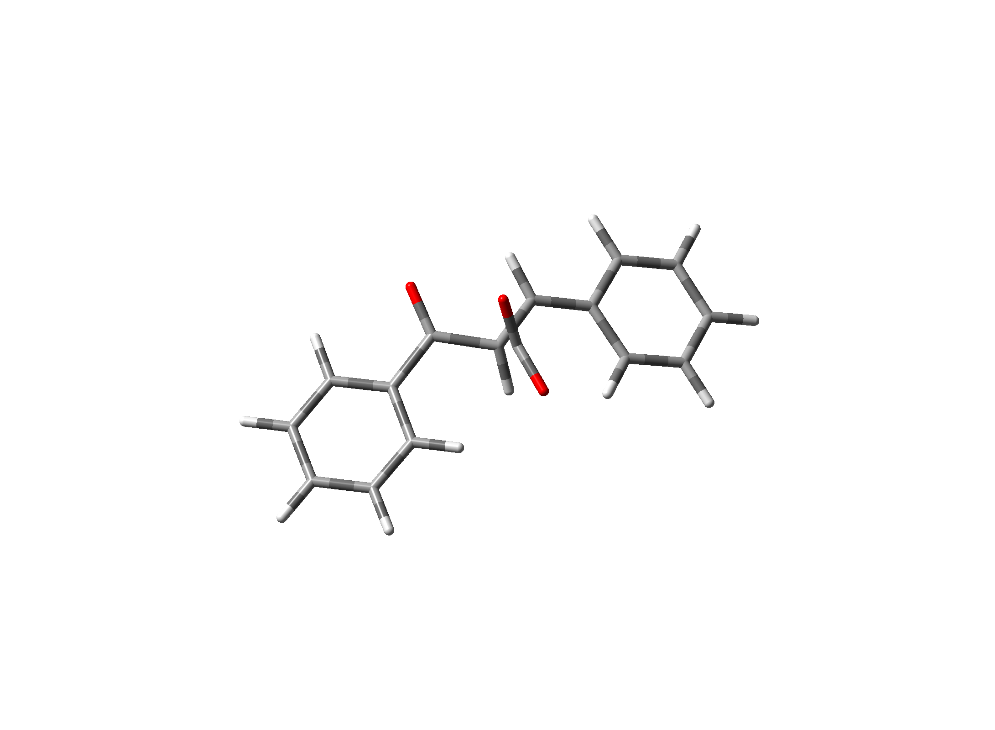
**

**C(RA)-COOα***: trans*-Chalcone radical anion carboxylate product in α position

**
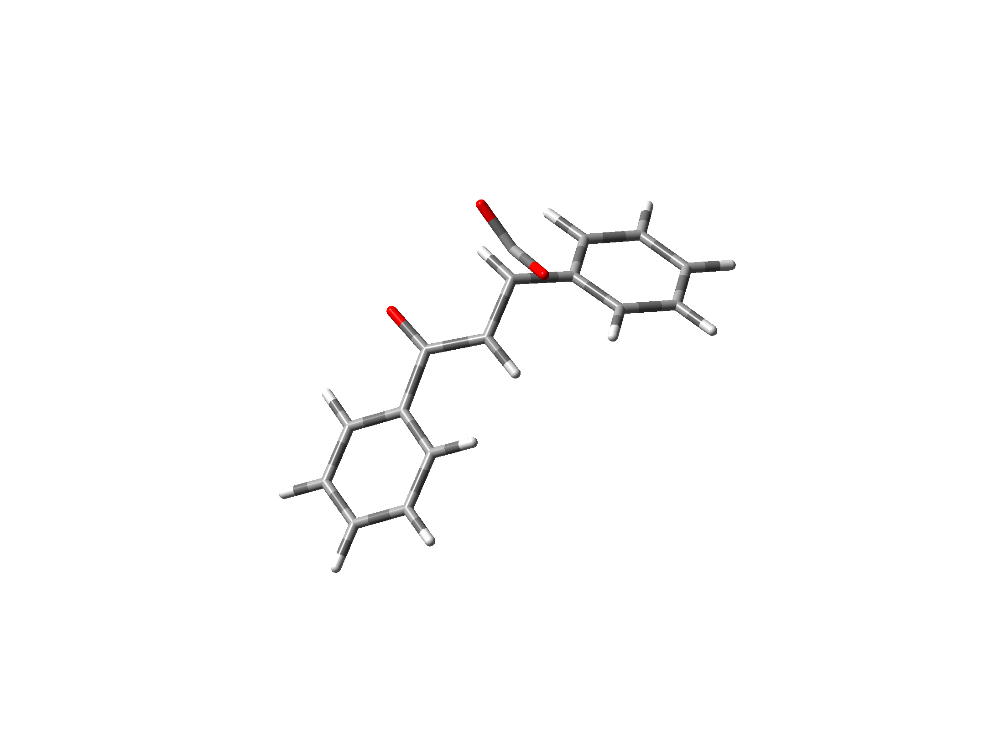
**

**C(RA)-COOβ***: trans*-Chalcone radical anion carboxylate product in β position

**
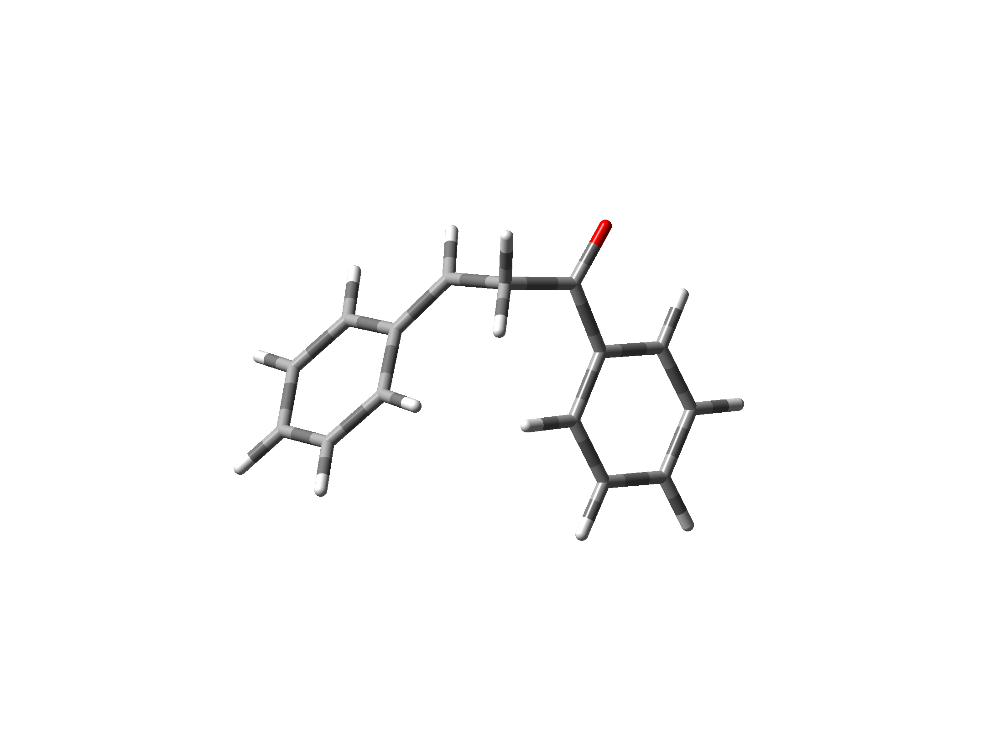
**

**C(DA)-Hα***: trans*-Chalcone dianion conjugate acid, considering the protonation in α position

**
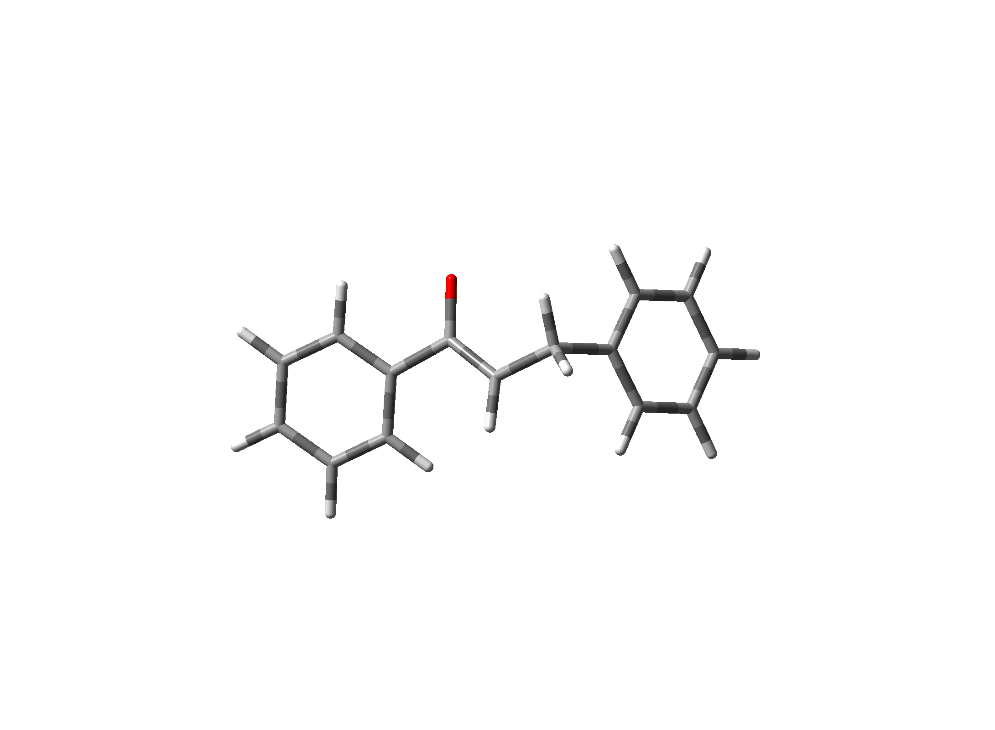
**

**C(DA)-Hβ:** *trans*-Chalcone dianion conjugate acid, considering the protonation in β position

**
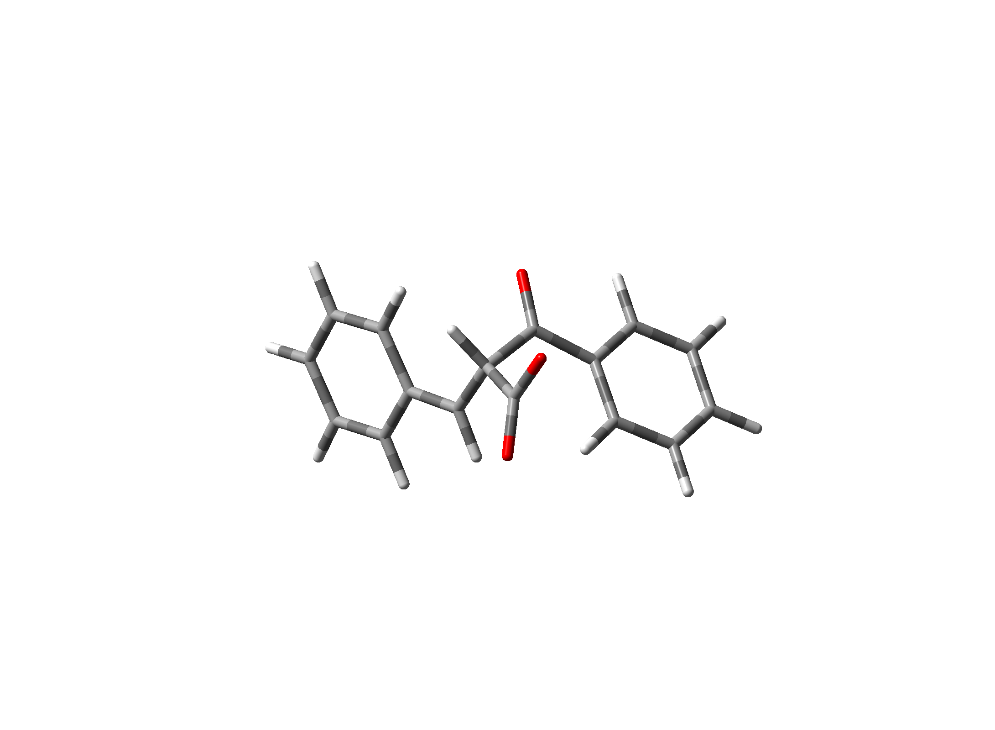
**

**C(DA)-COOα:** *trans*-Chalcone dianion carboxylate product in α position

**
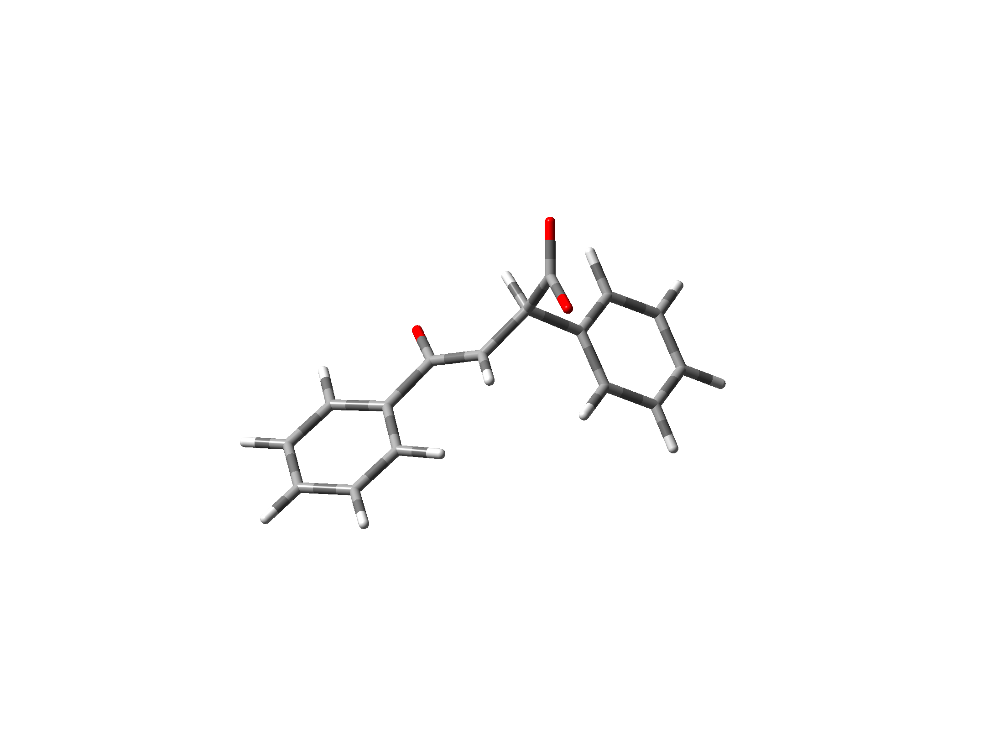
**

**C(DA)-COOβ***: trans*-Chalcone dianion carboxylate product in β position

**
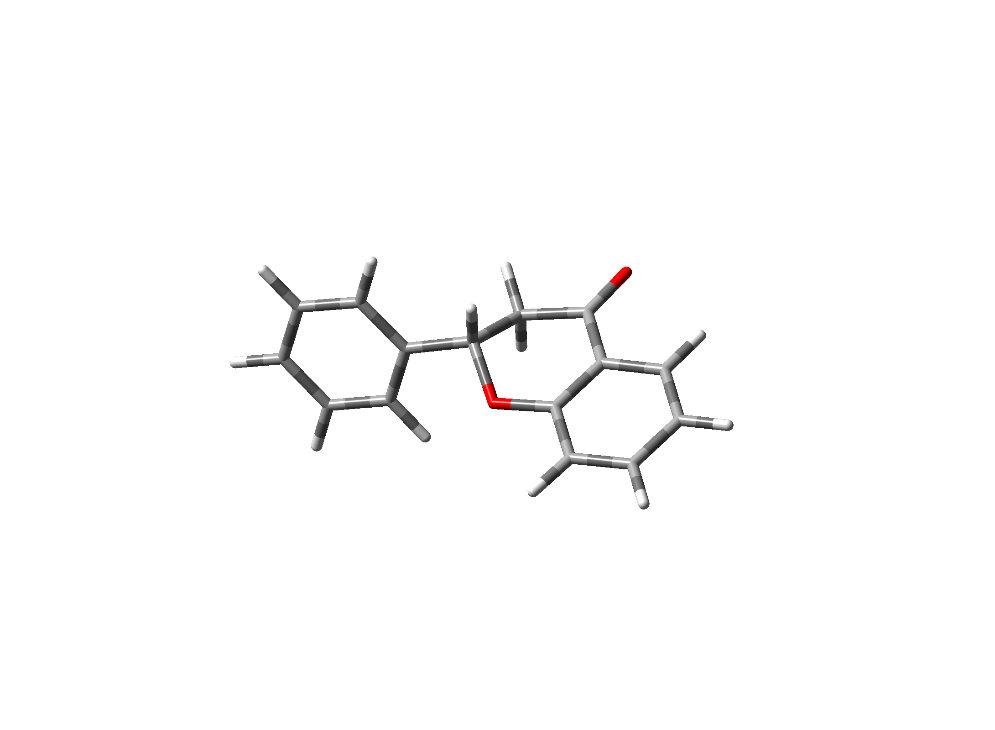
**

**F(A)-H**: Flavone anion, generated after a hydrogen atom transfer (HAT) step, conjugate acid (i.e. Flavanone)

**
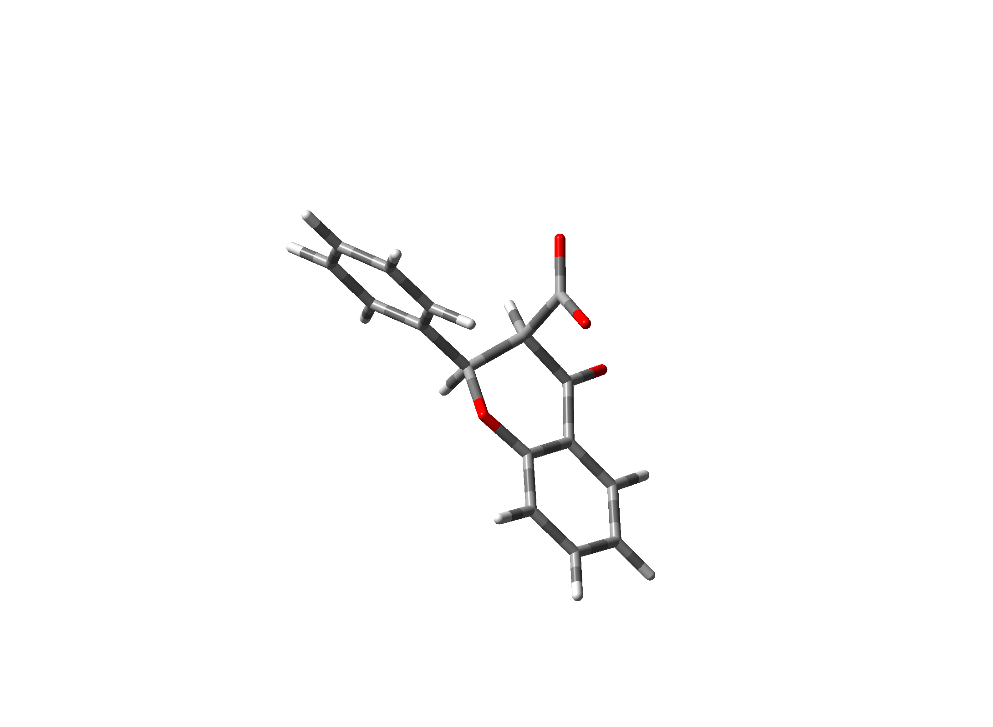
**

**F(A)-COOα**: Flavone anion, generated after a hydrogen atom transfer (HAT) step, carboxylate product in α position

**
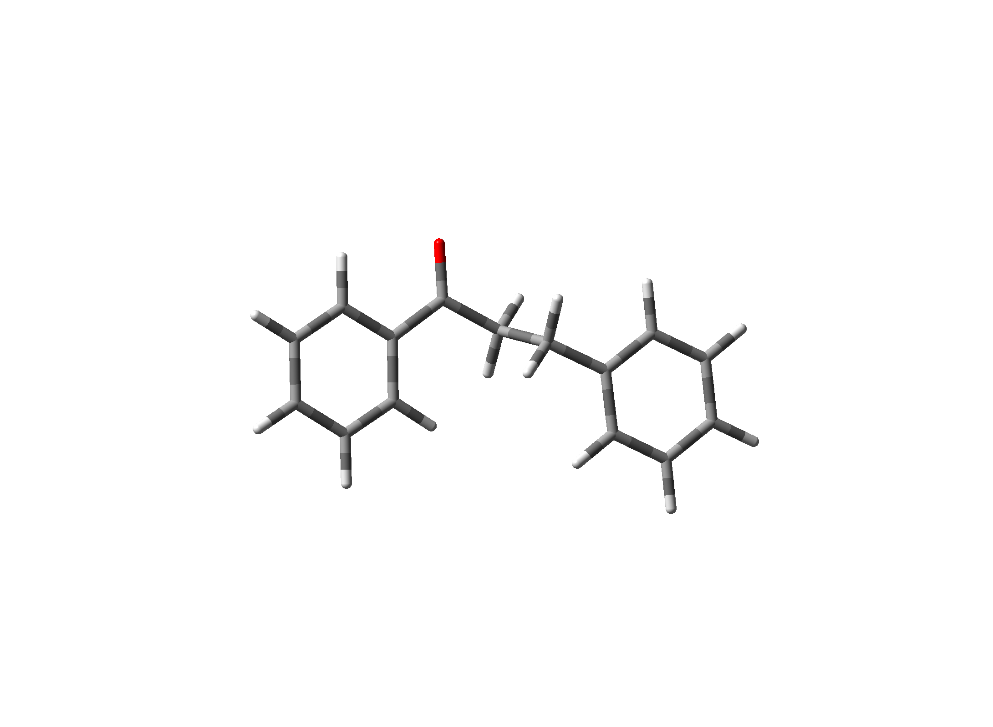
**

**C(A)-H***: trans*-Chalcone anion, generated after a hydrogen atom transfer (HAT) step, conjugate acid (i.e. 2-Phenylacetophenone)

**
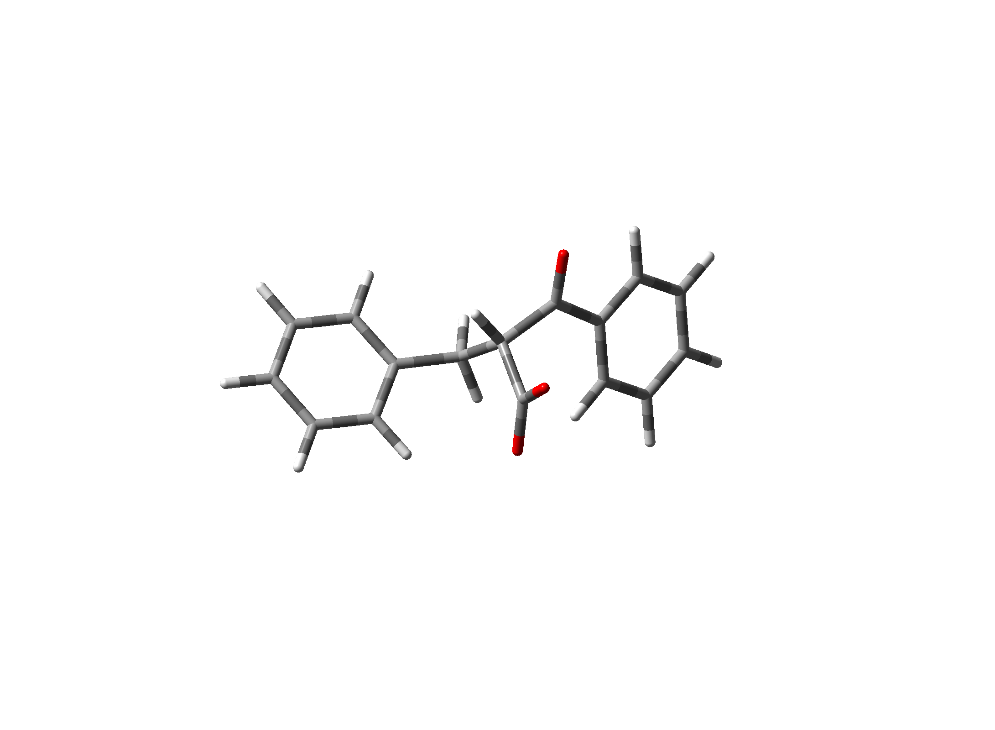
**

**C(A)-COOα:** *trans*-Chalcone anion, generated after a hydrogen atom transfer (HAT) step, carboxylate product in α position

**
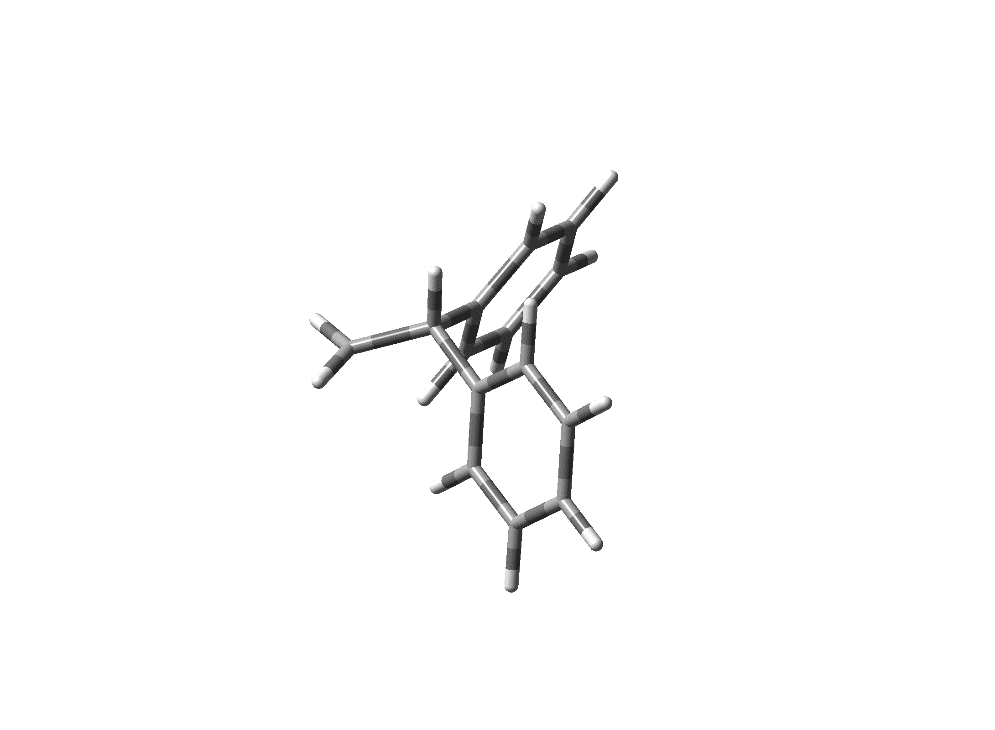
**

**1,1-DPE(RA)-Hα**: 1,1-Diphenylethylene radical anion conjugate acid, considering the protonation in α position

**
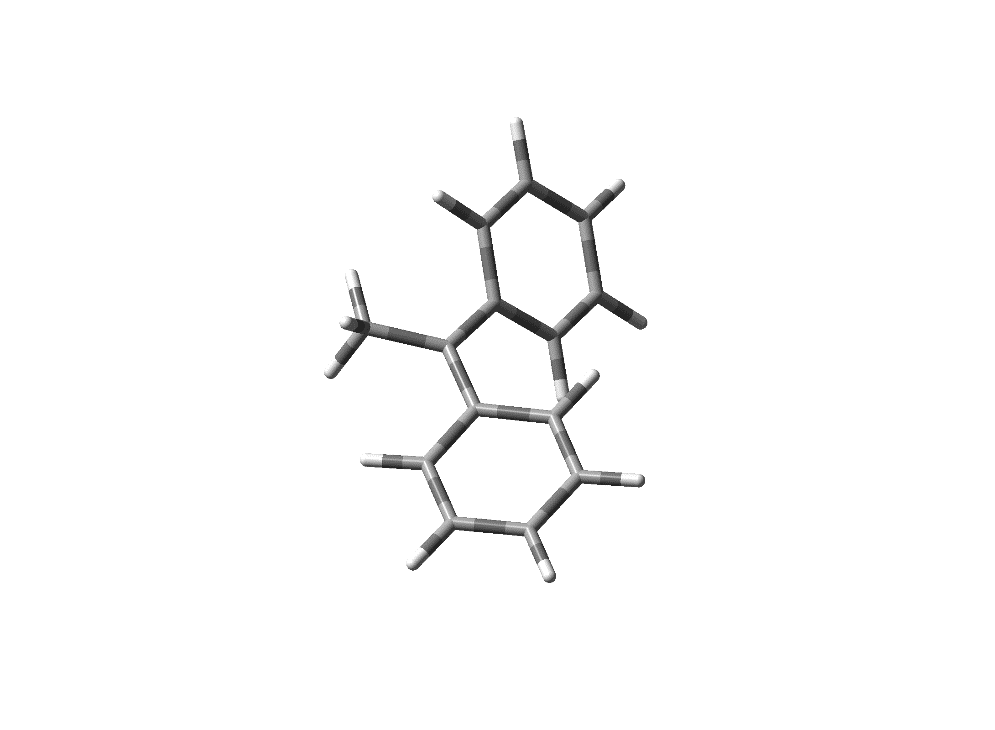
**

**1,1-DPE(RA)-Hβ**: 1,1-Diphenylethylene radical anion conjugate acid, considering the protonation in β position

**
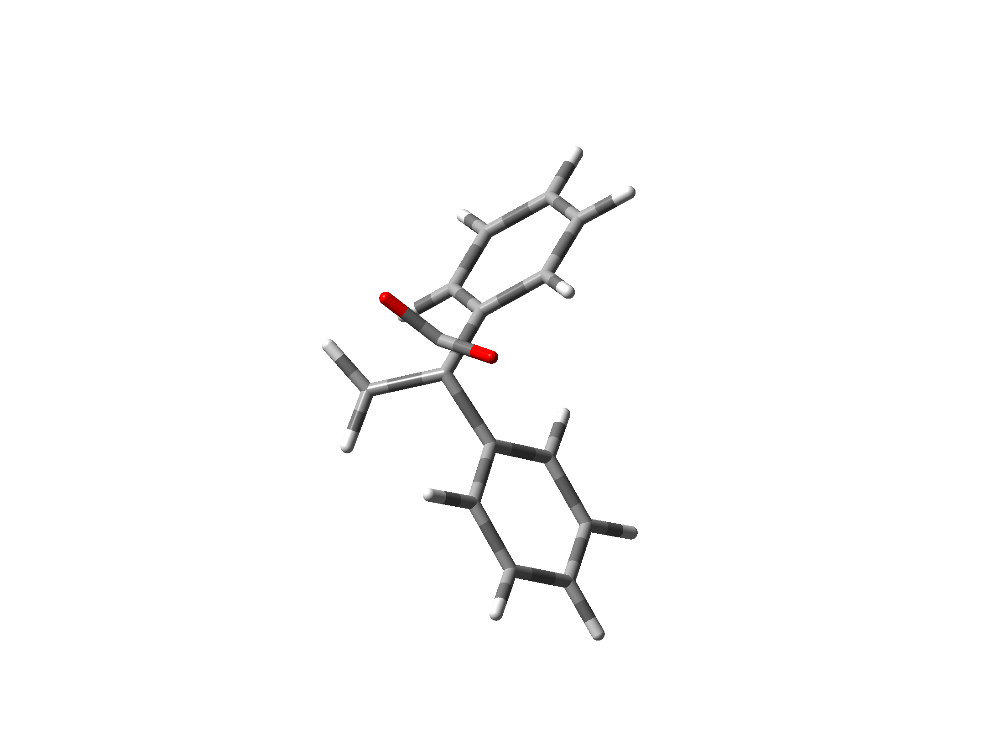
**

**1,1-DPE(RA)-COOα**: 1,1-Diphenylethylene radical anion carboxylate product in α position

**
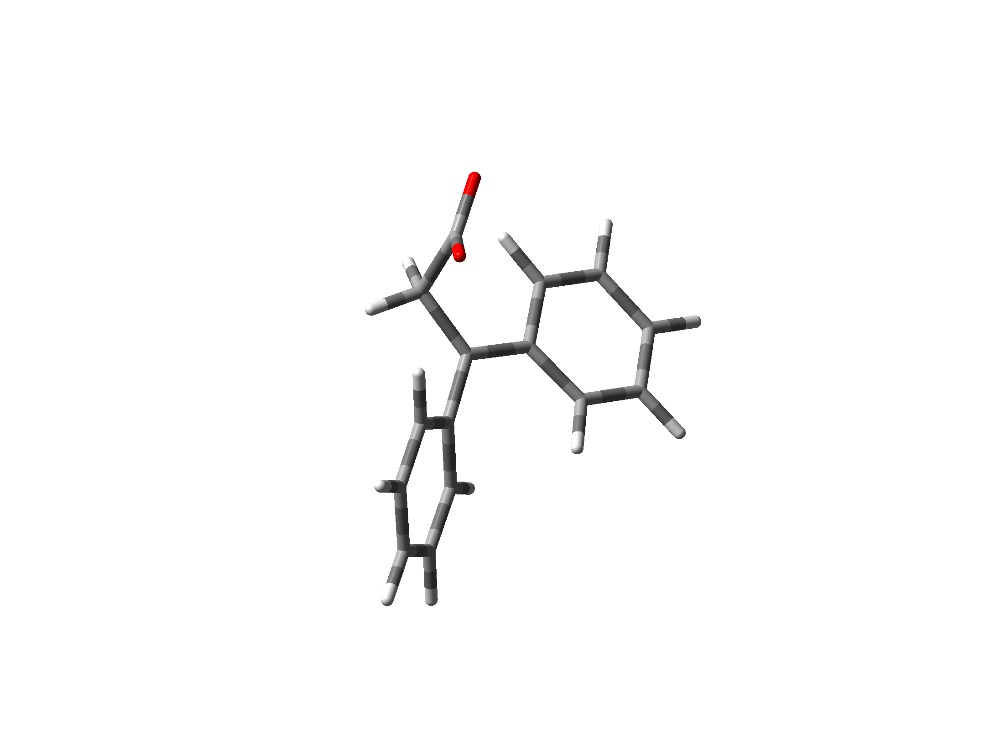
**

**1,1-DPE(RA)-COOβ:** 1,1-Diphenylethylene radical anion carboxylate product in β position

**
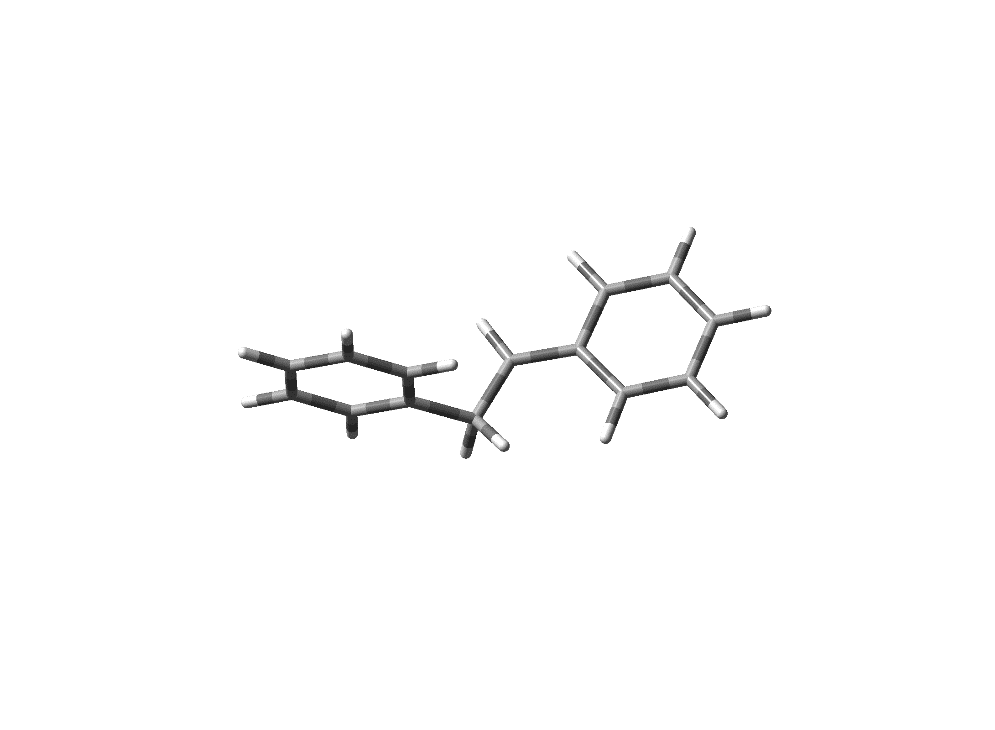
**

**S(RA)-H**: E-Stilbene radical anion conjugate acid

**
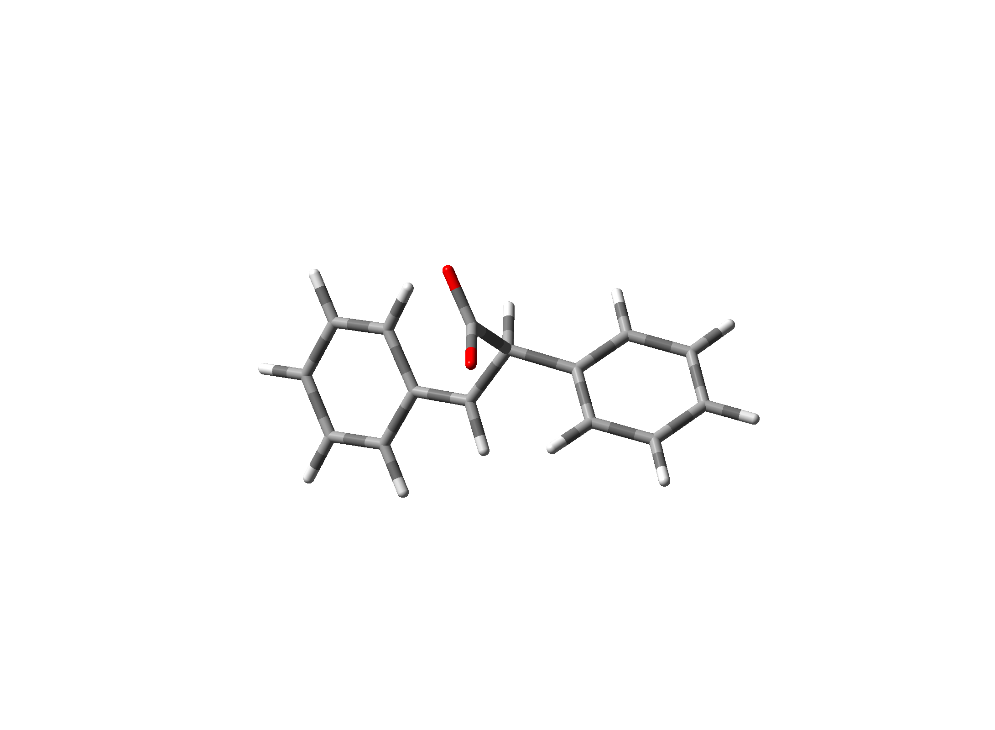
**

**S(RA)-COO**: E-Stilbene radical anion carboxylate product

**
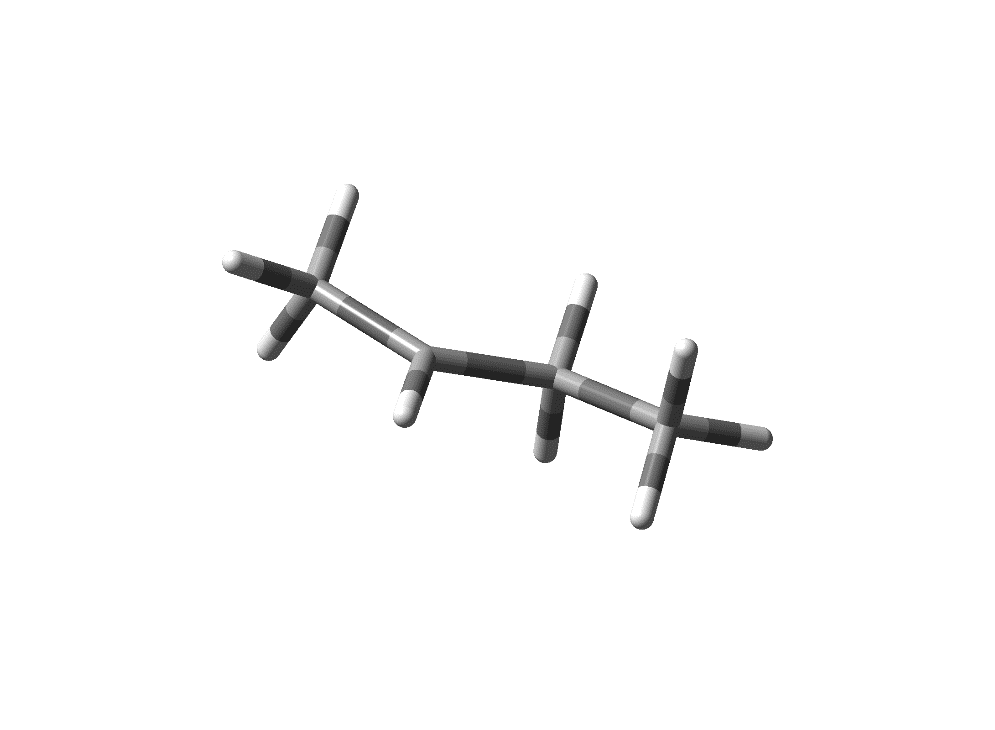
**

**B(RA)-H***: trans*-2-Butene radical anion conjugate acid

**
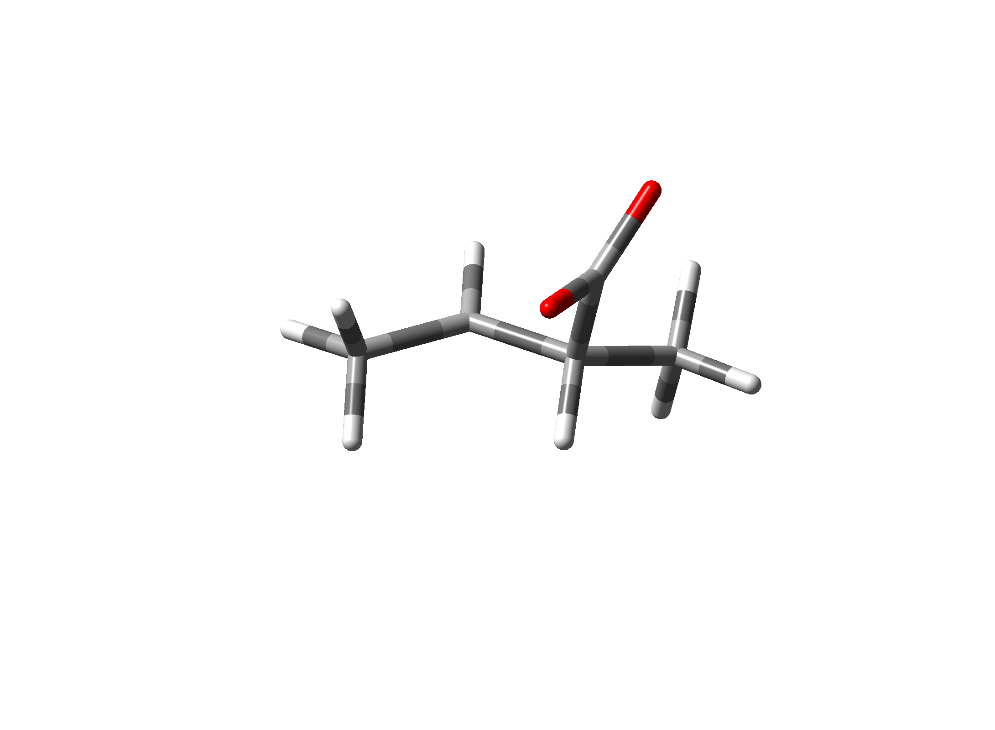
**

**B(RA)-COO***: trans*-2-Butene radical anion carboxylate product

**
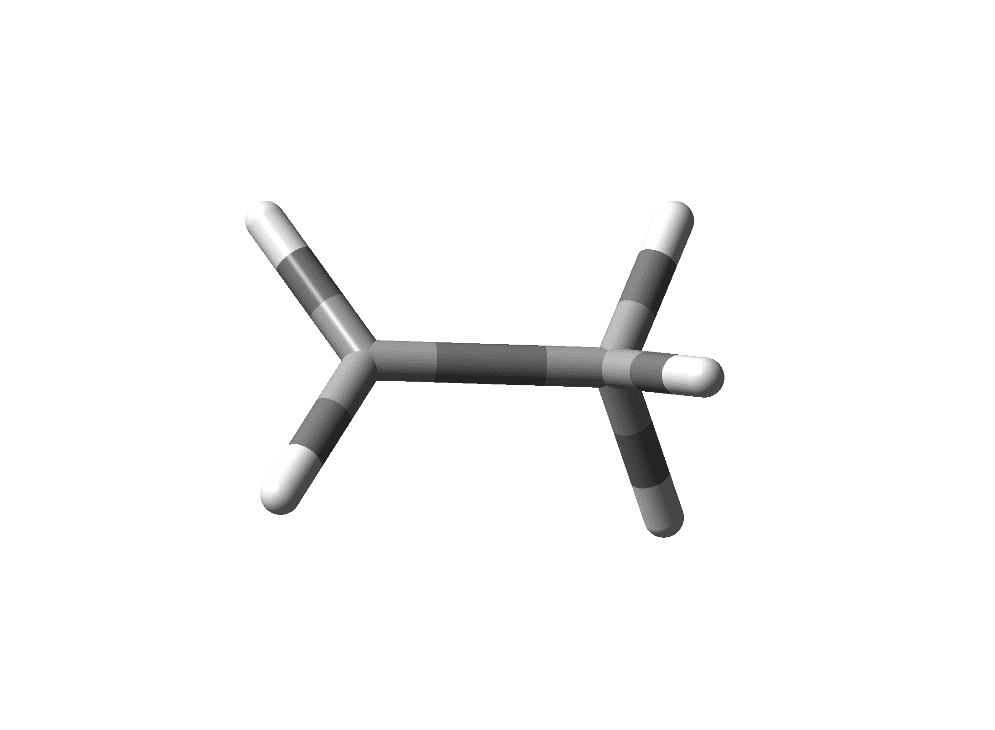
**

**E(RA)-H:** Ethylene radical anion conjugate acid

**
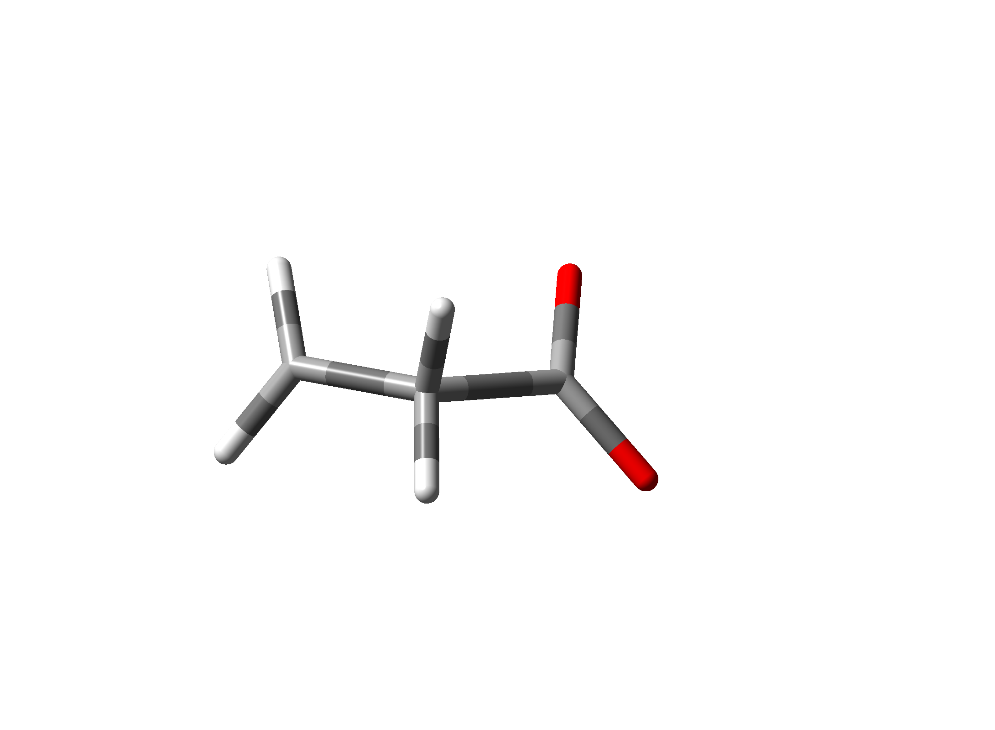
**

**E(RA)-COO**: Ethylene radical anion carboxylate product

**
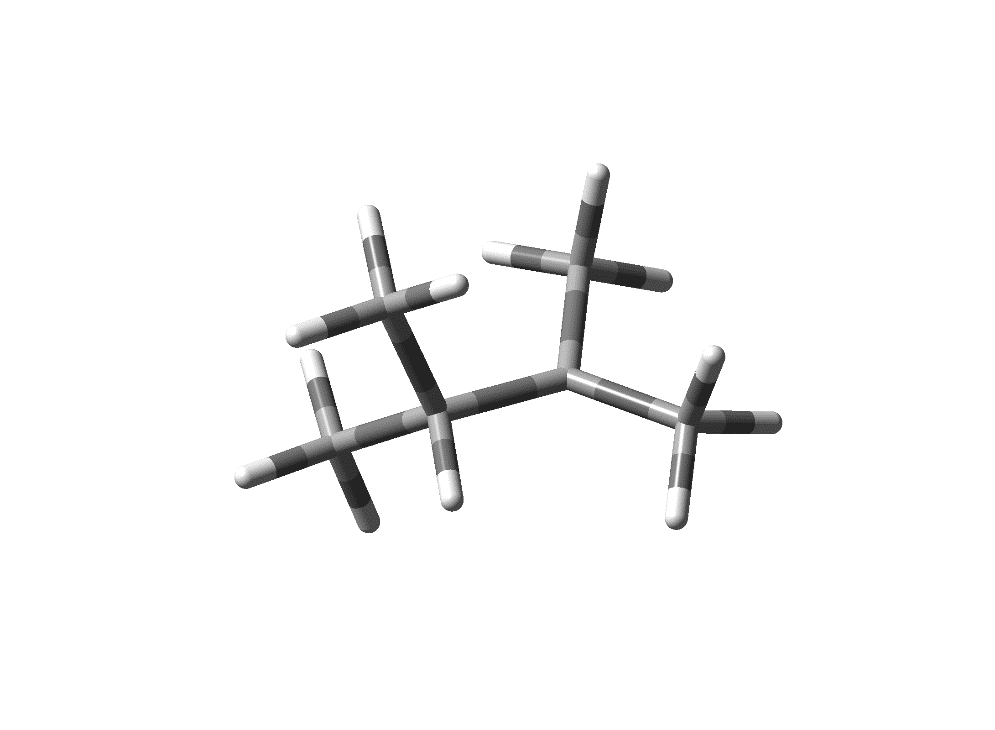
**

**T(RA)-H:** Tetramethylethylene radical anion conjugate acid

**
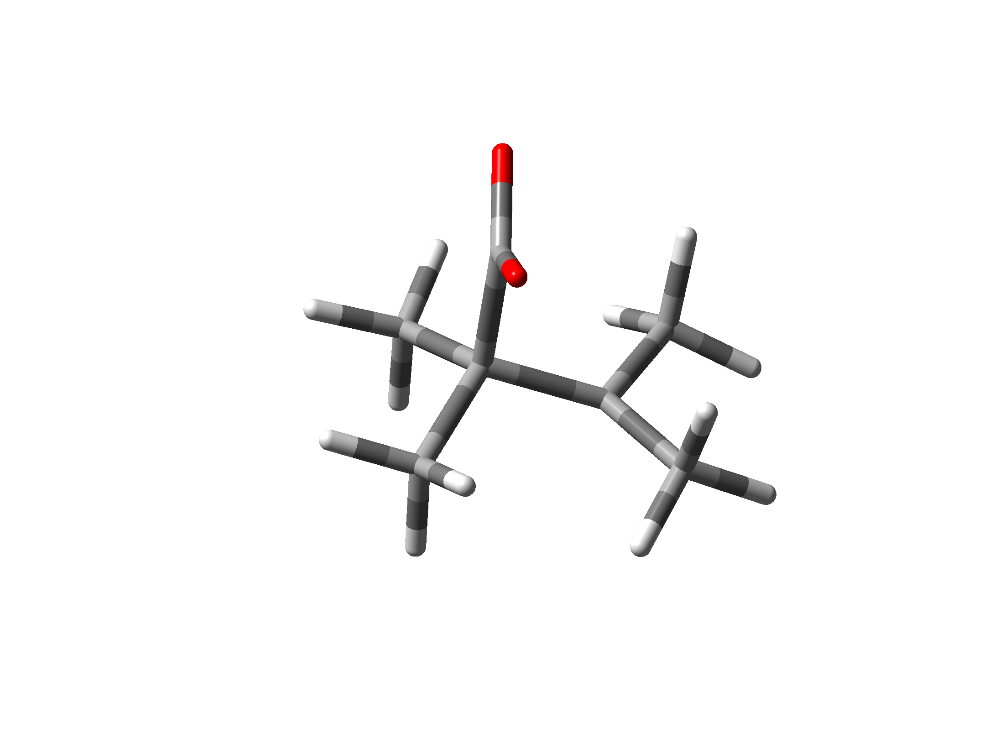
**

**T(RA)-COO**: Tetramethylethylene radical anion carboxylate product

**
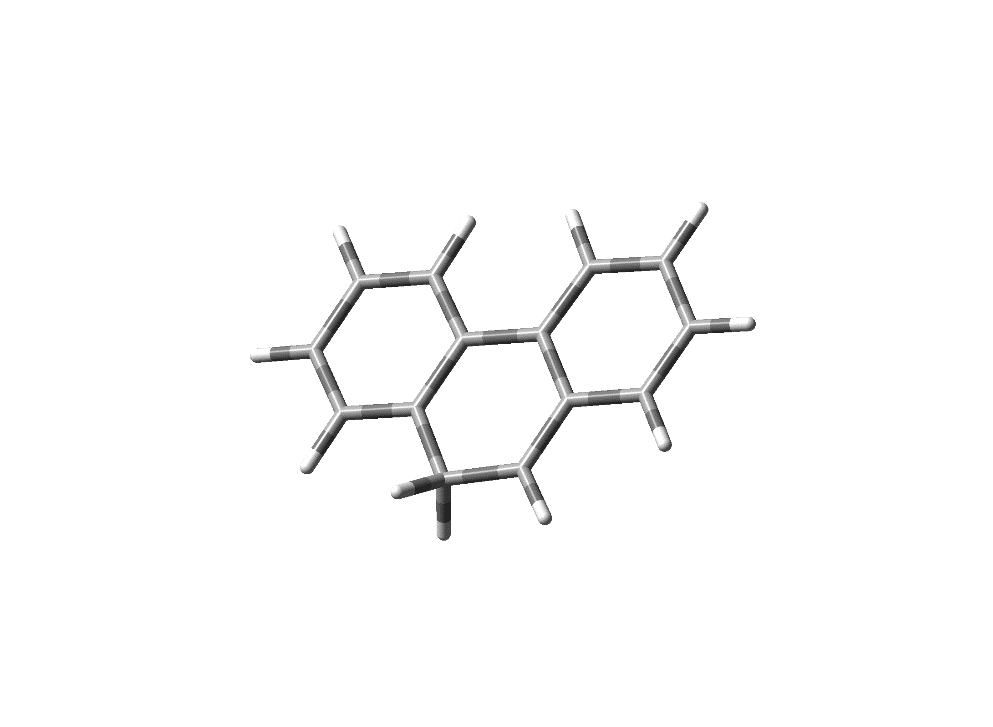
**

**P(RA)-H**: Phenanthrene radical anion conjugate acid

**
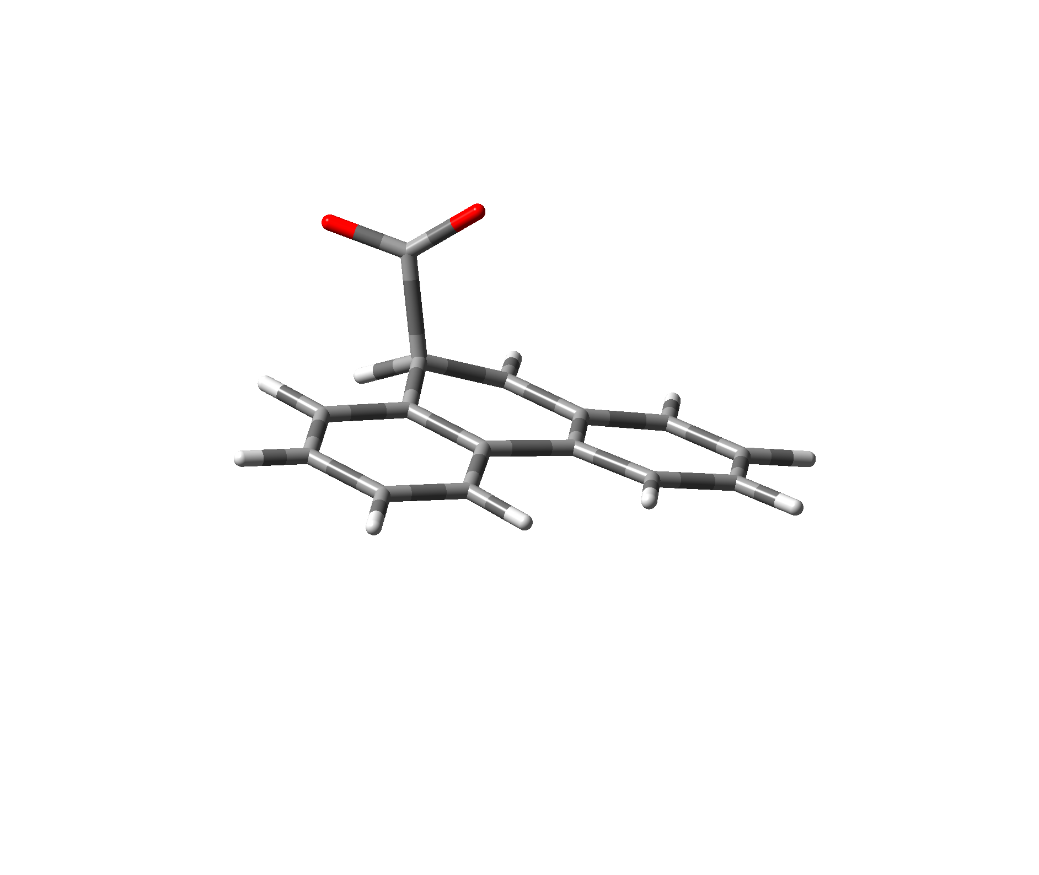
**

**P(RA)-COO:** Phenanthrene radical anion carboxylate product


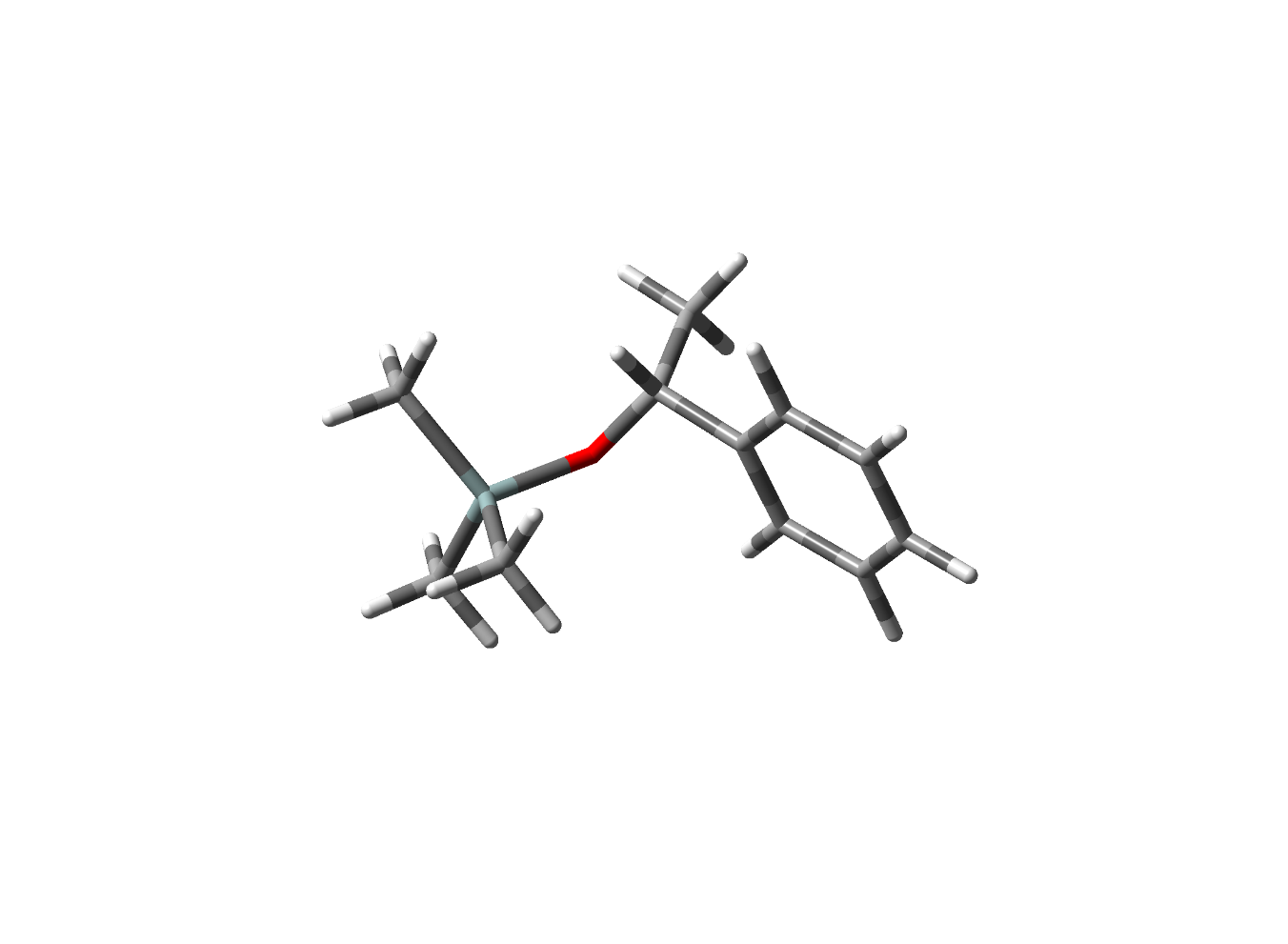


**ACP-OTMS(A)-H**: Acetophenone trimethylsilyl [−Si(CH_3_)_3_] protected anion conjugate acid


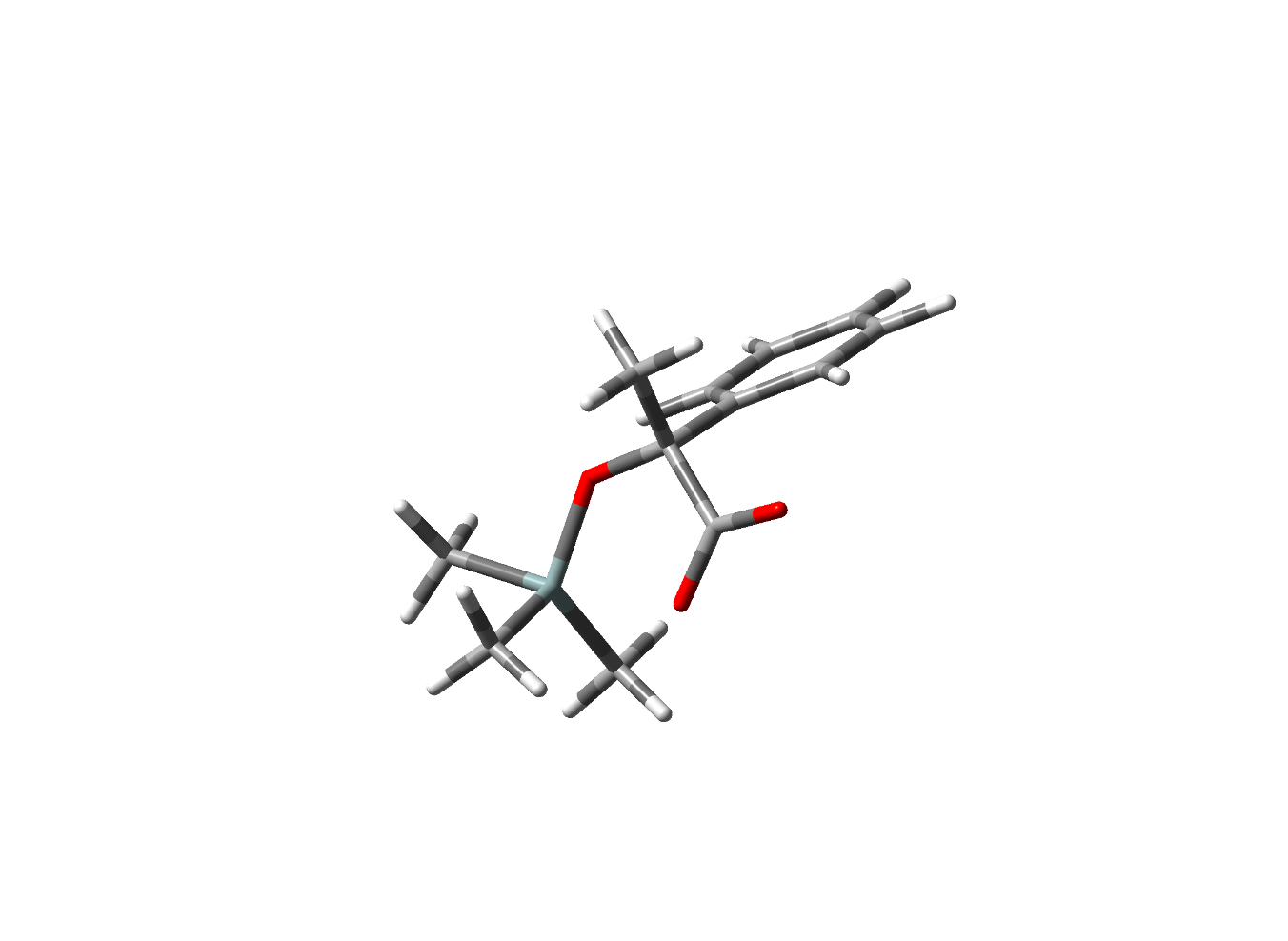


**ACP-OTMS(A)-COO**: Acetophenone trimethylsilyl [−Si(CH_3_)_3_] protected anion carboxylate product


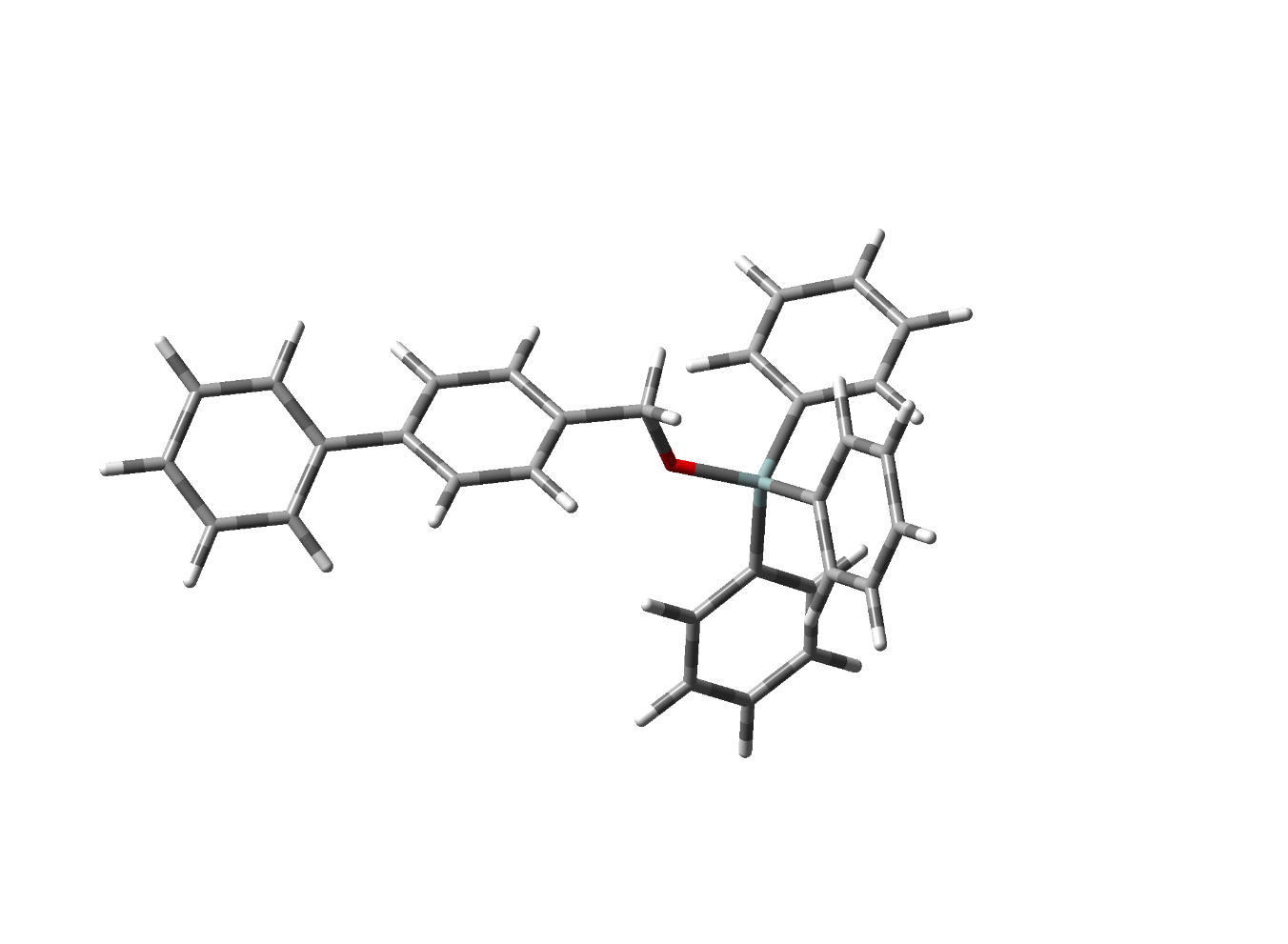


**pPBA-OTPS(A)-H:** *para*-phenilbenzaildeide triphenylsilyl [−Si(CH_3_)_3_] protected anion conjugate acid

**
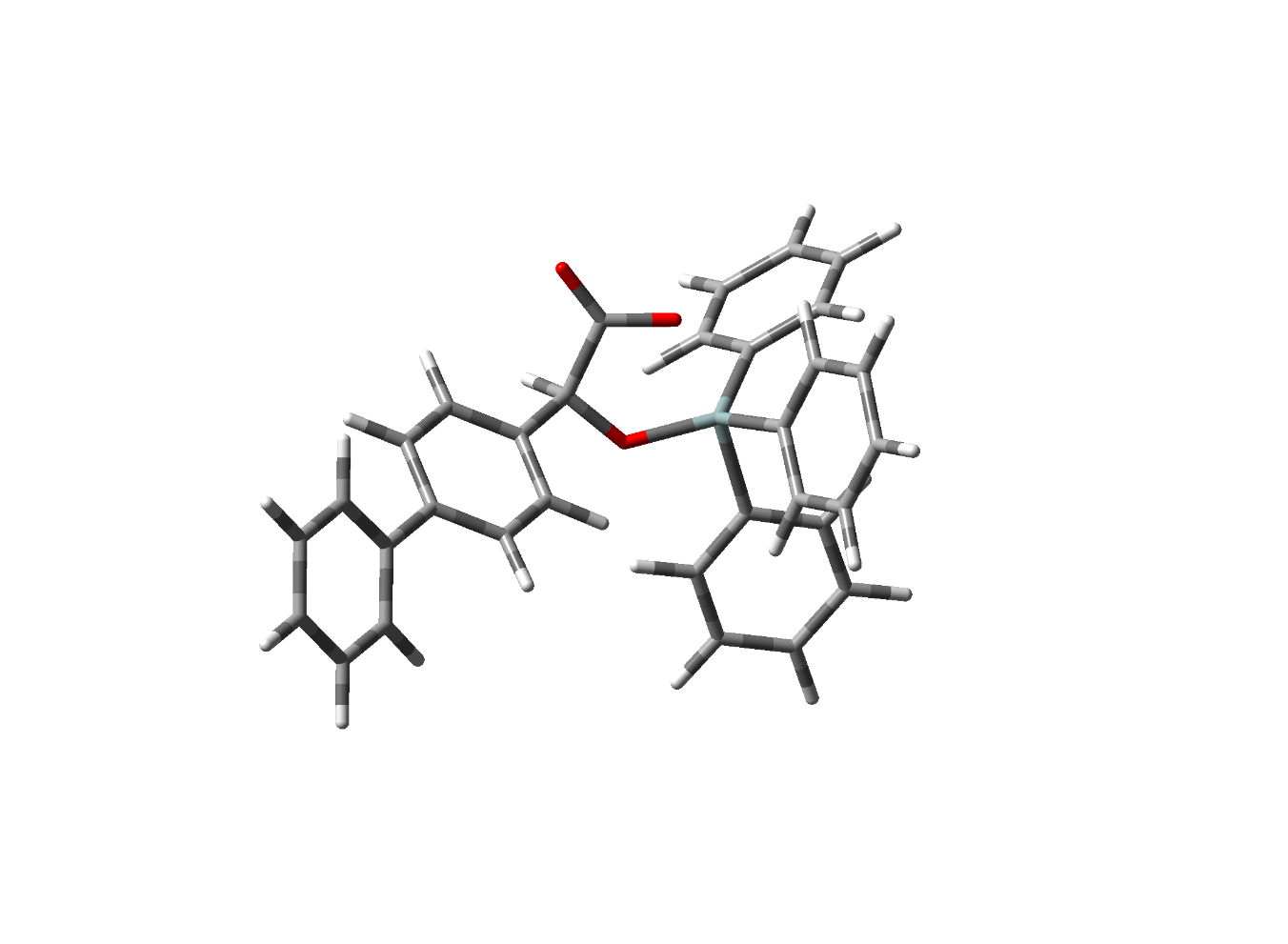
**

**p-PBA-OTPS(A)-COO:** *para*-phenilbenzaildeide triphenylsilyl [−Si(CH_3_)_3_] protected anion carboxylate product


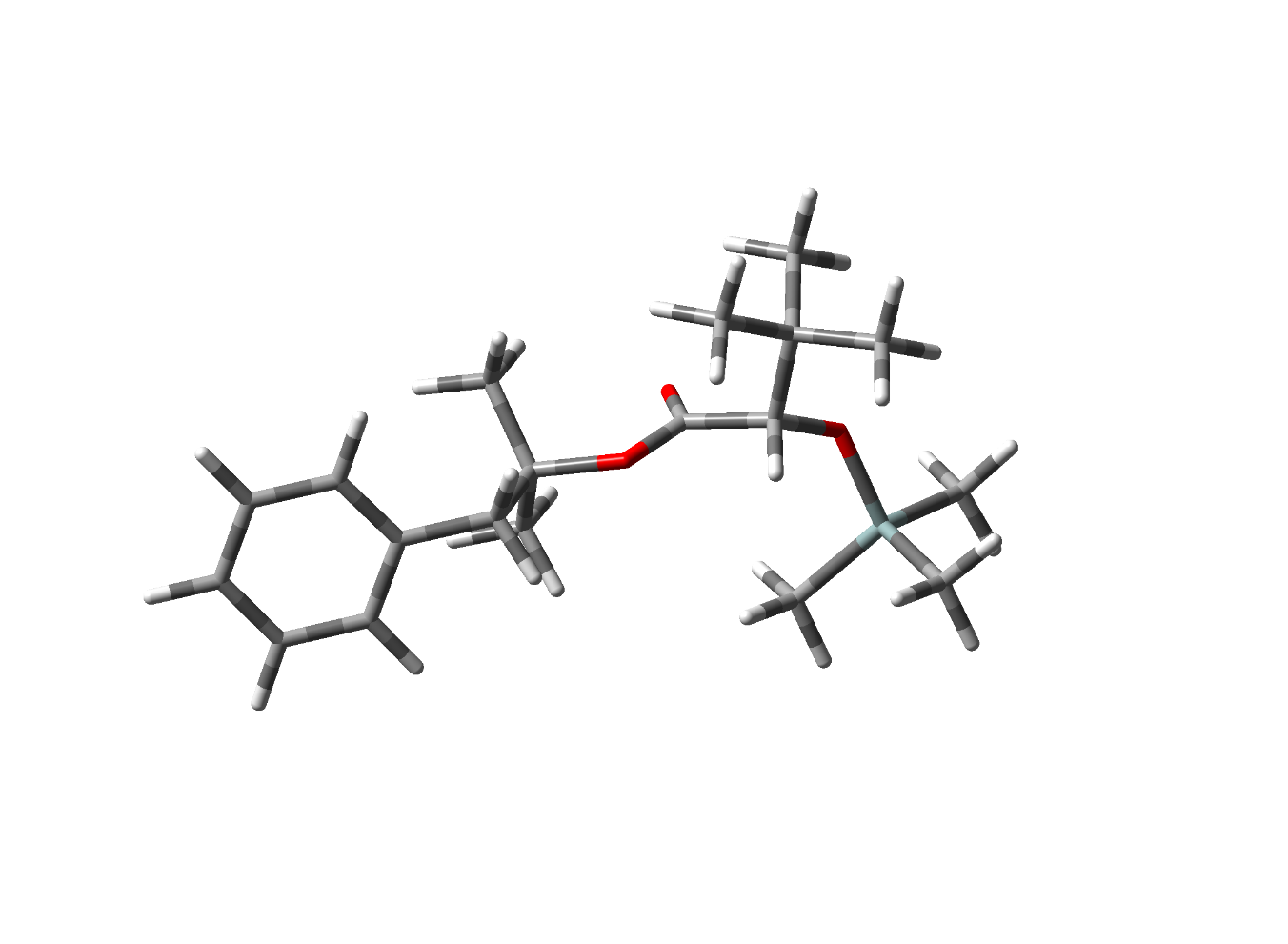


**TMPE-OTMS(A)-H:** 1-phenyl-2-methylpropil (trimethyl)pyruvate triphenylsilyl [−Si(CH_3_)_3_] protected anion conjugate acid


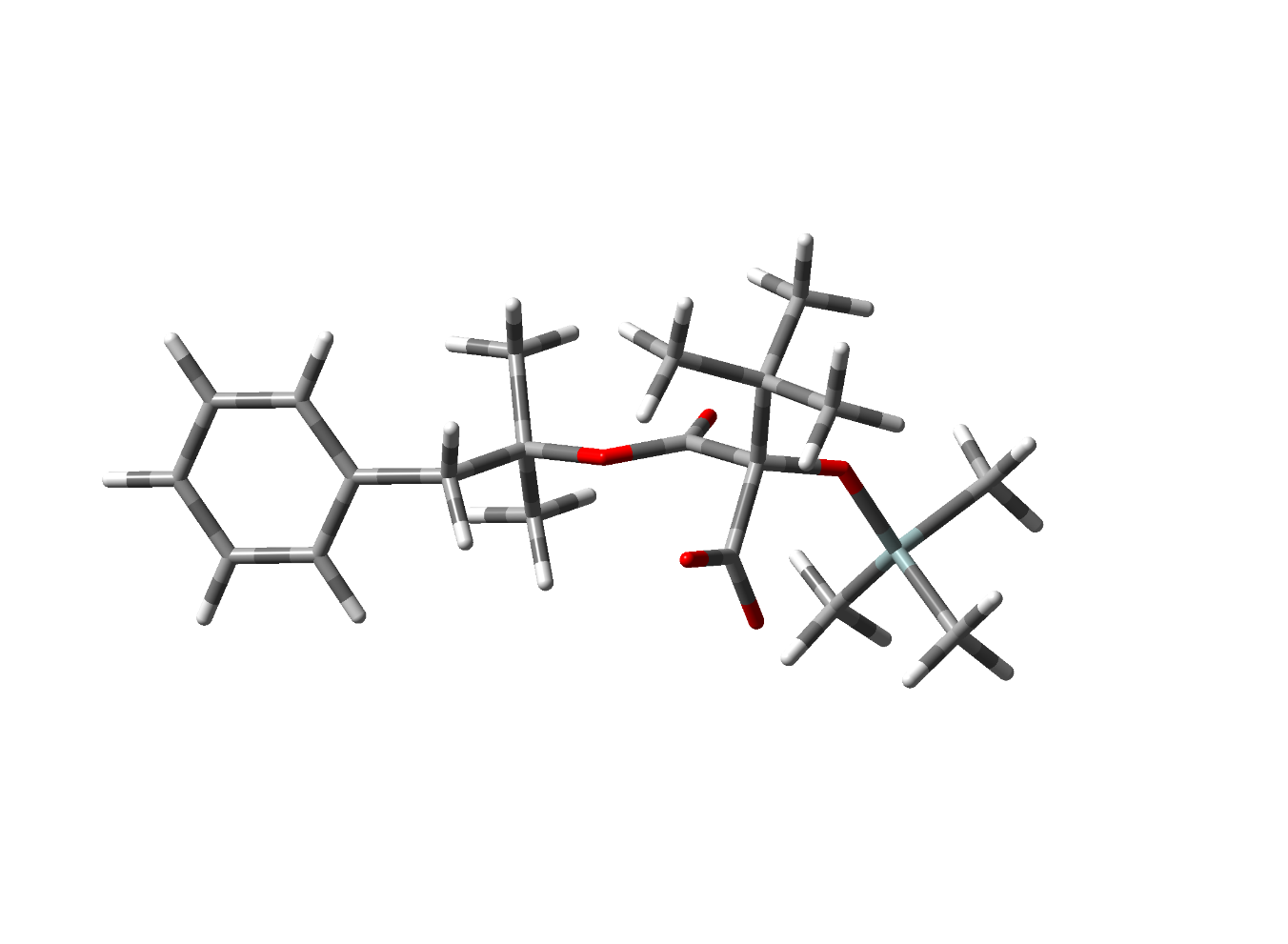


**TMPE-OTMS(A)-H:** 1-phenyl-2-methylpropil (trimethyl)pyruvate triphenylsilyl [−Si(CH_3_)_3_] protected anion carboxylate product


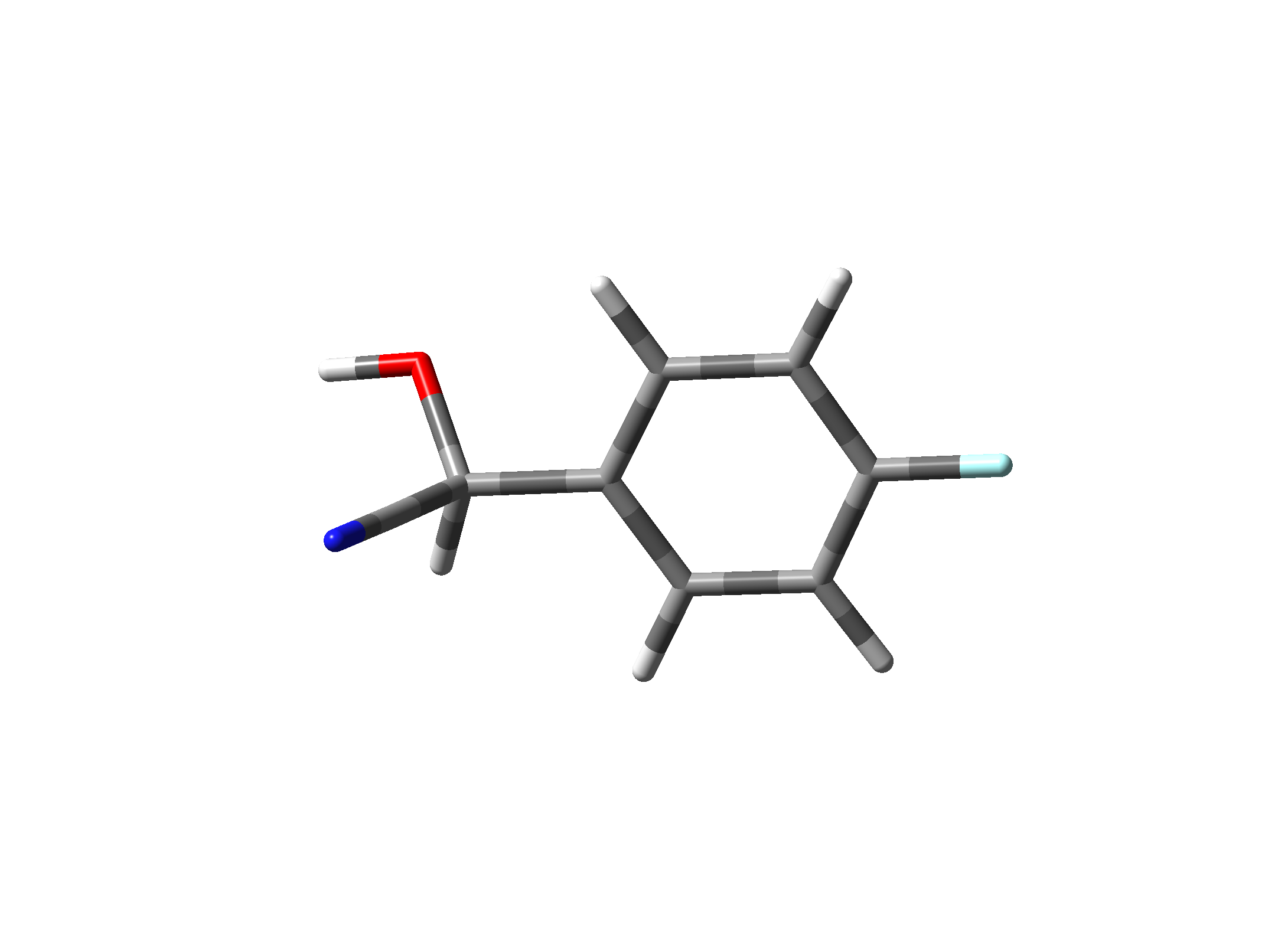


**p-FM:** *para*-fluoro-mandelonitrile


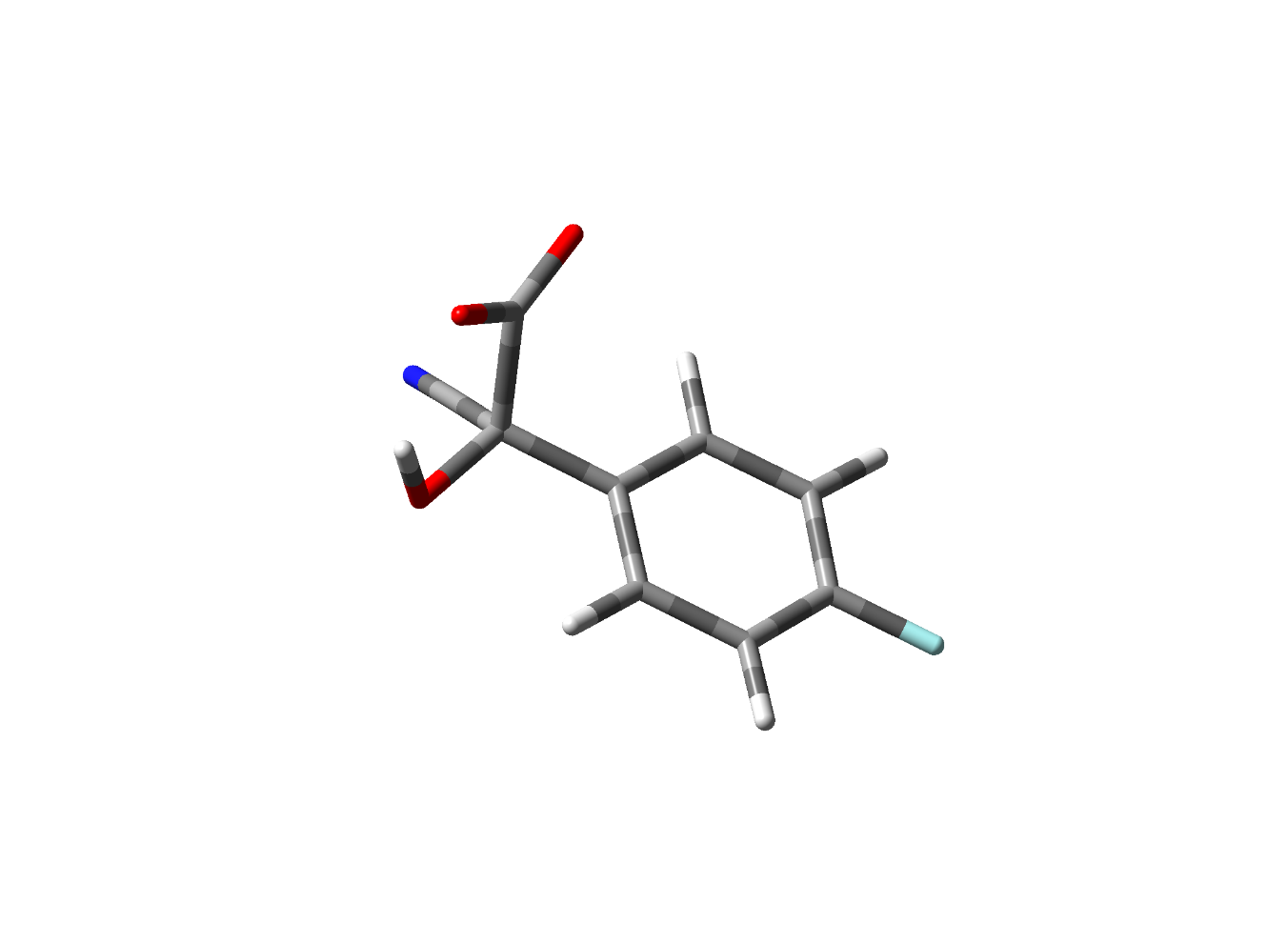


**p-FM:** *para*-fluoro-mandelonitrile anion carboxylate product

# G. References

1 R. R. Gagne, C. A. Koval. and G. C. Lisensky, *Inorg. Chem.*, 1980, **19**, 2854–2855.
